# Capturing structural intermediates in an animal-like cryptochrome photoreceptor by time-resolved crystallography

**DOI:** 10.1126/sciadv.adu7247

**Published:** 2025-05-16

**Authors:** Manuel Maestre-Reyna, Yuhei Hosokawa, Po-Hsun Wang, Martin Saft, Nicolas Caramello, Sylvain Engilberge, Sophie Franz-Badur, Eka Putra Gusti Ngurah Putu, Mai Nakamura, Wen-Jin Wu, Hsiang-Yi Wu, Cheng-Chung Lee, Wei-Cheng Huang, Kai-Fa Huang, Yao-Kai Chang, Cheng-Han Yang, Meng-Iao Fong, Wei-Ting Lin, Kai-Chun Yang, Yuki Ban, Tomoki Imura, Atsuo Kazuoka, Eisho Tanida, Shigeki Owada, Yasumasa Joti, Rie Tanaka, Tomoyuki Tanaka, Jungmin Kang, Fangjia Luo, Kensuke Tono, Stephan Kiontke, Lukas Korf, Yasufumi Umena, Takehiko Tosha, Yoshitaka Bessho, Eriko Nango, So Iwata, Antoine Royant, Ming-Daw Tsai, Junpei Yamamoto, Lars-Oliver Essen

**Affiliations:** ^1^ Department of Chemistry, National Taiwan University, 1Roosevelt Rd. Sec. 4, Taipei 106, Taiwan.; ^2^ Institute of Biological Chemistry, Academia Sinica, 128 Academia Rd. Sec. 2, Nankang, Taipei 115, Taiwan.; ^3^ Division of Chemistry, Graduate School of Engineering Science, Osaka University, 1-3 Machikaneyama, Toyonaka, Osaka 560-8531, Japan.; ^4^ Department of Chemistry, Philipps University Marburg, Hans-Meerwein Strasse 4, Marburg 35032, Germany.; ^5^ European Synchrotron Radiation Facility, 38043 Grenoble, France.; ^6^ Hamburg Centre for Ultrafast Imaging, Universität Hamburg, 22761 Hamburg, Germany.; ^7^ Univ. Grenoble Alpes, CNRS, CEA, Institut de Biologie Structurale (IBS), 38044 Grenoble, France.; ^8^ RIKEN SPring-8 Center, 1-1-1 Kouto, Sayo, Hyogo 679-5148, Japan.; ^9^ Japan Synchrotron Radiation Research Institute, 1-1-1 Kouto, Sayo, Hyogo 679-5198, Japan.; ^10^ Department of Cell Biology, Graduate School of Medicine, Kyoto University, Yoshidakonoe-cho, Sakyo-ku, Kyoto 606-8501, Japan.; ^11^ AICHI SR Center, Nagoya University, 250-3 Minamiyamaguchi-cho, Seto-shi, Aichi 464-8603, Japan.; ^12^ School of Science, University of Hyogo, 3-2-1 Kouto, Kamigori-cho, Ako-gun, Hyogo 678-1297, Japan.; ^13^ Graduate School of Medical Life Science, Yokohama City University, 1-7-29 Suehiro, Yokohama, Kanagawa 230-0045, Japan.; ^14^ Institute of Multidisciplinary Research for Advanced Materials, Tohoku University, 2-1-1 Katahira, Aoba-ku, Sendai 980-8577, Japan.; ^15^ Institute of Biochemical Sciences, National Taiwan University, 1, Roosevelt Rd. Sec. 4, Taipei 106, Taiwan.

## Abstract

Animal-like cryptochromes are photoreceptors that control circadian rhythm and signaling in many eukaryotes. Transient photoreduction of the cryptochrome flavin chromophore initiated signaling via a poorly understood mechanism. By serial femtosecond crystallography (SFX), we show that the photoreduction mechanism of *Chlamydomonas reinhardtii* cryptochrome involves three loci [carboxyl-terminal region, a transient protonation pathway, and flavin adenine dinucleotide (FAD)–binding site] acting in unison to accomplish three effects: radical pair stabilization, protonation of FAD radical, and formation of the signaling state. Using 19 time-resolved SFX snapshots between 10 nanoseconds and 233 milliseconds, we found that light-driven FAD^•–^/tyrosyl-373 radical pair (RP) formation primes α22 unfolding. Electron transfer–dependent protonation of aspartate-321 by tyrosine-373 is the epicenter of unfolding by disrupting salt bridges between α22 and the photolyase homology region. Before helix unfolding, another pathway opens transiently for FAD^•–^ protonation and RP stabilization. This link between RP formation and conformational changes provides a structural basis for signaling by animal-like cryptochromes.

## Teaser

Time-resolved crystallography unveils how light-driven chromophore reduction sparks dramatic structural change in a photoreceptor.

## INTRODUCTION

Cryptochromes (CRYs) belong to the photolyase/cryptochrome superfamily (PCSf), a group of flavin-dependent photoreceptors, which evolved repeatedly from photolyases as animal-like (aCRY), plant-like (pCRY, CryP), and DASH-type CRYs (*1*). Biological activity by CRY depends mostly on light-driven electron transfer (ET) to their flavin adenine dinucleotide (FAD) chromophore (*2*–*4*). While photolyases catalyze light-driven DNA repair (*5*, *6*), CRYs modulate plant growth, regulate circadian rhythms (*3*), and may even act as magnetoreceptors (*7*–*10*). As evolutionary transitional forms, some CRYs like the aCRY from *Chlamydomonas reinhardtii* (*Cr*aCRY) are bifunctional by acting both as photoreceptor (*11*–*13*) and photolyase (*14*).

**Fig. 1.**
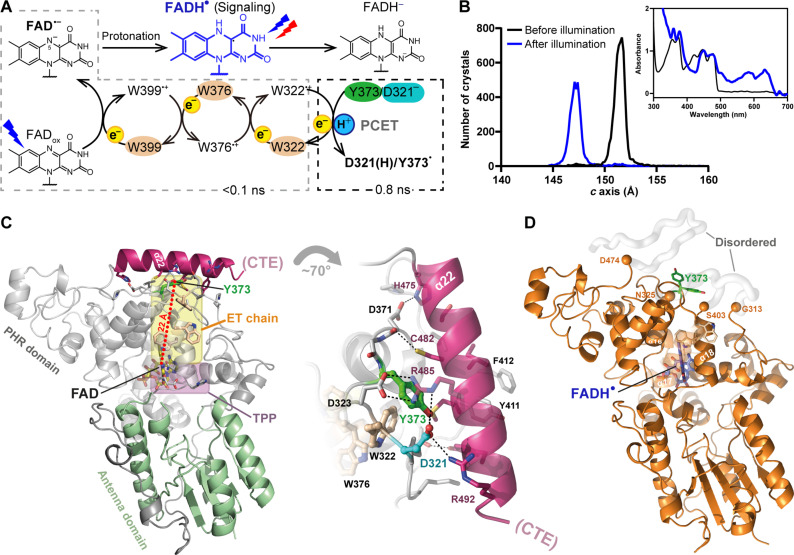
*Cr*aCRY photochemistry and redox-dependent SFX structures. (**A**) FAD_ox_ photoreduction generates the FAD^•–^/Y373^•^ RP by ET from Y373 (dashed boxes) within less than 1 ns, whereas PT to FAD^•−^ (top) is pH dependent and proceeds slower in the sub-second time range (*21*). Further reduction to FADH^−^ allows *Cr*aCRY to act as (6-4) photolyase (*14*). (**B**) Distribution plots of *c*-axis unit cell length of *Cr*aCRY crystals before (black) and after illumination (blue). A change of unit cell parameters accompanies light-triggered FAD➔ FADH^•^ transition of *Cr*aCRY crystals [inlay, ultraviolet visible (UV/Vis) spectra before and after illumination]. The oxidized and fully reduced *Cr*aCRY crystals are isomorphous (table S1). (**C**) Left, *Cr*aCRY structure in FAD_ox_ state with pathways for ET (yellow) and PT (TPP, pink) within the PHR domain (gray). The D321/Y373 PCET switch is marked in cyan/green, the α22 helix in magenta. Right, α22 helix/PHR domain interface with hydrogen bonding/ionic interactions shown as dashed lines. Our *Cr*aCRY structures miss the intrinsically disordered C-terminal extension (CTE) common to CRYs. (**D**) *Cr*aCRY structure in FADH^•^ state (orange). Orange spheres mark start and end points of disordered regions (gray tubes). Notably, Y373 (green) is ill defined by adopting multiple conformations.

*Cr*aCRY uses a tetrad of four aromatic residues (*14*–*16*) for FAD photoreduction and subsequent formation of the signaling-relevant FADH^•^ state (*11*). Like in other PCSf members of the aCRY/(6–4) photolyase branch (*17*, *18*), these residues (*Cr*aCRY: W399, W376, W322, and Y373) form a 22-Å-long ET pathway from the surface of the C-terminal photolyase homology region (PHR) domain toward the FAD chromophore. In *Cr*aCRY, light-driven ET forms within less than a nanosecond, the FAD^•–^/Y373^•^ radical pair (RP), where final ET from Y373 to W322^•+^ is coupled to proton transfer (PT) from Y373 to D321 (Fig. 1A). Subsequently, the FAD^•–^/Y373^•^ RP is further stabilized by protonation of FAD^•–^ to FADH^•^ (*15*, *16*). The molecular mechanism of flavin protonation remains unclear. As with other aCRYs (*19*), the very late stages of the *Cr*aCRY photocycle involve a large-scale C-terminal unfolding event that occurs several seconds after illumination (*20*). The structural determinants leading to this order-disorder transition are not well understood. With a sequence identity of 61% for the PHR, *Cr*aCRY is highly related to CRYs like European robin (*Erithaculus rubecula*) CRY4a. It has been hypothesized that CRY4a may be the bird’s compass for migration because its photoinduced RP is affected by relatively weak magnetic fields (2 mT) (*9*). *Cr*aCRY is therefore an excellent model system to study aCRY behavior as it is a highly representative member of this PCSf subfamily. Technically, in addition to having a well-characterized photocycle and a high quantum yield of ∼60% (*21*), *Cr*aCRY produced very robust, strongly diffracting crystals. Both of these features greatly facilitated detailed analysis of its structural kinetics via time-resolved serial femtosecond crystallography (TR-SFX) (*22*), an emerging method that takes advantage of ultrafast x-ray pulses produced by x-ray Free Electron Lasers (XFEL) (*23*–*25*) to produce detailed, structural, three-dimensional (3D) movies of protein function.

In the following, we used our *Cr*aCRY model to show the mechanisms of (a) initial RP stabilization, (b) protonation of the FAD^•–^ radical, and (c) how photoreduction of an aCRY causes structural changes in its C-terminal region 22 Å away from its FAD chromophore to foster potential down-stream signaling. Light-triggered strain within the D321/Y373 switch causes disordering of helix α22 on a millisecond time scale, which occurs in competition with a reordering reaction. Meanwhile, fast rotation of N395 nearby FAD within 10 ns resulted in both stabilization of FAD^•–^, and slower activation of a transient protonation pathway (TPP) consisting of E384 and H309 leading up to formation of FADH^•^ in ~40 ms. In summary, here, we provide a detailed structural-kinetic framework for conformational changes in an aCRY, having isolated all major structural intermediates between RP formation and signaling state.

## RESULTS

### Damage-free structures show partial unfolding of *Cr*aCRY in its FADH^•^ state

Without external reductant, FAD_ox_➔FADH^•^ photoreduction of *Cr*aCRY stalls in the semiquinoid FADH^•^ state (*14*). One end of the ET pathway (Fig. 1A), the deprotonated tyrosyl radical Y373^•^, requires chemical reduction to act again as electron donor for the second, light-driven reduction to the catalytically active FADH^−^ state (*14*). Furthermore, Y373^•^ has rather long lifetimes of 26 ms and 2.6 s when derived from the FAD_ox_ and the FADH^•^ state, respectively (*15*, *16*). To resolve redox-dependent changes in *Cr*aCRY, we used damage-free SFX (*26*) to derive static structures in all three redox states (Fig. 1B and table S1): FAD_ox_ (dark, Fig. 1C), photoreduced FADH^•^ as required for signaling (Fig. 1D and fig. S1A), and the fully reduced FADH^−^ state, i.e., *Cr*aCRY’s catalytically competent form as photolyase (fig. S1B).

*Cr*aCRY structures in FAD_ox_ and FADH^−^ states at room temperature are almost identical to those at cryogenic conditions (*14*), with root mean square deviation values of 0.235 and 0.212 Å for 488 Cα positions, respectively. Accordingly, they share the highly conserved two-domain topology of other PCSf members (*14*) with the FAD chromophore residing in the PHR domain, and Y373, the terminal electron donor, being centrally embedded between the canonical PHR domain and the C-terminal helix α22 (Fig. 1C and fig. S1). In contrast, the semiquinoid FADH^•^ state of *Cr*aCRY shows large, light-dependent disordering (Fig. 1D), which is reflected by changing crystal lattice dimensions due to collapse of the lattice pocket that harbored helix α22 in the oxidized state (Fig. 1B, fig. S2, and table S1). An increase of disorder is also manifested in solution according to H/D exchange and native mass spectrometry (MS) data (fig. S3) (*20*, *27*).

In *Cr*aCRY’s FADH^•^ state, helix α22 (H475-K494) and two regions nearby, N314-D324 in the α13/α14 loop as well as A404-Q410 in the α18/α19 loop, are disordered because of lacking electron density (Fig. 1D). Consequently, the ET pathway along W399-W376-W322-Y373 to the FAD chromophore (Fig. 1C) is broken. First, the Y373 side chain, which packs closely against W322 in FAD_ox_ and FADH^−^ states (Fig. 1C), is released from any interactions by becoming completely solvent exposed (Fig. 1D). Secondly, W322 is indispensable like Y373 for photoreduction (*16*) but belongs now to the disordered α13/α14 loop. Moreover, the FAD_ox_➔FADH^•^ transition causes loss of the prominent salt bridges between α22 and the PHR domain (Fig. 1C), namely D321-R485/R492, D323-R485 and D371-H475. Again, aspartates D321 and D323 belong to the disordered α13/α14 loop, while Y373 gets mobile and adopts two alternative conformations (Fig. 1D and fig. S1C). The aromatic residues Y411, F412, and Y415 of the α18/α19 loop, which contact helix α22 in the FAD_ox_ and FADH^−^ states, reorder and form an alternate set of interactions (Fig. 1C and fig. S1C). Y411 and F412 pack instead with L358 and H361 of the FAD-binding site, respectively, and cause conformational changes for these residues contacting the isoalloxazine’s methyl groups (fig. S1C). Local effects around the FADH^•^ chromophore are otherwise minor, i.e., less pronounced than found for photochemical reactions catalyzed by a class II photolyase (*26*, *28*), but consistent with TR-SFX studies of the (6-4) photolyase from *Drosophila melanogaster* (*29*). For example, the isoalloxazine moiety of FADH^•^ and FADH^−^ remains planar. Only the Oδ1 atom of N395 approaches the N5 nitrogen of FAD’s isoalloxazine moiety (fig. S1) to stabilize the protonated FAD chromophore (*26*, *30*, *31*).

The order-disorder transition upon FADH^•^ state formation is unusual as a surface area of 870 Å^2^ from 14 α22 residues is occluded from solvent access by interacting with the PHR domain (Fig. 1C). This resembles the well-known α helix unfolding found for an unrelated class of flavin photoreceptors, the Jα helix comprising light oxygen voltage (LOV) domains (*32*). However, the distance between the chromophore and helix α22 of *Cr*aCRY is 25 Å, almost twice as long as for the Jα helix of LOV domains (14 Å).

*Cr*aCRY’s FADH^•^ state structure provides numerous additional interaction sites for downstream signaling partners like the circadian clock factor ROC15 (*33*). Our in crystallo observation of a partly unfolded FADH^•^ state prompts us to note that refolding to an FAD_ox_-like conformation, concomitant with restoration of the ET pathway from Y373 to the chromophore, is required for further FADH^•^➔FADH^−^ photoreduction. In solution, a transient nature of the *Cr*aCRY signaling state is corroborated by kinetic native MS studies giving upper boundaries of 8.1 and 4.6 s for light-driven *Cr*aCRY unfolding and refolding, respectively (*20*).

### Tracking the conformational consequences of FAD_ox_ ➔ FADH^•^ photoreduction by TR-SFX

Next, we wondered (a) what kind of structural change precedes *Cr*aCRY’s order-to-disorder transition after RP formation and (b) whether a pathway exists for chromophore protonation in the FAD^•−^ state. The latter is essential to minimize fast nonproductive recombination of the FAD^•−^/Y373^•^ RP (*21*). However, *Cr*aCRY structures of all redox states lack such a protonation pathway leading from solvent to the FAD chromophore’s N5 nitrogen. This occlusion of FAD’s N5 from solvent access is not only found for *Cr*aCRY but also a general feature of structurally characterized PCSf members. To address these issues, we collected TR-SFX data using 3-ns laser pump pulses at a wavelength of 450 nm at the Spring-8 Angstrom Compact free electron LAser (SACLA). Experimental details, power titrations, etc. are described in Materials and Methods, texts S1 to S3, figs. S4 and S5, and tables S1 to S4. Given the subnanosecond rates for photochemical and ET reactions (*21*), our setup focuses on TR-SFX snapshots of *Cr*aCRY’s conformational changes after accomplished FAD^•−^/Y373^•^ RP formation.

**Fig. 2.**
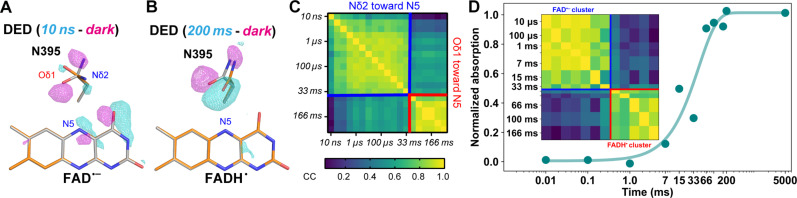
Stabilization of FAD radicals. (**A** and **B**) TR-SFX snapshots of the FAD-binding site [(A) 10 ns and (B) 200 ms] showing 3.5σ contoured DED (time-dark) maps (positive peaks, cyan; negative, magenta), overlaid on snapshot (orange) and FAD_ox_ state (dark, gray) structures. (**C**) Correlation map for N395 DED features. Pair-wise correlation coefficients (CC) between N395 features are color-coded in the heatmap. Two regions of high correlation emerge, one spanning from 10 to 33 ms (blue square), the other from 66 to 233 ms (red). Each region marks a different N395 to FAD orientation, i.e., Nδ2 toward N5 (blue) and Oδ1 toward N5 (red). (**D**) Time-resolved in crystallo UV/Vis spectra of the FAD^•−^➔FADH^•^ transition. The time trace shows accumulation of FADH^•^ (blue), fitted with a first-order kinetic. The inset panel displays a correlation map of spectra collected up to 166 ms after illumination, divided into two regions. The first one, dominated by FAD^•−^, spans from 10 μs to 33 ms, while the second, enriched with FADH^•^, lasts from 66 to 166 ms. For representative individual spectra, refer to figs. S12 (C and D).

**Fig. 3.**
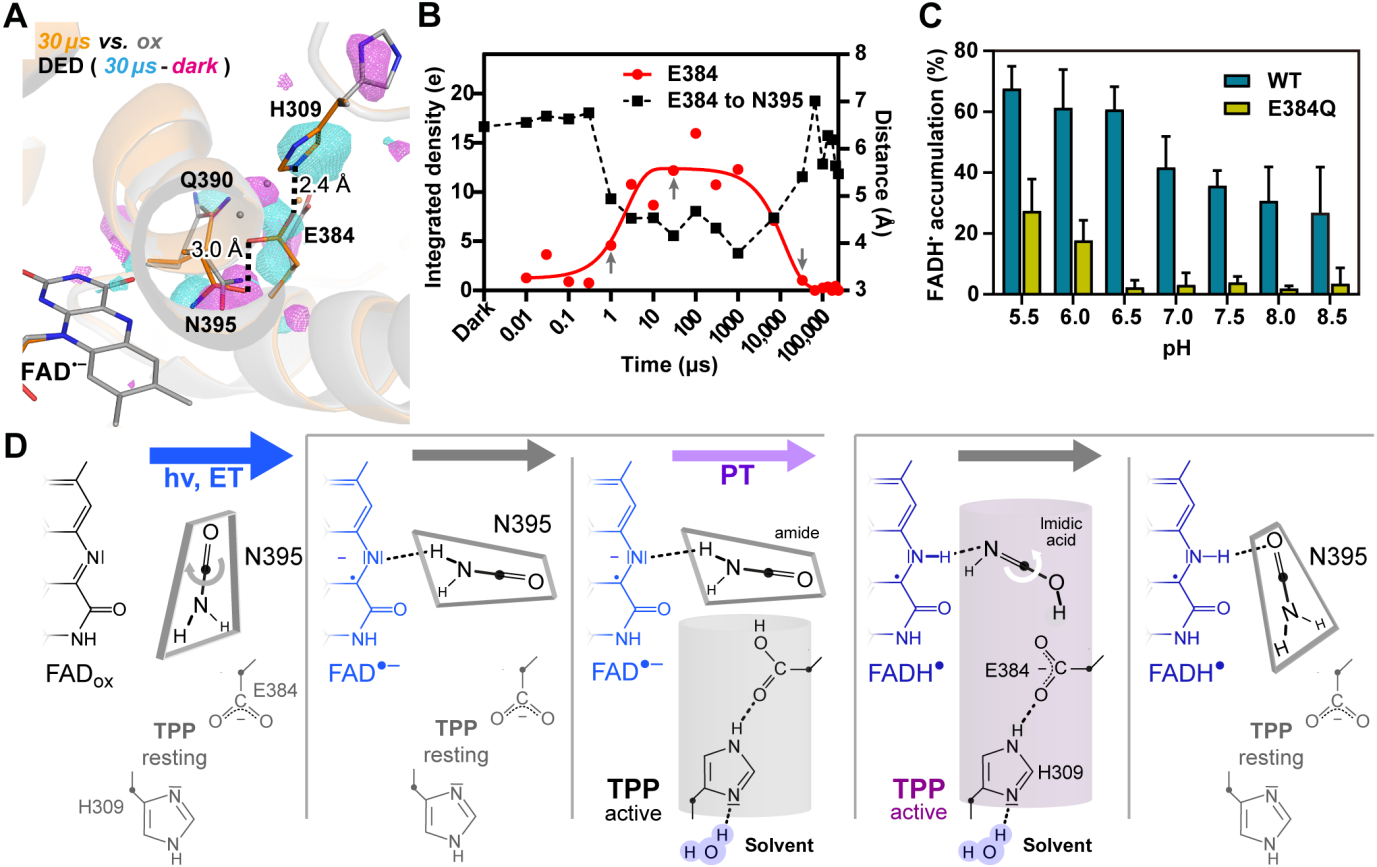
The transient protonation pathway. (**A**) Detail of transient protonation pathway (TPP) connecting bulk solvent to FAD via N395. The 30-μs snapshot (orange) with its 3.5σ contoured DED map represents TPP changes when compared to the FAD_ox_ state (dark, gray). (**B**) E384-to-N395 center of mass distances (black squares) compared to time-dependent accumulation of DED around E384 (red dots). DED data were fitted to a two-step kinetic model (continuous red line) for rate constants. The time points for TPP onset (1 μs), full activation (30 μs), and ending (33 ms) are indicated by arrows. (**C**) pH-dependent photoreduction of *Cr*aCRY wild type (WT, blue) and E384Q (lime green). Photoreductions were performed at different pH values to monitor the sensitivity of photoreduction to proton concentration. (**D**) The N395-trigger model for the TPP. Here, N395 mediates TPP activation after ~1 μs but also PT to FAD^•−^ by amide/imidic acid transition of its carboxamide side chain.

**Fig. 4.**
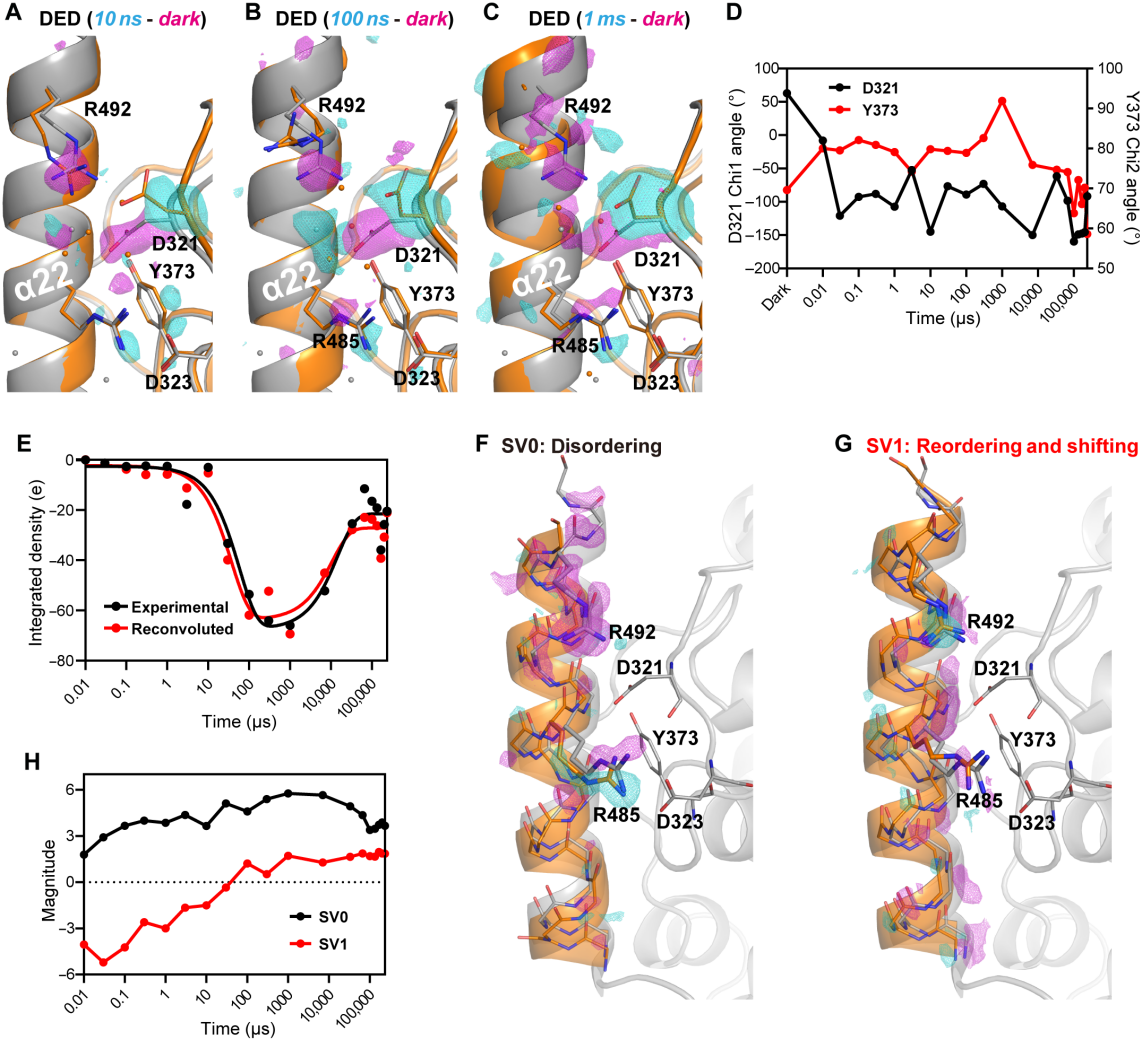
α22/PHR interface with D321/Y373 PCET switch. (**A** to **C**) TR-SFX snapshots of α22/PHR interface (orange) at 10 ns (A), 100 ns (B), and 1 ms (C) with corresponding 3σ contoured DED (time-dark) maps. DED signals concentrate early around D321, while later DED peaks spread around helix α22. The FAD_ox_ state (dark, gray) is shown as reference. (**D**) Time-dependent conformational changes of the D321/Y373 switch. (**E**) Time-dependent negative DED evolution around α22 (black dots) fits to a two-step kinetics model (continuous black line). Reconvoluted DED derived from the two main SVD components (red dots) matches well with experimental data and follows the same kinetics (continuous red line). (**F** and **G**) Time-invariant SVD components SV0 (F) and SV1 (G) appear as difference maps along *Cr*aCRY’s C-terminal region. To illustrate protein dynamics, SVD structural models (orange) (*70*) are shown (files S1 and S2). (**H**) Time-dependent traces of SV0 (black) and SV1 (red) evolution. SV0 gradually increases in the early time points, but decays later. Meanwhile SV1 is initially negative, while changing sign after ~30 μs.

When analyzing snapshots evolving from the RP via difference electron density (DED) maps, we observed time-dependent differences in three major regions (Fig. 1C and fig. S6): the FAD-binding site (Fig. 2 and figs. S7 and S8), a patch adjacent to the FAD pocket that we assigned as TPP (Fig. 3 and fig. S9), and helix α22 including its interface to the PHR domain (Fig. 4 and fig. S10). Next, each of these regions will be addressed in detail.

### FAD-binding site—The N395/FAD switch

Ultrafast spectroscopic studies on *Cr*aCRY showed that photoexcited FAD^*^ abstracts within <0.4 ps an electron from its neighbor, W399 (*21*). The resulting electron hole at W399^•+^ hops then via aromatic residues of the ET pathway toward Y373, with the slowest step being proton-coupled ET (PCET). PCET (τ_PCET_ = 0.8 ns) involves ET between W322 and Y373 and concomitant PT within the hydrogen-bonded D321/Y373 pair (*21*). Accordingly, peaks around FAD’s isoalloxazine moiety appear in early DED maps (10 ns-dark) (Fig. 2A). These slight changes within the first 3 μs (figs. S8 and S11) correspond to relaxation of the isoalloxazine’s geometry after ET. Before they return to the common conformations in the static *Cr*aCRY structures, they are accompanied by slight, compensatory movements of the highly conserved R360-D389 salt bridge packing against the FAD^•−^ isoalloxazine group (fig. S8).

In the FAD-binding site, the strongest DED peaks appearing during the entire time course correspond to N395’s rotating carboxamide group (Fig. 2, A and B, and fig. S7). DED correlation analysis of N395 peaks (Fig. 2C) reveals two distinct patterns with a transition occurring between the 33- and 66-ms snapshots, which we assign to the interaction between N395 and either FAD^•−^ or FADH^•^ radicals. Between 10 ns and 33 ms, a clockwise rotation relative to the dark state (Δchi2: −86.4°) positions the N395 side chain within hydrogen-bonding distance between its Nδ2 atom and the FAD^•−^ N5 nitrogen (Fig. 2A). Between 66 and 233 ms, a counter-clockwise rotation almost restores the dark-state conformation (Δchi2: +111°) of this N395/FAD switch by forming now a hydrogen bond between the protonated N5 nitrogen of FADH^•^ and the N395 Oδ1 atom. As suggested by previous reports for the conserved counterparts of N395 in other CRYs (*26*, *30*, *31*), the conformational flip of this asparagine stabilizes the FAD radicals. To confirm radical flavin species in crystallo over the entire time course, we performed time-resolved in crystallo optical spectroscopy, TR-icOS (Fig. 2D and fig. S12). TR-icOS shows that illumination of *Cr*aCRY crystals at a variety of power levels results in FAD^•–^ protonation ~45 ms after illumination (τ_FADH•_ = 43.9 ms), which correlates closely with the kinetics of N395 conformational changes (Fig. 2, A to C, and figs. S5 and S12). Furthermore, correlation analysis of TR-icOS data (Fig. 2D) produces a remarkably similar pattern to N395 DED correlation map (Fig. 2C), strongly supporting that FAD protonation is concomitant with N395 reorientation.

### Transient protonation pathway

Next to the FAD, DED peaks appear adjacent to the N395/FAD switch for a solvent-exposed patch of residues containing H309, E384, and Q390 (Fig. 3A and fig. S9). Our set of TR-SFX snapshots reveals that the side chain of E384 starts to orient toward N395 1 μs after photoreduction (Fig. 3B and fig. S9). This is accompanied by H309 side-chain swiveling, which is hydrogen-bonded from the 30-μs snapshot onward via its Nε2 nitrogen to Oε2 of E384 (2.4 Å, Fig. 3A and figs. S9 and S13), as well as compensatory changes contributed by the Q390’s side chain. This conformational transition as triggered by N395 places the Oε1 atom of the E384 carboxyl group over the plane of the N395 carboxamide with distances of 3.0 Å (Oδ1) and 3.9 Å (Nδ2), i.e., geometrically improper to form additional H bonds. This updated network of interactions leading from the solvent via H309 to N395 lasts until ~33 ms and is disrupted again by return to a dark state–like conformation in the 66-ms snapshot (Fig. 3B and figs. S9 and S13, τ_H309_ = 23 ms, τ_E384_ = 13 ms). To further address E384, we performed steady-state photoreduction assays for the E384Q mutant in solution (Fig. 3C). Unlike wild type, E384Q is inefficient to generate the neutral radical FADH^•^ at physiological pH, i.e., between 6.5 and 8.5, and can be recovered only to ~41% of wild type activity at acidic pH 5.5 (Fig. 3C). Accordingly, we suggest that E384 acts as shuttle for the essential PT between solvent and the FAD^•–^ radical.

Given these transient changes, we propose that the dyad H309/E384 is part of TPP that causes conversion of FAD^•−^ to FADH^•^ in *Cr*aCRY. In this scenario (Fig. 3D), clockwise rotation of the N395 side chain is enforced by anionic FAD^•−^ and activates TPP by H309/E384 reorientation onset after 1 μs. In the fully active state (>30 μs), a continuous protonation pathway forms between solvent and the E384 carboxyl group. The final hurdle is PT from E384 to the FAD^•−^ N5 nitrogen via N395. In analogy to the short-lived PCET reactions in blue light using FAD (BLUF) photoreceptors with their inbuilt flavin/glutamine/tyrosine triad (*34*), we imagine that only a slight rotation of N395 is now required to allow O-protonation of the N395 carboxamide by E384 with concomitant PT from the carboxamide’s nitrogen to N5 (Fig. 3D). However, unlike BLUF photoreceptors, where the glutamine’s imidic acid has long enough lifetimes to stabilize a signaling state (*35*), we predict that an N395 imidic acid tautomer is rather transient in *Cr*aCRY due to retautomerization upon TPP inactivation and N395 rotation. Given that the active TPP state lasts for at least 33 ms and final PT along E384-N395- FAD^•−^ is predictably much faster, this step may not be kinetically resolvable by TR-SFX.

### D321/Y373 PCET switch triggers α22 helix unfolding

As outlined above, α22 interacts in the FAD_ox_ state with the PHR via hydrophobic contacts and salt bridges, particularly D321-R492 and D323-R485. Our earliest time point at 10 ns after PCET demonstrates the unique role of D321 as proton acceptor (*21*) as the most prominent DED signals belong to this residue (Fig. 4A and fig. S10). PCET obviously triggers a flip of the D321 side chain that lasts over the entire time course. This PCET switching in the D321/Y373 couple causes release of the protonated D321 side chain from ionic interaction with R492 and hydrogen bonding to R485 (Figs. 1C and 4A). Solvent exposure and a lack of interaction partners further increase flexibility of the D321 side chain. Concomitantly, negative DED signals for the R492 side chain accumulate early at 10 ns. As no positive DED peaks are associated with R492, our structural snapshots show progressively increased flexibility as indicated early by multiple conformers for R492 (100 ns, Fig. 4B), which becomes thereafter completely disordered (Fig. 4C and fig. S10). Water invades a cavity between α22 and the PHR domain that is opened by D321-R492 salt bridge disruption, as evidenced by a large positive DED peak in the α22/PHR interface at 100 ns (Fig. 4B). Otherwise, PCET within the D321/Y373 switch causes only minor effects to Y373 that transiently rotates its phenolic group by subtle chi2 angle changes from 69.6° (dark) to 80° (10 ns) before recovering its dark-state conformation after 7 ms (Fig. 4D).

After R492 disordering, negative DED peaks rise (τ_α22,disorder_ = 59 μs) along α22’s C-terminal end (Fig. 4, B, C, and E). These signals suggest here an increased disorder and flexibility of α22 end’s residues over time. Although in our snapshots, α22 residues never returned to their original positions for the entirety of the time course, DED signals subside after ~10 ms, which suggest partial reordering (τ_α22,reorder_ = 15 ms; Fig. 4E).

We used singular value decomposition (SVD) of DED maps around α22 to resolve remaining discrepancies between our sets of structural snapshots and DED maps (Fig. 4, F to H). As reconvolution of only the first two SVD principal components is sufficient to reproduce the observed integrated DED time trace (Fig. 4E), we propose that these two explain α22 dynamics during the late stages of *Cr*aCRY photoreduction: The main component, SV0, corresponds to α22 disordering characterized by accumulation of negative DED around the α22 end (Fig. 4F). It also comprises disordering of R492 and conformational change of R485. SV0 dominates the entire time course (Fig. 4H), gradually increasing its magnitude until 100 μs, and only slowly subsiding from that point on. Meanwhile, under the effects of SV1 and beyond 30 μs, α22 adopts an alternative conformation in which the helix shifts away from the PHR domain, and both R485 and R492 are well ordered (Fig. 4G). Since SV1 magnitudes contribute positively in the later stages of our time course (Fig. 4H), we believe that it is reasonable to describe it as a long-lived intermediate in a competing reordering process, in opposition to further disordering promoted by SV0. On the basis of this interpretation, we anticipate the yield of disordered α22 to be lower than the efficiency of ET between FAD and Y373, as not every single successful ET event triggers α22 unfolding. In other words, the photoreduction yield of ~20%, which was estimated from XFEL data extrapolation based on DED signals in the neighborhood of D321 (tables S1 and S2), is diminished to ~10% for the α22 order-disorder transition.

In summary, SVD analysis reveals that α22 initial disordering is slow and without intermediates. Meanwhile, the competing reordering process, which ensues during the later stages of our time course, follows more traditional protein-folding kinetics with a structural intermediate described by SV1.

## DISCUSSION

### TPP intermediates and C-terminal strain in *Cr*aCRY: Implications for the PCSf

**Fig. 5.**
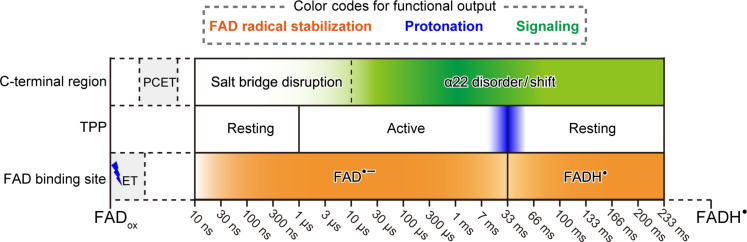
Schematic overview of *Cr*aCRY 3D molecular movie. Three loci (C-terminal region, TPP, and FAD-binding site) act in unison to accomplish three effects, RP stabilization (orange), protonation of FAD radical (blue), and formation of the signaling state (green). In the scheme, color gradients along each locus’ timeline represent the extent to which they contribute to each effect at any given time. On the left, a blue jagged line represents the blue light pulse initiating the *Cr*aCRY photocycle.

Before, we and others produced 3D-molecular movies by TR-SFX for photolyases undergoing photoreduction (*26*, *29*) or catalyzing DNA repair (*28*, *36*). Our SFX data for an aCRY, *Cr*aCRY, show now two sites of action triggered by light-driven RP formation. Each of them can be a prerequisite for downstream signaling by this and other aCRYs (Fig. 5 and movie S1): First, the N395/FAD switch activates TPP with concomitant FAD protonation, whereas, secondly, structural strain derived from the D321/Y373 PCET switch primes slow α22 helix unfolding.

As a key residue, N395 of the N395/FAD switch is highly conserved in most PCSf subfamilies, i.e., eukaryotic (6-4) and class I cyclobutane pyrimidine dimer (CPD) photolyases, DASH CRYs and NewPHL, because its position controls and modulates FAD redox chemistry (*3*, *14*, *30*, *31*). This asparagine is only replaced by aspartate in plant-type CRYs (*37*), water in prokaryotic (6-4) photolyases (*38*), and cysteine in a subbranch of the aCRYs (*39*). In *Cr*aCRY, we observe that N395’s clockwise rotation activates a TPP after about 1 μs. Subsequent FAD^•−^ protonation allows rotation reversal with concomitant TPP deactivation (Fig. 3D). What may be the benefit of a transient rather than a permanently active protonation pathway for PCSf members? First, the occlusion from solvent access of anionic FAD^•−^ and FADH^−^ species—the latter being crucial for photolyase activity—protects the chromophore against reoxidation by rapid oxygen diffusion. Notably, in insect CRYs, the replacement of asparagine by a smaller cysteine goes along with a highly stabilized FAD^•−^ signaling state (*39*). Secondly, one may envision that local changes involving the TPP are recognized by interaction partners. In this context, light-independent CRYs from mouse demonstrate that, when being complexed with the circadian clock factor PER2, the region corresponding to the TPP in *Cr*aCRY interacts with a PER2 helix (*40*, *41*). Furthermore, the C-terminal helix of these mammalian CRYs is counterpart of *Cr*aCRY’s α22 helix and packs not only against the remainder of PER2 but also to the FBXL3 component of the SCF_FBXL3_ ubiquitin ligase (*42*).

The structure-based kinetics of *Cr*aCRY protonation by TPP reveals how conformational changes in bird CRYs may depend on magnetoreception despite notable structural and mechanistic differences. The proposed mechanism for avian CRY magnetoreception involves preequilibrium kinetics of the photoinduced RP as depicted in fig. S14 (*7*, *8*, *10*, *43*). Upon illumination, the RP is initially spin correlated (fig. S14) because, before reaching equilibrium, it exhibits a bias toward either the singlet or triplet states. Concurrently with singlet/triplet equilibration over time, only singlet RPs can recombine by back ET to the dark-adapted state, while both singlet and triplet can evolve further via some reaction, such as protonation, into the signaling state (fig. S14). As singlet/triplet equilibration is affected by the RP’s orientation relative to magnetic fields, the proportion of CRY reaching the signaling state is controlled by the magnetic field as well (*7*, *8*, *43*). Simulations suggest that geomagnetic field sensitivity requires RP spin correlation to persist for ~1 μs (*8*), with much longer RP lifetimes after loss of spin correlation causing a loss of magnetic field sensitivity. We note that the N395/FAD switch aligns with these kinetic requirements by displacing the E384 side chain from its dark state conformation after 1 μs, thereby triggering TPP activation. Activated TPP drives PT to FAD^•−^, forming FADH^•^, and may also serve as a potential site for light-dependent CRY-effector interactions. Thus, the TPP couples FAD protonation to the RP dynamics and signaling within the proposed magnetosensory timescale of avian CRYs.

Notably, the evolution of FAD^•−^ toward FADH^•^ commits the C-terminal region to α22 helix unfolding. Consequently, the N395/FAD switch provides a mechanism by which spin-correlated RP, active on the microsecond timescale, could influence the likelihood of slower, large-scale conformational change (movie S1 and Fig. 5). Nevertheless, the order-disorder transition of α22 is initiated by a different site, the D321/Y373 PCET switch. First, this switch expels D321 from its interaction network within the α22/PHR interface; secondly, sets on disordering; and, finally, unfolding of helix α22. Unlike TPP activation, order-disorder transition of α22, due to electrostatic strain within the D321/Y373 switch, is partly reversible as we observe some recovering of the initial state within the time range of FAD chromophore protonation. Recent time-resolved ion mobility MS experiments on full-length *Cr*aCRY revealed that unfolding involving the α22 helix occurs on a timescale of seconds (*20*). This positions even our latest time-resolved snapshots (233 ms) within the early stages of the order-disorder transition. The kinetic reversibility during these early stages accounts for the disparity between our later time-resolved snapshots (Fig. 4C) and the fully disordered C terminus of the static FADH^•^ state structure (Fig. 1D). Under time-resolved conditions, the kinetics of the competing unfolding and reordering result in mixed populations. In contrast, continuous illumination used to obtain the static FADH^•^ state structure drives the reaction completely toward the signaling state. We propose that observing completely the structural dynamics of α22 unfolding would require longer delays on the order of hundreds of milliseconds to seconds.

Unlike ultrafast, subpicosecond transitions along protein backbones (*44*, *45*), which originate directly from the chromophore as epicenter, the D321/Y373 PCET switch is 22 Å distant from FAD and causes much slower disordering of the α22 backbone in the microsecond-millisecond range. Such a delayed order-disorder transition may hence be relevant not only to this photoreceptor but also in proteins depending on PT steps or other sudden electrostatic changes for their function.

## MATERIALS AND METHODS

### Experimental design

To produce structural snapshots of a CRY photocycle, we recombinantly produced large quantities of *Cr*aCRYΔCTE (UniProt entry A8J8W0), which we then crystallized by adapting previously established conditions (fig. S15) (*14*). Direct exposure of crystalline material to the XFEL at the BL2 SACLA beamline (*46*) produced the oxidized ground state *Cr*aCRY static structure (FAD_ox_; table S1). Treatment with blue light and reducing agents before data collection resulted in structures of the two stable reduced states (FADH^•^, FADH^−^; table S1), which were confirmed via icOS (Fig. 1B). Time-resolved structural snapshots were produced by activating crystals with a 450-nm optical parametric oscillator (OPO) pump laser, followed after a defined delay by the XFEL probe (fig. S4).

After data processing to produce complete, high-resolution datasets (tables S1 and S2), DED maps (*47*) were generated to highlight structural changes (figs. S6 to S10), and activated fractions were deconvoluted via structure factor extrapolation (fig. S16) (*28*, *48*). Refinement against extrapolated structure factors and DED maps resulted in structural coordinates (tables S1 and S2 and fig. S17) whose quality was then evaluated by generating calculated DED maps (figs. S18 to S21). Structural changes were also correlated with transient absorption ultraviolet visible (UV/Vis) spectroscopic data, which were produced via time-resolved icOS (Fig. 2D and figs. S5 and S12) and with in-solution proton-deuterium exchange MS data (fig. S3). Last, all time-resolved structural snapshots were assembled into a 3D molecular movie (movie S1).

### Protein production and purification of oxidized *Cr*aCRY and its E384Q mutant

Protein expression and purification were carried out according to published methods (*14*). In brief, an *Escherichia coli* BL21(DE3) culture bearing a pET28a-based construct that codes for *Cr*aCRYΔCTE with its C-terminal extension (CTE, E496-E595) replaced by a histidine tag was grown in 2YT medium at 37°C until it reached an optical density of 0.4 to 0.6 at 600 nm. The temperature was then lowered to 18°C, and isopropyl-β-d-thiogalactopyranoside was added to a final concentration of 10 μM. After cell harvesting via centrifugation, cell pellets were lysed via an emulsiflex-C5 homogenizer (Avestin) in lysis buffer [50 mM sodium phosphate (pH 7.8), 100 mM NaCl, 20% glycerol, a tip of a spatula of lyophilized deoxyribonuclease I and lysozyme]. *Cr*aCRYΔCTE was then purified via Ni–nitrilotriacetic acid chromatography (Roche), followed by heparin affinity chromatography (HiTrap 5-ml Heparin HP, GE Healthcare), with the protein eluted in 50 mM sodium phosphate (pH 7.8), 250 mM NaCl, and 20% glycerol. A final size exclusion chromatography (Superdex 200, GE Healthcare) as polishing step ensured sample monodispersity and buffer exchange to protein buffer [20 mM tris (pH 8.5) and 200 mM NaCl].

Site-directed mutagenesis generated the *Cr*aCRY E384Q mutant by using the previous construct as a template and a forward primer with the sequence 5′-GTTTTTCAGGAACATCTGATTGATCAGGACCACTATCTG-3′ along with its reverse primer. Expression and purification of the E384Q mutant followed the procedure described above.

### SFX-adapted *Cr*aCRY crystallization

Crystallization of *Cr*aCRYΔCTE was adapted and upscaled for SFX data collection from the previously established condition (*14*). A sterile-filtered *Cr*aCRY solution (3 mg/ml, 25-ml total volume) was mixed in a 1:1 ratio with crystallization buffer [0.1 M 2-(*N*-morpholino)ethanesulfonic acid (pH 5.6), 35% (w/v) PEG 4000]. To increase crystallization yield, the mix was supplemented with a micro-seed aliquot in a 1:100 ratio. Micro-seeds were prepared with the PTFE Seed Bead Kit (Hampton Research) according to the standard protocol provided with the kit. The crystallization batch was then aliquoted and stored in 15-ml conical tubes (Falcon) overnight, at 4°C, and in the dark.

At the beginning of the experimental beamtime, crystals were preconcentrated 10-fold via centrifugation (Eppendorf, Minispin) and partial decanting of crystal-free supernatant. The resulting crystal slurry was then passed through a 50-μm sorting filter (pluriSelect), which was washed with supernatant. Filtered, diluted crystals were concentrated again via centrifugation, and stored as a 10X preconcentrate to be used during data collection.

### Injector preparation of *Cr*aCRY in its different redox states

Injector preparation was started by crushing 500 μl of crystal preconcentrate with a microtube homogenizer (Fisherbrand Pellet pestle cordless motor, Thermo Fisher Scientific). Here, two crushing runs of 40 s (totally 80 s) were separated by a 40-s incubation period on ice. Then, crushed crystals were checked for their sizes under a microscope (fig. S15).

The crushed crystal suspension was further concentrated by centrifugation until the crystals had been compacted completely into semisolid pellets. The supernatant was discarded and the remaining crystal slurry was mixed in a 1:9 ratio with a hydrophobic grease matrix as described before (*49*). The embedded material was transferred into a φ4-mm injector cartridge, which was loaded into a SACLA high-viscosity injector (*50*) and capped with a 75-μm nozzle. All sample preparation operations were performed under red light to prevent light contamination, and injectors were transported inside opaque containers to the SACLA experimental hutch for mounting and subsequent data acquisition.

To obtain *Cr*aCRY crystals in its semiquinoid FADH^•^ state, homogenized, preconcentrated crystals were illuminated for 30 min under aerobic conditions with white light (Leica KD300). The injector was prepared as described above, maintaining white light illumination at all times. To obtain fully reduced crystals, we followed a previously described protocol producing fully reduced class II CPD photolyase crystals by a combination of exposure to a reducing agent [dithiothreitol (DTT) 50 mM] and white light under anaerobic conditions (*26*).

We validated *Cr*aCRY’s redox states via in crystallo spectroscopy (Fig. 1B). UV/Vis in crystallo spectra of *Cr*aCRY were recorded before illumination and after illumination either with or without treatment with DTT at the icOS laboratory (*51*). To demonstrate the effects of illumination and redox state change on the crystal lattice, pre- and post-illumination *c*-axis histograms were produced from *Cr*aCRY diffraction patterns during XFEL data collection. For that, an injector was filled with oxidized *Cr*aCRY crystals, and ~10,000 images were collected. Then, the injector was opened and sample was illuminated for 10 min with a white light, followed by data collection (Fig. 1B).

### Data acquisition for short delay times (10 ns to 7 ms)

Experiments were carried out at the SACLA BL2 beamline inside the DAPHNIS chamber (*52*) following previously established procedures (*26*, *28*). Detection took place within a helium chamber via a short-working distance multi-port CCD detector (*46*, *53*) with a 50-mm nominal sample-to-detector distance. Before the experiment started and to prevent both background noise on the detector and premature reoxidation of radical species, special attention was paid to reach at least 98% levels of helium within the chamber (gas flow of 1.7 liter/min). Once microaerobic conditions were reached, the sample was extruded at a flow rate of 2.8 μl/min (for TR-SFX experiments) or 1 μl/min (for SFX experiment). Images were obtained using a 10-keV x-ray beam with 30-Hz pulse frequency, a pulse duration of <10 fs and beam diameter of 1.5 μm. The x-ray beam was focused on the extruded filament at between 200 and 350 μm below the injector nozzle. Initial spot finding and image sorting was automatically performed via the cheetah pipeline (*54*), followed by on-site semiautomatic data processing with *CrystFEL* (*55*, *56*). Initial datasets were calculated from there, with on-site structure solution and DED construction being used for informing experimental decisions during data collection.

To trigger light-induced conformational changes, crystals were excited by a 10 or 15 Hz, 50 μJ, 450 nm, OPO pump laser in top-hat configuration with a focal spot diameter of 100 μm and 3-ns pulse duration (0.21 GW/cm^2^ nominal power; see text S1 for further details). The pump laser was centered on the same position as the XFEL. Here, once light contamination was ruled out by an initial run of ~7500 light-triggered indexed images acquired at 10 Hz (fig. S4, A and C), the pump laser frequency was switched to 15 Hz to maximize data collection of the light-triggered states (fig. S4A).

### Data acquisition for long delay times (33 to 233 ms)

Sample and injector preparation and XFEL parameters were identical between the short and long delay setups. However, to observe conformational changes beyond the ~7-ms limit imposed by the short delay parameters (fig. S4A), we lowered the sample flow speed to 0.76 μl/min and slightly modified the pump laser setup as follows. The pump laser top hat focal spot was enlarged to a 150-μm diameter and offset from the XFEL focal spot by 70 μm, as well as switched to a 3-Hz pump laser frequency while maintaining the XFEL 30-Hz repetition rate (fig. S4B). To compensate for the larger focal spot, a pulse energy of 111 μJ was chosen, yielding a constant nominal power density of 0.21 GW/cm^2^. The resulting geometry ensured that four XFEL pulses would fall within the sample region illuminated by the pump laser, corresponding to one concurrent with pumping, and 33, 66, and 100 ms after pumping (fig. S4B). By further offsetting the pump laser from the XFEL by 263 μm, we were able to produce further delays between 133 and 233 ms (figs. S6 to S10).

### Off-site processing of SFX data

Initial indexing and processing parameters, including detector geometry and distance, were further optimized in CrystFEL (*56*) to maximize the number of indexed images. The resulting intensities were merged via process_hkl and finally converted to structure factors by TRUNCATE from the CCP4 suite (*57*). Light-triggered images were separately treated according to their delay times, while a large dark dataset was constructed from all nontriggered images which, after DED analysis (fig. S4C), lacked any light contamination. Last, to ensure good agreement between all datasets, all light-triggered datasets were scaled to the large dark dataset by SCALEIT from the CCP4 suite (*57*). Data processing statistics can be consulted in tables S1 to S3 including the analogous analysis of static datasets.

### Solution and refinement of static *Cr*aCRY structures in its three redox states

The dark, i.e., FAD_ox_ state, and FADH^•^ state datasets were solved via molecular replacement using the *Cr*aCRYΔCTE structure (6FN3) as search model for Phaser software (*58*). Automated restrained refinement with phenix.refine (*59*–*61*) as well as manual model building with Coot (*62*) followed. The FADH^−^ state dataset was solved by molecular substitution using 20 cycles of rigid body refinement in Refmac5 (*63*), using the refined FAD_ox_ state coordinates as initial model. Refinement statistics are shown in tables S1 to S2.

### Generating difference density maps

To visualize structural differences between dark data, i.e., the FAD_ox_ state, and either time-resolved snapshots or the fully reduced FADH^−^ state, isomorphous DED maps were generated with the corresponding phenix tool of the same name (*47*, *61*). Here, scaled datasets were used to generate difference amplitudes, with phase information extracted from dark coordinates. For data quality, only amplitudes with a signal/noise ratio of >3 were used. The high-resolution limit was set to the lowest one between two datasets being compared, while a low-resolution limit of 10 Å was used to prevent high-intensity, low-resolution reflections to dominate the overall DED maps (*48*). Notably, illumination of FAD_ox_ state crystals to produce the FADH^•^ state resulted in changes of the crystal lattice (Fig. 1D), which excluded calculation of isomorphous DED maps.

### Density integration from DED maps

To trace the time evolution of positive and negative DED signals in the time-resolved snapshots, density integration of DED map peaks was conducted as follows: First, DED maps containing only peaks above a 3σ level were produced with MAPMASK (*57*). The same program was then used to extract DED values around a specific structural feature by providing a coordinate file mask containing the atoms of interest. Last, all positive or negative DED values within the target region were summed up to produce integrated density.

### Structure factor extrapolation and structural refinement of partially occupied structures

Excitation by the pump laser in TR-SFX does not result in 100% occupancy of reaction intermediates or products (*26*, *28*, *64*). Deconvolution of illuminated, experimental datasets (F_o,l_) via structure factor extrapolation is hence necessary to produce extrapolated structure factors (F_ext_) against which accurate atomic models can be refined (*48*).

Dataset extrapolation occurred in two rounds by established procedures (*26*, *28*, *48*). During the first round, Bayesian-weighted F_ext_ and their corresponding σ values were calculated at different *N* values (*N* = 2 per occupancy) following a scalar approach. Next, integrated residual negative densities (Δρ_res_) at each calculated occupancy were extracted from the extrapolated density map around D321, where significant DED signals (above 3σ) had been detected for all time points (fig. S10). Last, Δρ_res_ was plotted against *N* values (fig. S16). Since at low *N* values, the contribution of |F_c,dark_| dominates Δρ_res_, while at high *N* values, Bayesian weighted different amplitudes, Nw(|F_o,l_|-|F_o,dark_|), prevail; the *N* parameter, which accurately describes the occupancy of the excited species, can be estimated as the inflection point between these two behaviors (*26*, *48*).

At this point, and starting from dark coordinates, initial refinement of the time-resolved structure was performed by both real- and reciprocal-space refinement using Coot (*62*) and phenix.refine (*59*, *60*). R_free_ ≤ 0.5 at the highest-resolution shell was used as resolution cutoff criterium. In parallel, the difference structure factor correlation coefficient refinement (dFoCC) technique (see section “dFoCC and refinement reaction coordinates” and table S4) was used for accurate refinement of the H309, D321, E384, N395, and the FAD isoalloxazine moiety atomic positions (table S4 and figs. S6 to S10). The initial light dataset was then used for vectorial dataset extrapolation, as described elsewhere (*26*, *48*), followed by one cycle of coot-based model building and one cycle of automated refinement via phenix.refine.

### dFoCC and refinement of reaction coordinates

As previously discussed (*26*, *29*, *48*, *64*, *65*), time-resolved structural features may not be accurately described by monomer libraries available in standard refinement suites. While libraries were defined for molecules in their resting state, time-resolved snapshots may represent high-energy states. Accordingly, standard monomer descriptions may bias the refined coordinates toward inaccurate representations of their geometry (*26*). Previously, we showed that difference map real space correlation coefficient refinement (dFoCC) produced reasonable models of the light-activated state based on the observed difference structure factors, and the refined resting state coordinates (*28*). Briefly, for dFoCC, the dark coordinates were used to calculate (a) calculated dark structure factors (F_c,dark_), (b) dark phases (α_dark_), and (c) a large library of possible light state coordinates, from which possible calculated light structure factors were generated (F_c,l_). For each possible light state, a calculated |F_c,l_|-|F_c,dark_| DED map (DED_c_) was generated and compared to the experimental |F_o,l_|-|F_o,dark_| DED map (DED_o_) via correlation analysis. The best set of coordinates, i.e., the one producing the highest correlation coefficient between DED_c_ and DED_o_, was then used to produce an updated library of possible coordinates, which were again evaluated via DED_c_ versus DED_o_ correlation analysis. This iterative process continued until the correlation coefficient converged, or the conformational space became unreasonably small for the resolution of the experimental data, i.e., 0.1 Å for interatomic distances and 0.1° for bond angles.

Clearly, dFoCC refinement depends on a reliable set of reaction coordinates that describes the conformational space of the target moiety to be refined well. For the FAD isoalloxazine moiety, we chose the same reaction coordinates that previously produced consistent results for transient oxidation of FADH^−^ during light-induced photolyase DNA repair (figs. S11 and S22) (*28*).

In addition, we subjected several key residues, H309, D321, E384, and N395, to dFoCC refinement. Here, we chose reaction coordinates as rotational axes along all side-chain single bonds (fig. S23). As the observed D321 DED_o_ features extended into its main-chain volume, we also considered rotational axes that slightly changed the D321 main chain, as well as those of neighboring residues (fig. S23).

To produce the light state model, and after dFoCC converged, the initial coordinates of the target moieties in dark were manually replaced by refined coordinates. From this point on, dFoCC-refined coordinates were fixed, while the rest of the modified dark structure was further subjected to real and reciprocal space refinement via phenix.refine (*60*) and manual model building with COOT (*62*). As an illustration of the results from dFoCC refinement, figs. S6 to S10 show DED_o_ maps while figs. S18 to S21 the equivalent DED_c_ maps. Further details on the reliability of the dFoCC method can be consulted in text S3.

### In crystallo optical spectroscopy

Static and transient in crystallo UV/Vis absorption spectra were recorded at the icOS laboratory of the ESRF (*51*). For pump-probe data collection at room temperature, crystals with dimensions of (20 to 40) μm by (100 to 200) μm by (10 to 20) μm were mounted under red-light conditions and probed at various delays (10 μs to 5 s) between the pulse of a nanosecond laser tuned at 450 nm and a 2-μs xenon flash lamp pulse using the TR-icOS instrument (*66*). Laser pulse energy was adjusted to three different light fluences at the sample position (0.0316, 0.0984, and 0.161 GW/cm^2^) to validate whether multiphoton absorption contributed to artefacts. Since the FADH^•^ state persists for hours after crystal illumination, only one measurement per crystal could be performed. Consequently, using a different crystal for each pair of dark-adapted and time-resolved spectra resulted in varying optical path lengths, precluding quantitative analysis through direct comparison of the time-resolved spectra.

For each crystal the dark UV/Vis spectrum was subtracted from the transient spectrum. Subsequently, for each of the three power levels, a matrix of pairwise Pearson correlation coefficients was computed between all resulting difference spectra and visualized as correlation maps (Fig. 2D for the 0.161 GW/cm^2^ power; fig. S5D for 0.0316, 0.0984, and 0.161 GW/cm^2^). To smooth the difference spectra before calculating pairwise correlation coefficients, a Savitzky-Golay filter (21-point window length, third-degree polynomial) was applied, minimizing the contribution of random measurement fluctuations. To ensure consistency across the correlation maps for all three power levels, each position in the map was assigned a uniform size corresponding to a unique time point. When multiple spectra were collected for the same time point, they were allocated equal portions of the designated space.

For quantitative analysis, the area between 580 and 640 nm in the difference spectrum that corresponded to the center of the FADH^•^ species-specific absorption band was integrated. To account for the difference in optical path length between measurements, this integrated absorbance value was normalized by the integrated absorption from 456 to 490 nm in the dark-adapted spectrum, which corresponds to the red side of the absorption band of the oxidized flavin (fig. S12A). The red side of the oxidized flavin absorption band was chosen to minimize the effect of Rayleigh scattering, which contributes considerably to measured absorbance at that wavelength (*67*). The remaining scattering contribution was subtracted from the dark-adapted spectrum before integration using a software developed for in crystallo absorption spectroscopy data correction (https://github.com/ncara/icos). To mitigate the strong noise floor of the in crystallo spectra (fig. S12), we chose to integrate characteristic spectral bands (*68*) (456 to 490 nm for FAD_ox_ and 580 to 640 nm for FADH^•^) instead of tracking changes at single wavelengths.

For the purpose of comparing different power levels, integrated absorption values were then standardized for a maximum amplitude of 1. A first-order kinetic equation was then fitted to the standardized integrated FADH^•^ absorption values, with repeated measurements averaged when possible. This fit, as well as the corresponding data points and the resulting time constants are represented in Fig. 2D for the 0.161 GW/cm^2^ power and fig. S5 for 0.0316, 0.0984, and 0.161 GW/cm^2^.

### Estimation of FADH^•^ accumulation upon photoreduction in solution at various pH values

Seventy microliters of dark-adapted protein diluted to 20 μM was applied to a Micro Bio-Spin 6 column (Bio-Rad) and equilibrated with reaction buffer (20 mM phosphate, 500 mM NaCl, and 10% glycerol, at pH 5.5, 6.0, 6.5, 7.0, 7.5, 8.0, or 8.5). Sixty microliters of the eluate was further diluted by adding 180-μl of the reaction buffer, and the mixture was transferred into a 10 mm–by–2 mm–by–8 mm (length by width by height) inner volume quartz cuvette (Starna, 16.160-F/4/Q/10 GL 14/2/Z15). Before and after illuminating the sample with white light (430 to 800 nm) from a MAX-150 xenon lamp (Asahi Spectra) through the 10 mm–by–8 mm window on ice, absorption spectra were recorded along the 10 mm path by a UV/Vis V-730 spectrometer (JASCO). Because we performed the experiment under aerobic conditions without any external reducing agents, no FADH^−^ was accumulated during the measurement. On the basis of absorption changes at 450 (mostly FAD_ox_) and 630 nm (FADH^•^), the concentration of FAD in each state was calculated by Lambert-Beer law using reported molar extinction coefficients for FAD_ox_ (450 nm: 11204 M^−1^ cm^−1^, 635 nm: 0 M^−1^ cm^−1^) and FADH^•^ (450 nm: 3833 M^−1^ cm^−1^, 635 nm: 4958 M^−1^ cm^−1^) (*68*).

### SVD analysis

For the analysis of the C-terminal dynamics by SVD, each of the 19 DED maps was masked by the atoms of all residues encompassing the C-terminal helix. Briefly, the series of restricted isomorphous DED maps is decomposed into three objects. The first is a set of time-invariant structural elements (or left singular vectors, lSV) in the form of a set of DED maps. The second object contains a set of magnitudes over time (or right singular vectors, rSV) for each time-invariant lSV. The last object contains a set of weights (or singular values, SV) for each time-invariant lSV, indicating how meaningful it is in the overall description of the events happening over the original series of restricted DED maps. For each time point, the original DED map can be recomposed by a linear combination of the time-invariant lSV, scaled by their magnitude (from the rSV) for that particular time point, and their overall weight (their SV). The analysis was performed using in-house python scripts available at https://github.com/ncara/SVD, as described in detail in Maestre-Reyna *et al.* (*28*) and originally developed by Schmidt *et al.* (*69*).

### Structural visualization of SVD data

To better visualize the time-independent lSVs, they were treated as DED maps, converted to difference structure factors via CCP4 (*57*) and extrapolated as described under section “Structure factor extrapolation and structural refinement of partially occupied structures,” according to previously published procedures (*70*). Structural models were prepared by one cycle of phenix.refine (*60*) automated refinement and COOT model building (*62*) against the lSV extrapolated structure factors (Fig. 4, F and G). Although the degree to which such structural models are bias free is debatable, they are well suited as visualization tools to simplify the complex interpretation of lSV maps. Accordingly, instead of uploading the coordinates to the Protein Data Bank, the lSV0 and lSV1 models are provided with the current manuscript as files S1 and S2, respectively.

### HDX-MS experiments

The hydrogen-deuterium exchange MS (HDX-MS) experiments were performed to corroborate the light-dependent conformational changes observed via TR-SFX. *Cr*aCRY samples were subjected to HDX-MS experiments either in the dark or after 15-min illumination with a 455 nm, 5.9 mW cm^−2^ (224 μmol m^−2^ s^−1^) LED. *Cr*aCRY protein solutions were diluted 10-fold with D_2_O-containing buffer [50 mM NaH_2_PO_4_/Na_2_HPO_4_ and 100 mM NaCl (pH 7.8)] and incubated for 10, 100, or 1000 s at 25°C. The HDX reaction was stopped by addition cold quench buffer [400 mM KH_2_PO_4_/H_3_PO_4_ and 2 M guanidine-HCl (pH 2.2), 1°C] in a 1:1 ratio. Samples were then immediately injected into an ACQUITY UPLC M-class system with HDX technology (Waters) (*71*). After digestion with immobilized porcine pepsin at 12°C in water +0.1% (v/v) formic acid, the resulting *Cr*aCRY peptides were trapped on a C18 column. Peptides were then separated via a gradient of water +0.1% (v/v) formic acid (eluent A) and acetonitrile +0.1% (v/v) formic acid (eluent B). Mass spectra were recorded on a G2-Si HDMS mass spectrometer (Waters) in high-definition MS (HDMS) positive ion mode. [Glu1]-fibrinopeptide B (Waters) was used for lock mass correction. Undeuterated samples of *Cr*aCRY were prepared similarly using undeuterated buffer [50 mM NaH_2_PO_4_/Na_2_HPO_4_ and 100 mM NaCl (pH 7.8)], and mass spectra acquired in Enhanced HDMS positive ion mode (*72*, *73*). All measurements were performed in triplicate. *Cr*aCRY peptide identification and assignment of deuterium incorporation were conducted with the PLGS and DynamX 3.0 software (Waters), respectively, as described elsewhere (*74*).

### Data visualization

All structural figures in the current work were prepared with PyMOL (*75*), while graphs with Graphpad Prism and the matplotlib python package.

## Supplementary Materials

### The PDF file includes:

Supplementary Text S1 to S3 Figs. S1 to S23 Tables S1 to S4 Legends for Supplementary Coordinates Files S1 and S2 Legend for movie S1 References

## References

[1] Q. Mei, V. Dvornyk, Evolutionary history of the photolyase/cryptochrome superfamily in eukaryotes. PLOS ONE 10, e0135940 (2015). 26352435 10.1371/journal.pone.0135940 PMC4564169

[2] I. H. Kavakli, I. Baris, M. Tardu, Ş. Gül, H. Öner, S. Çal, S. Bulut, D. Yarparvar, Ç. Berkel, P. Ustaoğlu, C. Aydın, The photolyase/cryptochrome family of proteins as DNA repair enzymes and transcriptional repressors. Photochem. Photobiol. 93, 93–103 (2017). 28067410 10.1111/php.12669

[3] I. Chaves, R. Pokorny, M. Byrdin, N. Hoang, T. Ritz, K. Brettel, L.-O. Essen, G. T. J. van der Horst, A. Batschauer, M. Ahmad, The cryptochromes: Blue light photoreceptors in plants and animals. Annu. Rev. Plant Biol. 62, 335–364 (2011). 21526969 10.1146/annurev-arplant-042110-103759

[4] K. Brettel, M. Byrdin, Reaction mechanisms of DNA photolyase. Curr. Opin. Struct. Biol. 20, 693–701 (2010). 20705454 10.1016/j.sbi.2010.07.003

[5] A. Sancar, Mechanisms of DNA repair by photolyase and excision nuclease (Nobel lecture). Angew. Chem. Int. Ed. Engl. 55, 8502–8527 (2016). 27337655 10.1002/anie.201601524

[6] L. O. Essen, T. Klar, Light-driven DNA repair by photolyases. Cell. Mol. Life Sci. 63, 1266–1277 (2006). 16699813 10.1007/s00018-005-5447-y PMC11136382

[7] T. Ritz, P. Thalau, J. B. Phillips, R. Wiltschko, W. Wiltschko, Resonance effects indicate a radical-pair mechanism for avian magnetic compass. Nature 429, 177–180 (2004). 15141211 10.1038/nature02534

[8] P. J. Hore, H. Mouritsen, The radical-pair mechanism of magnetoreception. Annu. Rev. Biophys. 45, 299–344 (2016). 27216936 10.1146/annurev-biophys-032116-094545

[9] J. Xu, L. E. Jarocha, T. Zollitsch, M. Konowalczyk, K. B. Henbest, S. Richert, M. J. Golesworthy, J. Schmidt, V. Déjean, D. J. C. Sowood, M. Bassetto, J. Luo, J. R. Walton, J. Fleming, Y. Wei, T. L. Pitcher, G. Moise, M. Herrmann, H. Yin, H. Wu, R. Bartölke, S. J. Käsehagen, S. Horst, G. Dautaj, P. D. F. Murton, A. S. Gehrckens, Y. Chelliah, J. S. Takahashi, K.-W. Koch, S. Weber, I. A. Solov’yov, C. Xie, S. R. Mackenzie, C. R. Timmel, H. Mouritsen, P. J. Hore, Magnetic sensitivity of cryptochrome 4 from a migratory songbird. Nature 594, 535–540 (2021). 34163056 10.1038/s41586-021-03618-9

[10] K. Schulten, C. E. Swenberg, A. Weiler, A biomagnetic sensory mechanism based on magnetic field modulated coherent electron spin motion. Z. Phys. Chem. 111, 1–5 (1978).

[11] B. Beel, K. Prager, M. Spexard, S. Sasso, D. Weiss, N. Müller, M. Heinnickel, D. Dewez, D. Ikoma, A. R. Grossman, T. Kottke, M. Mittag, A flavin binding cryptochrome photoreceptor responds to both blue and red light in Chlamydomonas reinhardtii. Plant Cell 24, 2992–3008 (2012). 22773746 10.1105/tpc.112.098947 PMC3426128

[12] J. Petersen, A. Rredhi, J. Szyttenholm, S. Oldemeyer, T. Kottke, M. Mittag, The world of algae reveals a broad variety of cryptochrome properties and functions. Front. Plant Sci. 12, 766509 (2021). 34790217 10.3389/fpls.2021.766509 PMC8591175

[13] Y. Zou, S. Wenzel, N. Müller, K. Prager, E.-M. Jung, E. Kothe, T. Kottke, M. Mittag, An animal-like cryptochrome controls the *Chlamydomonas* sexual cycle. Plant Physiol. 174, 1334–1347 (2017). 28468769 10.1104/pp.17.00493 PMC5490917

[14] S. Franz, E. Ignatz, S. Wenzel, H. Zielosko, E. P. G. N. Putu, M. Maestre-Reyna, M.-D. Tsai, J. Yamamoto, M. Mittag, L.-O. Essen, Structure of the bifunctional cryptochrome aCRY from Chlamydomonas reinhardtii. Nucleic Acids Res. 46, 8010–8022 (2018). 30032195 10.1093/nar/gky621 PMC6125616

[15] D. Nohr, S. Franz, R. Rodriguez, B. Paulus, L. O. Essen, S. Weber, E. Schleicher, Extended electron-transfer in animal cryptochromes mediated by a tetrad of aromatic amino acids. Biophys. J. 111, 301–311 (2016). 27463133 10.1016/j.bpj.2016.06.009 PMC4968396

[16] S. Oldemeyer, S. Franz, S. Wenzel, L. O. Essen, M. Mittag, T. Kottke, Essential role of an unusually long-lived tyrosyl radical in the response to red light of the animal-like cryptochrome acry. J. Biol. Chem. 291, 14062–14071 (2016). 27189948 10.1074/jbc.M116.726976 PMC4933166

[17] R. Martin, F. Lacombat, A. Espagne, N. Dozova, P. Plaza, J. Yamamoto, P. Müller, K. Brettel, A. De La Lande, Ultrafast flavin photoreduction in an oxidized animal (6-4) photolyase through an unconventional tryptophan tetrad. Phys. Chem. Chem. Phys. 19, 24493–24504 (2017). 28890968 10.1039/c7cp04555g

[18] D. Timmer, A. Frederiksen, D. C. Lünemann, A. R. Thomas, J. Xu, R. Bartölke, J. Schmidt, T. Kubař, A. De Sio, I. A. Solov’yov, H. Mouritsen, C. Lienau, Tracking the electron transfer cascade in European robin Cryptochrome 4 mutants. J. Am. Chem. Soc. 145, 11566–11578 (2023). 37195086 10.1021/jacs.3c00442 PMC10236492

[19] A. K. Michael, J. L. Fribourgh, R. N. Van Gelder, C. L. Partch, Animal cryptochromes: Divergent roles in light perception, circadian timekeeping and beyond. Photochem. Photobiol. 93, 128–140 (2017). 27891621 10.1111/php.12677 PMC5397253

[20] R. Zangl, S. Soravia, M. Saft, J. G. Löffler, J. Schulte, C. J. Rosner, J. Bredenbeck, L. O. Essen, N. Morgner, Time-resolved ion mobility mass spectrometry to solve conformational changes in a cryptochrome. J. Am. Chem. Soc. 146, 14468–14478 (2024). 38757172 10.1021/jacs.3c13818

[21] F. Lacombat, A. Espagne, N. Dozova, P. Plaza, P. Müller, K. Brettel, S. Franz-Badur, L. O. Essen, Ultrafast oxidation of a Tyrosine by proton-coupled electron transfer promotes light activation of an animal-like cryptochrome. J. Am. Chem. Soc. 141, 13394–13409 (2019). 31368699 10.1021/jacs.9b03680

[22] C. Kupitz, S. Basu, I. Grotjohann, R. Fromme, N. A. Zatsepin, K. N. Rendek, M. S. Hunter, R. L. Shoeman, T. A. White, D. Wang, D. James, J. H. Yang, D. E. Cobb, B. Reeder, R. G. Sierra, H. Liu, A. Barty, A. L. Aquila, D. Deponte, R. A. Kirian, S. Bari, J. J. Bergkamp, K. R. Beyerlein, M. J. Bogan, C. Caleman, T. C. Chao, C. E. Conrad, K. M. Davis, H. Fleckenstein, L. Galli, S. P. Hau-Riege, S. Kassemeyer, H. Laksmono, M. Liang, L. Lomb, S. Marchesini, A. V. Martin, M. Messerschmidt, D. Milathianaki, K. Nass, A. Ros, S. Roy-Chowdhury, K. Schmidt, M. Seibert, J. Steinbrener, F. Stellato, L. Yan, C. Yoon, T. A. Moore, A. L. Moore, Y. Pushkar, G. J. Williams, S. Boutet, R. B. Doak, U. Weierstall, M. Frank, H. N. Chapman, J. C. H. Spence, P. Fromme, Serial time-resolved crystallography of photosystem II using a femtosecond x-ray laser. Nature 513, 261–265 (2014). 25043005 10.1038/nature13453 PMC4821544

[23] J. C. H. Spence, XFELs for structure and dynamics in biology. IUCrJ 4, 322–339 (2017). 10.1107/S2052252517005760 PMC5571796 28875020

[24] P. Fromme, XFELs open a new era in structural chemical biology. Nat. Chem. Biol. 11, 895–899 (2015). 26575227 10.1038/nchembio.1968 PMC4839532

[25] A. M. Orville, Recent results in time resolved serial femtosecond crystallography at XFELs. Curr. Opin. Struct. Biol. 65, 193–208 (2020). 33049498 10.1016/j.sbi.2020.08.011

[26] M. Maestre-Reyna, C. H. Yang, E. Nango, W. C. Huang, E. P. G. Ngurah Putu, W. J. Wu, P. H. Wang, S. Franz-Badur, M. Saft, H. J. Emmerich, H. Y. Wu, C. C. Lee, K. F. Huang, Y. K. Chang, J. H. Liao, J. H. Weng, W. Gad, C. W. Chang, A. H. Pang, M. Sugahara, S. Owada, Y. Hosokawa, Y. Joti, A. Yamashita, R. Tanaka, T. Tanaka, F. Luo, K. Tono, K. C. Hsu, S. Kiontke, I. Schapiro, R. Spadaccini, A. Royant, J. Yamamoto, S. Iwata, L. O. Essen, Y. Bessho, M. D. Tsai, Serial crystallography captures dynamic control of sequential electron and proton transfer events in a flavoenzyme. Nat. Chem. 14, 677–685 (2022). 35393554 10.1038/s41557-022-00922-3

[27] S. Franz-Badur, A. Penner, S. Straß, S. von Horsten, U. Linne, L. O. Essen, Structural changes within the bifunctional cryptochrome/photolyase *Cra*CRY upon blue light excitation. Sci. Rep. 9, 9896 (2019). 31289290 10.1038/s41598-019-45885-7 PMC6616342

[28] M. Maestre-Reyna, P.-H. Wang, E. Nango, Y. Hosokawa, M. Saft, A. Furrer, C.-H. Yang, E. P. G. N. Putu, W.-J. Wu, H.-J. Emmerich, N. Caramello, S. Franz-Badur, C. Yang, S. Engilberge, M. Wranik, H. L. Glover, T. Weinert, H.-Y. Wu, C.-C. Lee, W.-C. Huang, K.-F. Huang, Y.-K. Chang, J.-H. Liao, J.-H. Weng, W. Gad, C.-W. Chang, A. H. Pang, K.-C. Yang, W.-T. Lin, Y.-C. Chang, D. Gashi, E. Beale, D. Ozerov, K. Nass, G. Knopp, P. J. M. Johnson, C. Cirelli, C. Milne, C. Bacellar, M. Sugahara, S. Owada, Y. Joti, A. Yamashita, R. Tanaka, T. Tanaka, F. Luo, K. Tono, W. Zarzycka, P. Müller, M. A. Alahmad, F. Bezold, V. Fuchs, P. Gnau, S. Kiontke, L. Korf, V. Reithofer, C. J. Rosner, E. M. Seiler, M. Watad, L. Werel, R. Spadaccini, J. Yamamoto, S. Iwata, D. Zhong, J. Standfuss, A. Royant, Y. Bessho, L.-O. Essen, M.-D. Tsai, Visualizing the DNA repair process by a photolyase at atomic resolution. Science 382, eadd7795 (2023). 38033054 10.1126/science.add7795

[29] A. Cellini, M. K. Shankar, A. Nimmrich, L. A. Hunt, L. Monrroy, J. Mutisya, A. Furrer, E. V. Beale, M. Carrillo, T. N. Malla, P. Maj, L. Vrhovac, F. Dworkowski, C. Cirelli, P. J. M. Johnson, D. Ozerov, E. A. Stojković, L. Hammarström, C. Bacellar, J. Standfuss, M. Maj, M. Schmidt, T. Weinert, J. A. Ihalainen, W. Y. Wahlgren, S. Westenhoff, Directed ultrafast conformational changes accompany electron transfer in a photolyase as resolved by serial crystallography. Nat. Chem. 16, 624–632 (2024). 38225270 10.1038/s41557-023-01413-9 PMC10997514

[30] I. M. M. Wijaya, T. Domratcheva, T. Iwata, E. D. Getzoff, H. Kandori, Single hydrogen bond donation from Flavin N5 to proximal asparagine ensures FAD reduction in DNA photolyase. J. Am. Chem. Soc. 138, 4368–4376 (2016). 27002596 10.1021/jacs.5b10533

[31] T. Iwata, Y. Zhang, K. Hitomi, E. D. Getzoff, H. Kandori, Key dynamics of conserved asparagine in a cryptochrome/photolyase family protein by Fourier transform infrared spectroscopy. Biochemistry 49, 8882–8891 (2010). 20828134 10.1021/bi1009979 PMC4329311

[32] S. M. Harper, L. C. Neil, K. H. Gardner, Structural basis of a phototropin light switch. Science 301, 1541–1544 (2003). 12970567 10.1126/science.1086810

[33] P. Li, H. Cheng, V. Kumar, C. S. Lupala, X. Li, Y. Shi, C. Ma, K. Joo, J. Lee, H. Liu, Y.-W. Tan, Direct experimental observation of blue-light-induced conformational change and intermolecular interactions of cryptochrome. Commun. Biol. 5, 1103 (2022). 36257983 10.1038/s42003-022-04054-9 PMC9579160

[34] J. J. Goings, P. Li, Q. Zhu, S. Hammes-Schiffer, Formation of an unusual glutamine tautomer in a blue light using flavin photocycle characterizes the light-adapted state. Proc. Natl. Acad. Sci. U.S.A. 117, 26626–26632 (2020). 33037153 10.1073/pnas.2016719117 PMC7604415

[35] T. Fujisawa, S. Masuda, Light-induced chromophore and protein responses and mechanical signal transduction of BLUF proteins. Biophys. Rev. 10, 327–337 (2018). 29235080 10.1007/s12551-017-0355-6 PMC5899715

[36] N.-E. Christou, V. Apostolopoulou, D. V. M. Melo, M. Ruppert, A. Fadini, A. Henkel, J. Sprenger, D. Oberthuer, S. Günther, A. Pateras, A. R. Mashhour, O. M. Yefanov, M. Galchenkova, P. Y. A. Reinke, V. Kremling, T. E. S. Scheer, E. R. Lange, P. Middendorf, R. Schubert, E. De Zitter, K. Lumbao-Conradson, J. Herrmann, S. Rahighi, A. Kunavar, E. V. Beale, J. H. Beale, C. Cirelli, P. J. M. Johnson, F. Dworkowski, D. Ozerov, Q. Bertrand, M. Wranik, C. Bacellar, S. Bajt, S. Wakatsuki, J. A. Sellberg, N. Huse, D. Turk, H. N. Chapman, T. J. Lane, Time-resolved crystallography captures light-driven DNA repair. Science 382, 1015–1020 (2023). 38033070 10.1126/science.adj4270

[37] T. Kottke, A. Batschauer, M. Ahmad, J. Heberle, Blue-light-induced changes in arabidopsis cryptochrome 1 probed by FTIR difference spectroscopy. Biochemistry 45, 2472–2479 (2006). 16489739 10.1021/bi051964b

[38] Y. Geisselbrecht, S. Frühwirth, C. Schroeder, A. J. J. Pierik, G. Klug, L.-O. Essen, CryB from Rhodobacter sphaeroides: A unique class of cryptochromes with new cofactors. EMBO Rep. 13, 233–239 (2012). 10.1038/embor.2012.2 PMC3323124 22290493

[39] B. D. Zoltowski, A. T. Vaidya, D. Top, J. Widom, M. W. Young, B. R. Crane, Structure of full-length Drosophila cryptochrome. Nature 480, 396–399 (2011). 22080955 10.1038/nature10618 PMC3240699

[40] S. N. Nangle, C. Rosensweig, N. Koike, H. Tei, J. S. Takahashi, C. B. Green, N. Zheng, Molecular assembly of the period-cryptochrome circadian transcriptional repressor complex. eLife 3, e03674 (2014). 25127877 10.7554/eLife.03674 PMC4157330

[41] I. Schmalen, S. Reischl, T. Wallach, R. Klemz, A. Grudziecki, J. R. Prabu, C. Benda, A. Kramer, E. Wolf, Interaction of circadian clock proteins CRY1 and PER2 is modulated by zinc binding and disulfide bond formation. Cell 157, 1203–1215 (2014). 24855952 10.1016/j.cell.2014.03.057

[42] W. Xing, L. Busino, T. R. Hinds, S. T. Marionni, N. H. Saifee, M. F. Bush, M. Pagano, N. Zheng, SCF FBXL3 ubiquitin ligase targets cryptochromes at their cofactor pocket. Nature 496, 64–68 (2013). 23503662 10.1038/nature11964 PMC3618506

[43] T. Ritz, S. Adem, K. Schulten, A model for photoreceptor-based magnetoreception in birds. Biophys. J. 78, 707–718 (2000). 10653784 10.1016/S0006-3495(00)76629-X PMC1300674

[44] T. R. M. Barends, L. Foucar, A. Ardevol, K. Nass, A. Aquila, S. Botha, R. B. Doak, K. Falahati, E. Hartmann, M. Hilpert, M. Heinz, M. C. Hoffmann, J. Köfinger, J. E. Koglin, G. Kovacsova, M. Liang, D. Milathianaki, H. T. Lemke, J. Reinstein, C. M. Roome, R. L. Shoeman, G. J. Williams, I. Burghardt, G. Hummer, S. Boutet, I. Schlichting, Direct observation of ultrafast collective motions in CO myoglobin upon ligand dissociation. Science 350, 445–450 (2015). 26359336 10.1126/science.aac5492

[45] P. Nogly, T. Weinert, D. James, S. Carbajo, D. Ozerov, A. Furrer, D. Gashi, V. Borin, P. Skopintsev, K. Jaeger, K. Nass, P. Båth, R. Bosman, J. Koglin, M. Seaberg, T. Lane, D. Kekilli, S. Brünle, T. Tanaka, W. Wu, C. Milne, T. White, A. Barty, U. Weierstall, V. Panneels, E. Nango, S. Iwata, M. Hunter, I. Schapiro, G. Schertler, R. Neutze, J. Standfuss, Retinal isomerization in bacteriorhodopsin captured by a femtosecond x-ray laser. Science 361, eaat0094 (2018). 29903883 10.1126/science.aat0094

[46] M. Yabashi, H. Tanaka, T. Ishikawa, Overview of the SACLA facility. J. Synchrotron Radiat. 22, 477–484 (2015). 25931056 10.1107/S1600577515004658 PMC4416664

[47] M. A. Rould, C. W. Carter Jr., Isomorphous difference methods. Methods Enzymol. 374, 145–163 (2003). 14696372 10.1016/S0076-6879(03)74007-5

[48] M. Schmidt, Time-resolved macromolecular crystallography at pulsed x-ray sources. Int. J. Mol. Sci. 20, 1401 (2019). 30897736 10.3390/ijms20061401 PMC6470897

[49] M. Sugahara, E. Mizohata, E. Nango, M. Suzuki, T. Tanaka, T. Masuda, R. Tanaka, T. Shimamura, Y. Tanaka, C. Suno, K. Ihara, D. Pan, K. Kakinouchi, S. Sugiyama, M. Murata, T. Inoue, K. Tono, C. Song, J. Park, T. Kameshima, T. Hatsui, Y. Joti, M. Yabashi, S. Iwata, Grease matrix as a versatile carrier of proteins for serial crystallography. Nat. Methods 12, 61–63 (2014). 25384243 10.1038/nmeth.3172

[50] Y. Shimazu, K. Tono, T. Tanaka, Y. Yamanaka, T. Nakane, C. Mori, K. T. Kimura, T. Fujiwara, M. Sugahara, R. Tanaka, R. B. Doak, T. Shimamura, S. Iwata, E. Nango, M. Yabashi, High-viscosity sample-injection device for serial femtosecond crystallography at atmospheric pressure. J. Appl. Cryst. 52, 1280–1288 (2019). 31798359 10.1107/S1600576719012846 PMC6878880

[51] D. von Stetten, T. Giraud, P. Carpentier, F. Sever, M. Terrien, F. Dobias, D. H. Juers, D. Flot, C. Mueller-Dieckmann, G. A. Leonard, D. De Sanctis, A. Royant, In crystallo optical spectroscopy (*ic*OS) as a complementary tool on the macromolecular crystallography beamlines of the ESRF. Acta Crystallogr. D Biol. Crystallogr. 71, 15–26 (2015). 25615856 10.1107/S139900471401517X PMC4304682

[52] K. Tono, E. Nango, M. Sugahara, C. Song, J. Park, T. Tanaka, R. Tanaka, Y. Joti, T. Kameshima, S. Ono, T. Hatsui, E. Mizohata, M. Suzuki, T. Shimamura, Y. Tanaka, S. Iwata, M. Yabashi, Diverse application platform for hard x-ray diffraction in SACLA (DAPHNIS): Application to serial protein crystallography using an x-ray free-electron laser. J. Synchrotron Radiat. 22, 532–537 (2015). 25931065 10.1107/S1600577515004464 PMC4817517

[53] T. Kameshima, S. Ono, T. Kudo, K. Ozaki, Y. Kirihara, K. Kobayashi, Y. Inubushi, M. Yabashi, T. Horigome, A. Holland, K. Holland, D. Burt, H. Murao, T. Hatsui, Development of an x-ray pixel detector with multi-port charge-coupled device for x-ray free-electron laser experiments. Rev. Sci. Instrum. 85, 033110 (2014). 24689567 10.1063/1.4867668

[54] T. Nakane, Y. Joti, K. Tono, M. Yabashi, E. Nango, S. Iwata, R. Ishitani, O. Nureki, Data processing pipeline for serial femtosecond crystallography at SACLA. J. Appl. Cryst. 49, 1035–1041 (2016). 27275146 10.1107/S1600576716005720 PMC4886989

[55] T. A. White, Processing serial crystallography data with crystFEL: A step-by-step guide. Acta Crystallogr. D Struct. Biol. 75, 219–233 (2019). 30821710 10.1107/S205979831801238X PMC6400257

[56] T. A. White, R. A. Kirian, A. V. Martin, A. Aquila, K. Nass, A. Barty, H. N. Chapman, *CrystFEL*: A software suite for snapshot serial crystallography. J. Appl. Cryst. 45, 335–341 (2012).

[57] M. D. Winn, C. C. Ballard, K. D. Cowtan, E. J. Dodson, P. Emsley, P. R. Evans, R. M. Keegan, E. B. Krissinel, A. G. W. Leslie, A. McCoy, S. J. McNicholas, G. N. Murshudov, N. S. Pannu, E. A. Potterton, H. R. Powell, R. J. Read, A. Vagin, K. S. Wilson, Overview of the CCP4 suite and current developments. Acta Crystallogr. D Biol. Crystallogr. 67, 235–242 (2011). 21460441 10.1107/S0907444910045749 PMC3069738

[58] A. J. McCoy, R. W. Grosse-Kunstleve, P. D. Adams, M. D. Winn, L. C. Storoni, R. J. Read, Phaser crystallographic software. J. Appl. Cryst. 40, 658–674 (2007). 19461840 10.1107/S0021889807021206 PMC2483472

[59] D. Liebschner, P. V. Afonine, M. L. Baker, G. Bunkoczi, V. B. Chen, T. I. Croll, B. Hintze, L. W. Hung, S. Jain, A. J. McCoy, N. W. Moriarty, R. D. Oeffner, B. K. Poon, M. G. Prisant, R. J. Read, J. S. Richardson, D. C. Richardson, M. D. Sammito, O. V. Sobolev, D. H. Stockwell, T. C. Terwilliger, A. G. Urzhumtsev, L. L. Videau, C. J. Williams, P. D. Adams, Macromolecular structure determination using X-rays, neutrons and electrons: Recent developments in Phenix. Acta Crystallogr. D Struct. Biol. 75, 861–877 (2019). 31588918 10.1107/S2059798319011471 PMC6778852

[60] P. V. Afonine, R. W. Grosse-Kunstleve, N. Echols, J. J. Headd, N. W. Moriarty, M. Mustyakimov, T. C. Terwilliger, A. Urzhumtsev, P. H. Zwart, P. D. Adams, Towards automated crystallographic structure refinement with phenix.refine. Acta Crystallogr. D Biol. Crystallogr. 68, 352–367 (2012). 22505256 10.1107/S0907444912001308 PMC3322595

[61] P. D. Adams, P. V. Afonine, G. Bunkóczi, V. B. Chen, I. W. Davis, N. Echols, J. J. Headd, L. W. Hung, G. J. Kapral, R. W. Grosse-Kunstleve, A. J. McCoy, N. W. Moriarty, R. Oeffner, R. J. Read, D. C. Richardson, J. S. Richardson, T. C. Terwilliger, P. H. Zwart, PHENIX: A comprehensive Python-based system for macromolecular structure solution. Acta Crystallogr. D Biol. Crystallogr. 66, 213–221 (2010). 20124702 10.1107/S0907444909052925 PMC2815670

[62] P. Emsley, B. Lohkamp, W. G. Scott, K. Cowtan, Features and development of Coot. Acta Crystallogr. D Biol. Crystallogr. 66, 486–501 (2010). 20383002 10.1107/S0907444910007493 PMC2852313

[63] G. N. Murshudov, P. Skubák, A. A. Lebedev, N. S. Pannu, R. A. Steiner, R. A. Nicholls, M. D. Winn, F. Long, A. A. Vagin, REFMAC5 for the refinement of macromolecular crystal structures. Acta Crystallogr. D Biol. Crystallogr. 67, 355–367 (2011). 21460454 10.1107/S0907444911001314 PMC3069751

[64] M. Carrillo, S. Pandey, J. Sanchez, M. Noda, I. Poudyal, L. Aldama, T. N. Malla, E. Claesson, W. Y. Wahlgren, D. Feliz, V. Šrajer, M. Maj, L. Castillon, S. Iwata, E. Nango, R. Tanaka, T. Tanaka, L. Fangjia, K. Tono, S. Owada, S. Westenhoff, E. A. Stojković, M. Schmidt, High-resolution crystal structures of transient intermediates in the phytochrome photocycle. Structure 29, 743–754.e4 (2021). 33756101 10.1016/j.str.2021.03.004 PMC8405169

[65] U. K. Genick, G. E. O. Borgstahl, K. Ng, Z. Ren, C. Pradervand, P. M. Burke, V. Šrajer, T.-Y. Teng, W. Schildkamp, D. E. McRee, K. Moffat, E. D. Getzoff, Structure of a protein photocycle intermediate by millisecond time-resolved crystallography. Science 275, 1471–1475 (1997). 9045611 10.1126/science.275.5305.1471

[66] S. Engilberge, N. Caramello, S. Bukhdruker, M. Byrdin, T. Giraud, P. Jacquet, D. Scortani, R. Biv, H. Gonzalez, A. Broquet, P. Van Der Linden, S. L. Rose, D. Flot, T. Balandin, V. Gordeliy, J. M. Lahey-Rudolph, M. Roessle, D. De Sanctis, G. A. Leonard, C. Mueller-Dieckmann, A. Royant, The TR-icOS setup at the ESRF: Time-resolved microsecond UV-Vis absorption spectroscopy on protein crystals. Acta Crystallogr. D Struct. Biol. 80, 16–25 (2024). 38088897 10.1107/S2059798323010483 PMC10833346

[67] F. S. N. Dworkowski, M. A. Hough, G. Pompidor, M. R. Fuchs, Challenges and solutions for the analysis of in situ, in crystallo micro-spectrophotometric data. Acta Crystallogr. D Biol. Crystallogr. 71, 27–35 (2015). 25615857 10.1107/S1399004714015107 PMC4304683

[68] P. Müller, J. Yamamoto, R. Martin, S. Iwai, K. Brettel, Discovery and functional analysis of a 4th electron-transferring tryptophan conserved exclusively in animal cryptochromes and (6-4) photolyases. Chem. Commun. 51, 15502–15505 (2015). 10.1039/c5cc06276d 26355419

[69] M. Schmidt, S. Rajagopal, Z. Ren, K. Moffat, Application of singular value decomposition to the analysis of time-resolved macromolecular x-ray data. Biophys. J. 84, 2112–2129 (2003). 12609912 10.1016/S0006-3495(03)75018-8 PMC1302779

[70] M. Schmidt, R. Pahl, V. Srajer, S. Anderson, Z. Ren, H. Ihee, S. Rajagopal, K. Moffat, Protein kinetics: Structures of intermediates and reaction mechanism from time-resolved x-ray data. Proc. Natl. Acad. Sci. U.S.A. 101, 4799–4804 (2004). 15041745 10.1073/pnas.0305983101 PMC387328

[71] T. E. Wales, K. E. Fadgen, G. C. Gerhardt, J. R. Engen, High-speed and high-resolution UPLC separation at zero degrees celsius. Anal. Chem. 80, 6815–6820 (2008). 18672890 10.1021/ac8008862 PMC2562353

[72] S. J. Geromanos, J. P. C. Vissers, J. C. Silva, C. A. Dorschel, G. Z. Li, M. V. Gorenstein, R. H. Bateman, J. I. Langridge, The detection, correlation, and comparison of peptide precursor and product ions from data independent LC-MS with data dependant LC-MS/MS. Proteomics 9, 1683–1695 (2009). 19294628 10.1002/pmic.200800562

[73] G.-Z. Li, J. P. C. Vissers, J. C. Silva, D. Golick, M. V. Gorenstein, S. J. Geromanos, Database searching and accounting of multiplexed precursor and product ion spectra from the data independent analysis of simple and complex peptide mixtures. Proteomics 9, 1696–1719 (2009). 19294629 10.1002/pmic.200800564

[74] M. Osorio-Valeriano, F. Altegoer, W. Steinchen, S. Urban, Y. Liu, G. Bange, M. Thanbichler, ParB-type DNA segregation proteins are CTP-dependent molecular switches. Cell 179, 1512–1524.e15 (2019). 31835030 10.1016/j.cell.2019.11.015

[75] L. Schrödinger, W. DeLano, The PyMOL Molecular Graphics System, Version 3.0 Schrödinger LLC.

[76] G. Brändén, R. Neutze, Advances and challenges in time-resolved macromolecular crystallography. Science 373, eaba0954 (2021). 34446579 10.1126/science.aba0954

[77] R. Neutze, R. J. D. Miller, Energetic laser pulses alter outcomes of x-ray studies of proteins. Nature 626, 720–722 (2024). 38355996 10.1038/d41586-024-00233-2

[78] T. R. M. Barends, A. Gorel, S. Bhattacharyya, G. Schirò, C. Bacellar, C. Cirelli, J. P. Colletier, L. Foucar, M. L. Grünbein, E. Hartmann, M. Hilpert, J. M. Holton, P. J. M. Johnson, M. Kloos, G. Knopp, B. Marekha, K. Nass, G. Nass Kovacs, D. Ozerov, M. Stricker, M. Weik, R. B. Doak, R. L. Shoeman, C. J. Milne, M. Huix-Rotllant, M. Cammarata, I. Schlichting, Influence of pump laser fluence on ultrafast myoglobin structural dynamics. Nature 626, 905–911 (2024). 38355794 10.1038/s41586-024-07032-9 PMC10881388

[79] Q. Bertrand, P. Nogly, E. Nango, D. Kekilli, G. Khusainov, A. Furrer, D. James, F. Dworkowski, P. Skopintsev, S. Mous, I. Martiel, P. Börjesson, G. Ortolani, C. Y. Huang, M. Kepa, D. Ozerov, S. Brünle, V. Panneels, T. Tanaka, R. Tanaka, K. Tono, S. Owada, P. J. M. Johnson, K. Nass, G. Knopp, C. Cirelli, C. Milne, G. Schertler, S. Iwata, R. Neutze, T. Weinert, J. Standfuss, Structural effects of high laser power densities on an early bacteriorhodopsin photocycle intermediate. Nat. Commun. 15, 10278 (2024). 39604356 10.1038/s41467-024-54422-8 PMC11603225

[80] A. Vallejos, G. Katona, R. Neutze, Appraising protein conformational changes by resampling time-resolved serial x-ray crystallography data. Struct. Dyn. 11, 044302 (2024). 39056073 10.1063/4.0000258 PMC11272219

