# Supplementary material for: Capturing structural intermediates in an animal-like cryptochrome photoreceptor by time-resolved crystallography
Source: Sci Adv. 2025 May 16;11(20):eadu7247. doi: 10.1126/sciadv.adu7247 (PMC12083526; doi:10.1126/sciadv.adu7247)
Supplement: Supplementary file 1 — Supplementary Text S1 to S3 Figs. S1 to S23 Tables S1 to S4 Legends for Supplementary Coordinates Files S1 and S2 Legend for movie S1 References [file sciadv.adu7247_sm.pdf]

Science Advances
AAAS

Supplementary Materials for

Capturing structural intermediates in an animal-like cryptochrome photoreceptor by time-resolved crystallography

Manuel Maestre-Reyna *et al.*

Corresponding author: Manuel Maestre-Reyna, mmaestre@ntu.edu.tw; Ming-Daw Tsai, mdtsai@gate.sinica.edu.tw; Junpei Yamamoto, yamamoto.junpei.es@osaka-u.ac.jp; Lars-Oliver Essen, essen@chemie.uni-marburg.de

Sci. Adv. **11**, eadu7247 (2025)
DOI: 10.1126/sciadv.adu7247

The PDF file includes:

Supplementary Text S1 to S3
Figs. S1 to S23
Tables S1 to S4
Legends for Supplementary Coordinates Files S1 and S2
Legend for movie S1
References

Other Supplementary Material for this manuscript includes the following:

Movie S1

Supplementary texts

Supplementary text S1: Estimating photon dosage

In order to estimate photon dosage, we considered our previous report (*28*), in which we showed that upon illumination of a grease-embedded 75 µm diameter lysozyme filament, ~32% of incoming light was scattered away by the air-filament interface. Meanwhile, scattering within the grease filament itself was negligible (*28*). With a nominal pump laser power density of 0.21 GW/cm^2, we estimated that interaction with the air-grease interface reduces power density to 0.143 GW/cm^2, which, for a pulse duration of 3 ns, corresponded to an energy density of 428.4 mJ/cm^2. Considering the energy of a 450 nm photon (4.41×10^{-19} J) and the cross-section of oxidized FAD at 450 nm (4.28×10^{-17} cm^2) we estimated a nominal photon dosage of 36 photons per pulse and chromophore.

Next, we considered the effect of crystal orientation towards the pump beam. We estimated an average crystal size of 50x10x10 μm^3 (fig. S15) of which a cylindrical volume with 2 µm diameter would be probed by the XFEL pulse. Therefore, if the crystal is exposed along any of the two short axes, light will be attenuated along a path of ~4 µm on average (half of crystal axis length minus probed volume radius), while, if along the long axis, ~24 µm. Considering the FAD's extinction coefficient at 450 nm and an *in crystallo* *Cr*aCRY concentration of 13 mM, the pump beam is predicted to be attenuated by ~12.5% if oriented along the short axis, while ~53.6% if oriented along the long axis, giving nominal photon dosage range of between 32 to 17 photons per pulse and chromophore.

Given this high nominal photon dosage, it is worth interrogating what the probability of simultaneous multi-photon events is for *Cr*aCRY under our experimental conditions. If the lifetime of the excited FAD^* state is estimated to be ~0.4 ps due to relaxation via electron transfer from W399 (Fig. 1)(*21*), a 0.14% conditional probability arises for a multiphoton event given 32 photon absorption events per 3 ns pulse.

Supplementary text S2: Power titration and effect of laser power on *Cr*aCRY kinetics

Although the relatively high nominal photon dosage as calculated in Supplementary text 2 may appear excessive, it is not outside the common norm for TR-SFX experiments (*76*). In fact, it is well documented that, for poorly understood reasons, TR-SFX experiments which are run in the nominal multi-photon regime still produce biologically relevant results (*77*). In this context, we performed both TR-SFX power titrations at power densities ranging from 0.002 to 0.21 GW/cm^2 (0.0014 to 0.143 GW/cm^2 after correction for grease-air interface scattering, fig. S5B and C). As an indicator, we compared the integrated negative DED(*3µs-dark*) signal around E384 vs. power density level. Here, we observed that E384-associated DED signals were far from being saturated even at 0.143 GW/cm^2. Given the microsecond conformational change time-frame of E384 (Fig. 3B), we propose that it accurately represents the power dependency of the overall *Cr*aCRY structural kinetics at the 3 µs time-point.

Meanwhile, to understand the effect of *in crystallo* high power densities on FAD photoreduction and protonation kinetics, we performed the experiments described in section "Time-resolved *in crystallo* optical spectroscopy (TR-*ic*OS)" and exposed on Fig. 2D and fig. S5 and S12 at power densities between 0.03 and 0.16 GW/cm^2. Power density was not adjusted for scattering here, as, as previously shown, scattering through the sample holder window is negligible (*28*).

Based on these experiments, and in good agreement with recent studies which confined structural multiphoton effects to the sub-ps time-scale (*78, 79*), we could observe no power-dependent differences in the kinetic behavior of the sample within the µs time resolution of the TR-*ic*OS instrument (fig. S5A). Additionally, *in crystallo* kinetics (fig. S5 and S12) agree well with transient spectroscopy (TA) data of *Cr*aCRY in solution in the ns to ms time-scale (*21*). Importantly, while the ns-ms TA experiments were not measured under single-photon conditions,

they in turn overlapped well with fs to ns ultrafast data from the same study, which were acquired in the single photon absorption regime (*21*). To us this signifies that, at least for the slower processes observed in this work, i.e. TPP conformational changes (µs-time scale), $FAD^{\bullet-}$ protonation (ms-time-scale) and C-terminal order-disorder transition (subsecond time scale), high power densities as used here produce biologically relevant results. Another possible effect of high photon dosage which cannot be discarded is a change in the reaction speed/kinetic or unit cell parameters derived from conversion of photon energy into heat *(79)*. Here, we could not detect any heat effects on the crystal lattice, as TR-SFX datasets were highly isomorphous (table S1-2). Additionally, the good agreement between the high photon dosage TR-SFX structures, *in crystallo* spectroscopy, and single photon *in solution* spectroscopy suggests that the kinetics of FAD radical stabilization and protonation are not significantly affected. Meanwhile, the kinetics of unfolding of helix α22 presented here correlate well with results derived from time-resolved mass spectrometry (*20*). In both cases the order-disorder transition occurs in the second timescale, thus suggesting that protein motion is also unaffected by possible thermal effects or even, surprisingly, confinement within the crystal lattice.

Only stabilization of $FAD^{\bullet-}$ by N395 (ns-time scale), which falls below the resolution limit of the TR- *ic*OS experiments, may conceivably be affected by such multi-photon effects. Future work in the femto- to nanosecond time-scale will have to address this issue of $FAD^{\bullet-}$ stabilization kinetics, as multi-photon effects pose a challenge (*78*).

Supplementary text S3: Impact of occupancy choice on dFoCC refinement

During TR-SFX experiments, the occupancy of the photoactivated state does not normally reach 100% due to various, poorly understood factors (*79*). However, occupancy must be determined to accurately refine the target structure. For example, Vallejos et al. (*80*) recently analyzed TR-SFX data of bacteriorhodopsin, assuming various occupancy values, and demonstrated that occupancy choice significantly influences the refined model structure when using standard refinement procedures (*80*). This becomes particularly critical here, as recent work by Bertrand et al. (*79*) showed that sample heating due to high pump power may lead to an underestimation of the occupancy. While we estimated the occupancy by a well-established method (*26*, *28*, *48*) as shown in fig. S16, we also used real space correlation coefficient refinement (dFoCC) to determine the positions of several residues (see supplementary methods section "dFoCC and refinement of reaction coordinates"). Since the dependency of dFoCC refinement on occupancy has not been rigorously assessed, we decided to test how different occupancies affected it. Given their prominence in our conclusions, we targeted the occupancies and refined structures of N395 and D321 at 30 µs (16.67% occupancy, N=12). Here, we tested both how moderate over- or underestimation of the occupancy by 30% affects our models (21.67%, and 11.67% occupancy, corresponding to N=9.23 and N=17.14) and what gross occupancy miscalculation would do (100%, and 8%, i.e. N=2 and N=25, respectively).

Briefly, our overall conclusion is that dFoCC is very robust regarding occupancy estimation errors (fig. S21), as overestimation had almost no effect, while underestimation resulted in a quick decay of the converged correlation coefficient value.

In detail, the plots of the correlation coefficient between DED_c and DED_o maps versus occupancy show that confidence in the produced structure increases until occupancy reaches 16.67% (our proposed "real" occupancy), and that it remains approximately constant upon further occupancy increase (fig. S21A and C). Crucially, all refined models, regardless of assumed occupancy, led to the same interpretation of the data. In the case of N395, all refined structures exhibited the same key structural changes, with a clockwise rotation allowing the Nδ2 atom of N395 to form a hydrogen bond with the N5 atom of FAD (fig. S21B). D321 structures refined at assumed occupancy of 11.67% or higher converged to a similar conformation (fig. S21D). Only when the occupancy of D321 is assumed to be as low as 8% did the refinement produce a different orientation of the D321 side chain. However, the 8% occupancy model was the least trustworthy, as

assessed by its correlation coefficient (CC=0.294). Importantly, just like in all other models, the 8% occupancy D321 model still resulted in a rotation away from D321's interaction partner R492; i.e. it still supported that, upon illumination, the salt-bridge interaction between the two broke. This suggests that dFoCC refinement may result in low quality models if occupancy is underestimated by more than 50% during refinement. Otherwise, dFoCC refinement results appear to be robust against variations in occupancy, probably due to the method's reliance on maximizing the covariance between DED_o and DED_c. In other words, dFoCC convergence results in faithful modeling of DED_o trends, rather than absolute values.

Supplementary Figures

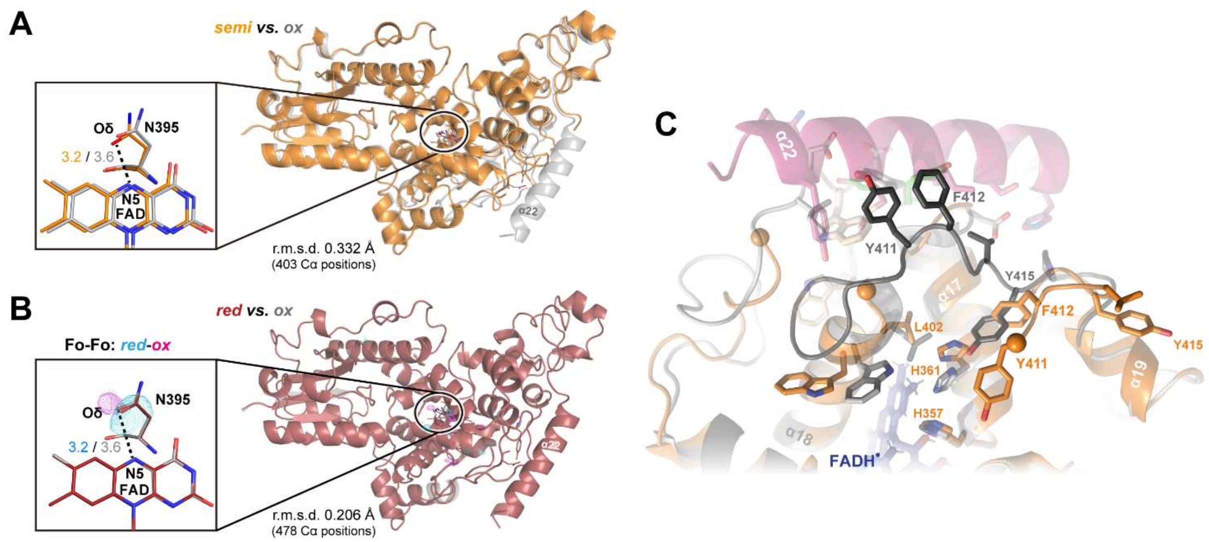

fig. S1. Damage-free *Cra*CRY structures in different FAD redox states. (A) Structure of *Cra*CRY in its FADH$^{\bullet}$ state (orange) that is superimposed to the dark state FAD_{ox} structure (*ox*, grey). The FAD binding site is indicated as black circle and shown with FAD's isoalloxazine and N395 in the inlay (left). (B) Structure of *Cra*CRY in the fully reduced FADH^{-} state (*red*, mauve). Figure arrangement is the same as in (A). To highlight changes between states a 3.5σ contoured DED(*red*-*ox*) map is overlaid (cyan for positive peaks, and magenta for negative ones). (C) Structural details of the FADH$^{\bullet}$ state showing disordering and structural rearrangement of the α18/α19 loop (FAD_{ox} state: dark grey). Colors correspond to Fig, 1C.

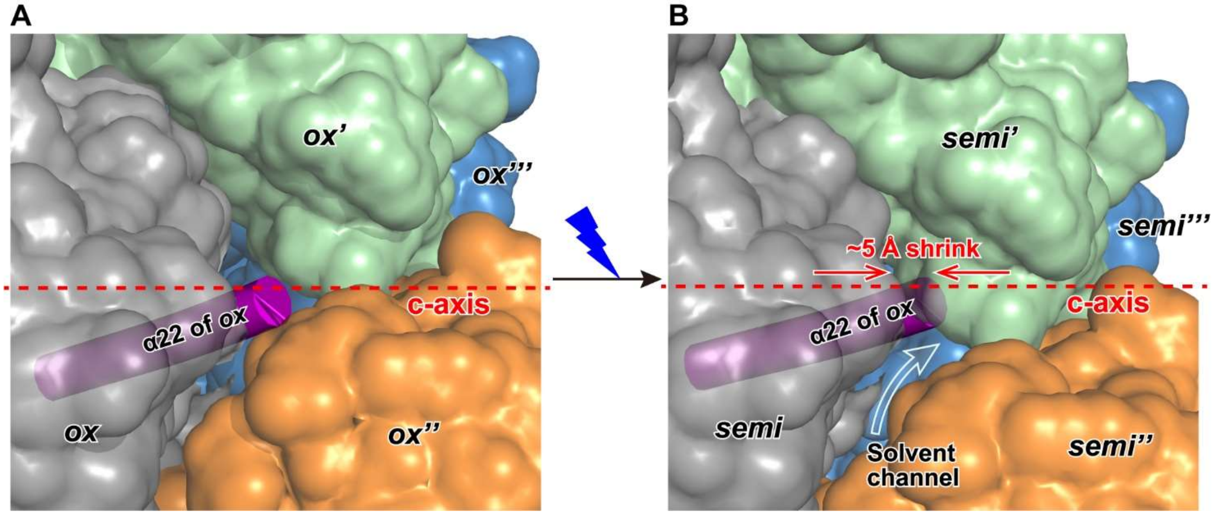

fig. S2. Lattice changes derived from *Cr*aCRY switching from the FAD_{ox} (*dark*) to the $FADH^{\bullet}$ (*semi*) state. (A) Lattice arrangement of *dark* crystals around the c-axis (red dotted line). Four symmetry related asymmetric units are shown as surface representations (*ox* to *ox'''* in grey, green, orange and blue). The surface representation excludes the α22-helix, which is shown as a solid purple cylinder for the *ox* asymmetric unit. Notably, α22 fits tightly into a pocket derived from the interactions between all four asymmetric units, while its C-terminus has access to a solvent channel via a triangular opening between *ox, ox'* and *ox''*. (B) Lattice arrangement of semi crystals around the c-axis (red dotted line). All structures are presented as in (A), and named *semi* to *semi'''*. To highlight the effects of unit cell change on the α22-helix, the *ox* α22-helix is depicted as a purple cylinder. Upon transition to the $FADH^{\bullet}$ state, the c-axis compresses by ~5 Å. As a result, all four asymmetric units approach each other and the α22-helix pocket collapses, shown most prominently here by the fact that *semi'* now overlaps with the *ox* α22-helix. Meanwhile, the triangular opening narrows, but persists. This suggests to us that, during the FAD_{ox}→ $FADH^{\bullet}$ transition, the α22-helix is displaced from its pocket into the solvent channel.

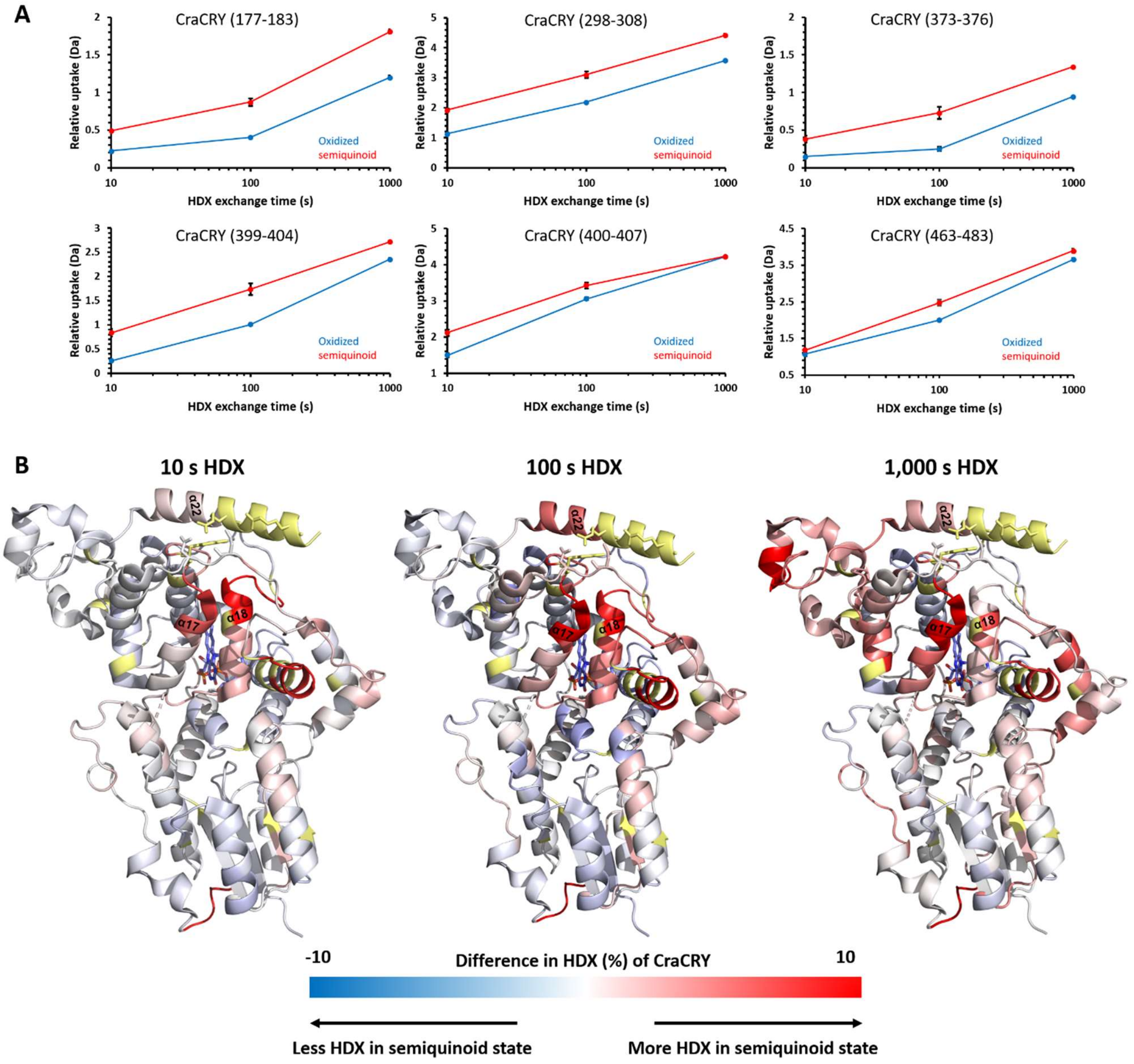

fig. S3. Differences of *Cr*aCRY between oxidized and semiquinoid states in HDX-MS. (A) The HDX analysis of representative *Cr*aCRY peptides (shown by residue numbers in brackets) in both the semiquinoid form (red trace) and the oxidized state (blue trace). The data shown here indicate the mean value ± s.d. of three technical replicates. (B) The difference in HDX of *Cr*aCRY between its oxidized and semiquinoid states is mapped onto the *Cr*aCRY structure (PDB: 5ZM0) at each timepoint of HDX, using the same scale as in A. Residues not covered by peptides are colored in yellow.

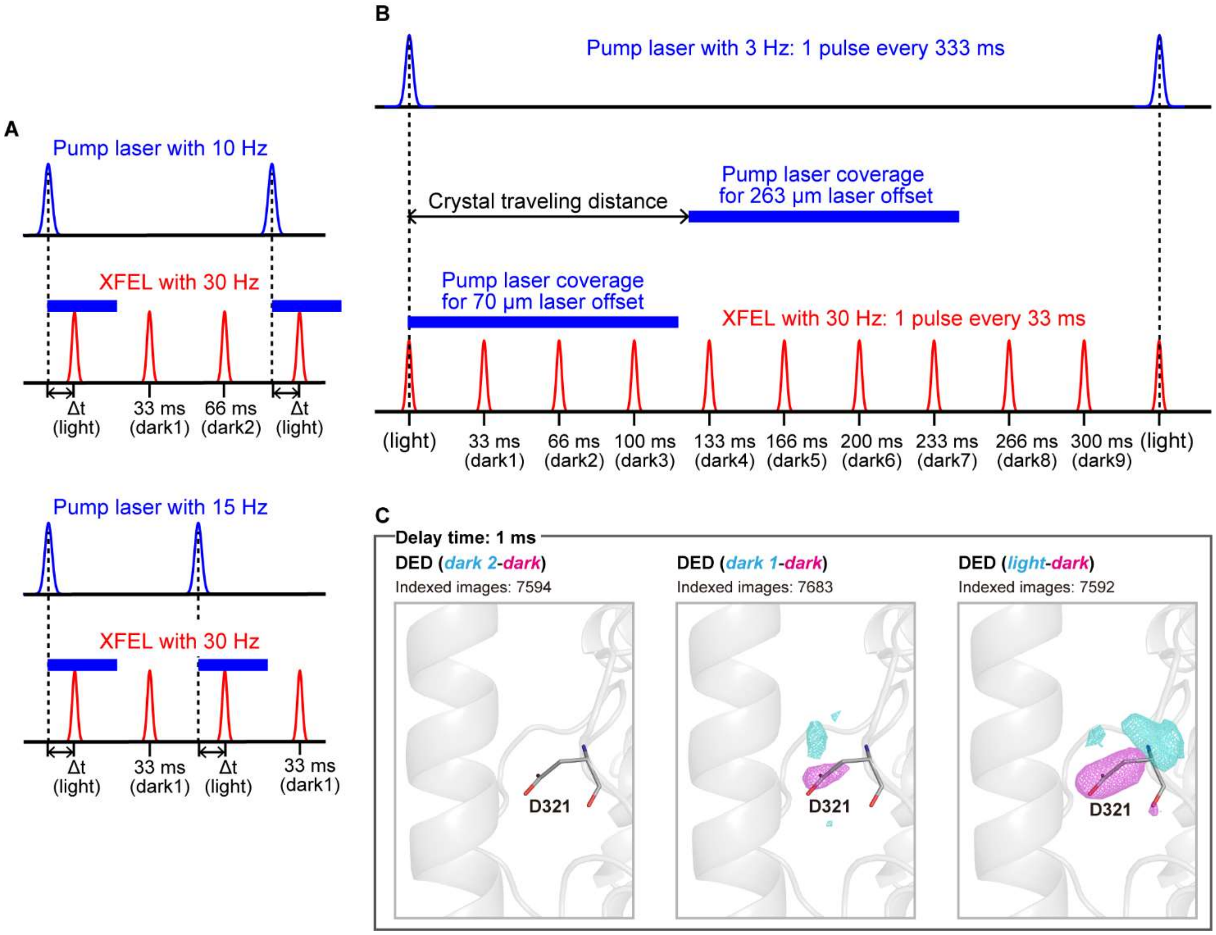

fig. S4. Data collection modes and light contamination analysis. The different pulse sequences used in this work are shown, with XFEL pulses shown as red peaks, while pump pulses at 450 nm in blue. In each panel, a blue horizontal bar covers the XFEL pulses directly within the pump-illuminated region. (**A**) Comparison between 10 Hz and 15 Hz short delay data collection modes. Pump laser frequency and sample flow rate result theoretically in a single XFEL pulse falling within the pump laser illuminated region (light). Meanwhile, at 10 Hz, two dark XFEL pulses (dark1 and dark2) follow each light pulse. At 15 Hz, a single dark XFEL pulse, equivalent to dark1 at 10 Hz, follows each light pulse. Importantly, the subsequent light pulse at 15 Hz occurs at the same position as dark2 at 10 Hz. Therefore, lack of light contamination of dark2 during 10 Hz data collection is proof that, at 15 Hz, illuminated datasets are not light contaminated (see C). (**B**) Long delay data collection mode. Here, 3 Hz pump laser and 30 Hz XFEL frequencies, combined with slow sample flow rate results in several XFEL pulses within the pump-illuminated region (blue horizontal bar). Offsetting the pump-laser from the XFEL beam center allows probing at later delay times. (**C**) Light contamination analysis was performed at a 1 ms delay using the 10 Hz data collection mode (see A). 3σ-contoured DED maps derived from datasets containing ~7600 indexed images vs *dark* are shown, with positive density in cyan and negative in magenta. The C-terminal region of the *dark* structure (grey cartoon) including α22, while D321 is shown as sticks. The most prominent DED signal in all datasets occurred at D321 and could be clearly detected for DED(*light-dark*) (compare with fig. S8), and barely for DED(*dark1-dark*). No DED peaks were observed in DED(*dark2-dark*) maps, indicating that dark2 images are free of light contamination.

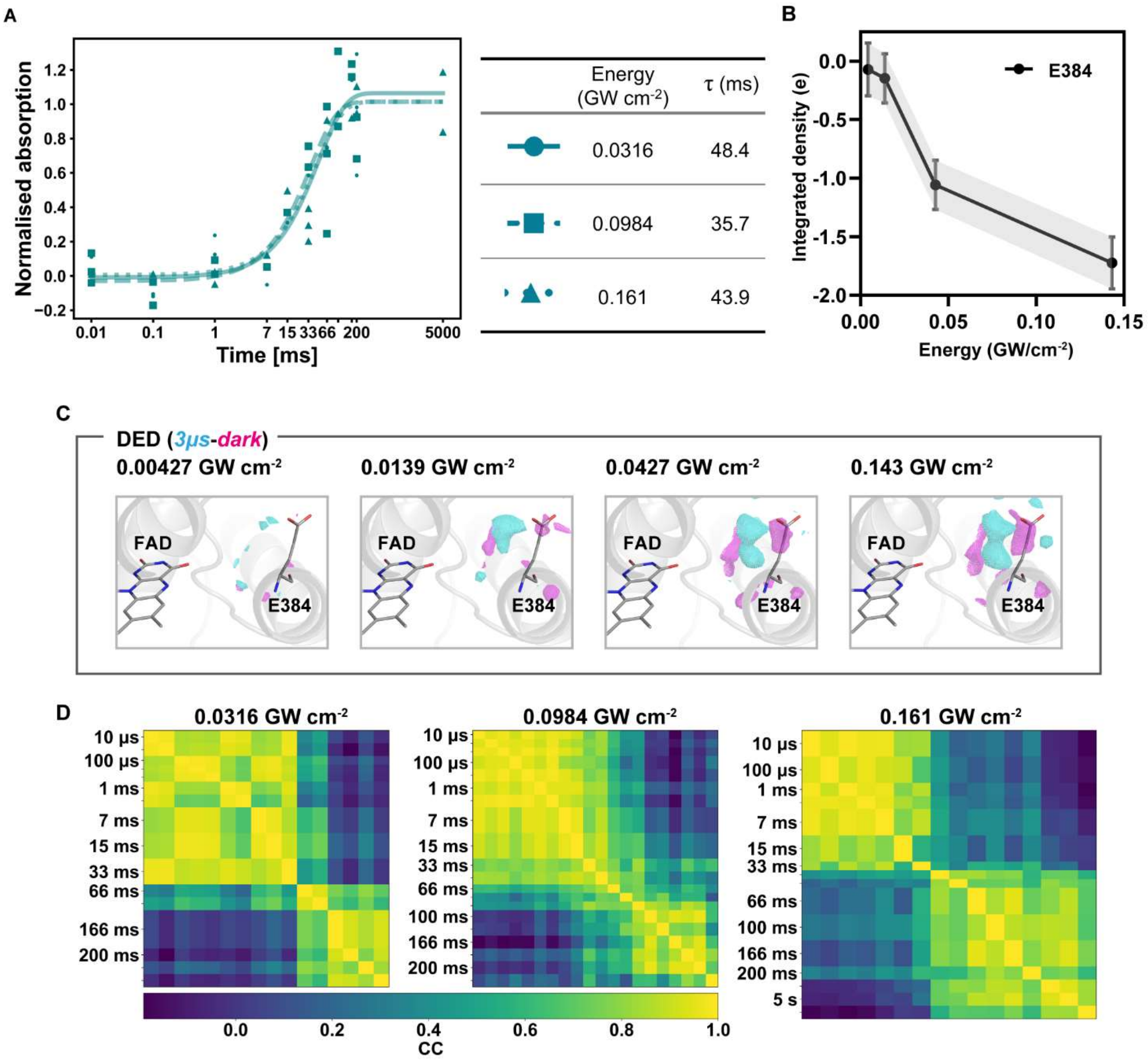

fig. S5. Power titration. Power titration via time-resolved *in crystallo* transient absorption spectroscopy. (**A**) and extraction of negative DED(*3µs-dark*) (**B** and **C**) were performed to determine the effect of high photon dosage on *Cr*aCRY kinetics. (**A**) time traces of FADH$^{\bullet}$ accumulation over time were performed as described under section "*in crystallo* optical spectroscopy" of the supplementary materials and methods. Here, kinetics at three different power density levels (0.0316, 0.0984 and 0.161 GW/cm^2) are shown as circles, squares and triangles, respectively. Fitting by a first order kinetic equation is shown as continuous, dotted, and dashed lines, for each power level. Time-constants for FAD$^{\bullet-}$ depletion and FADH$^{\bullet}$ were within the margin of error (right table) for all three power density levels, indicating that increasing power had no effect on the protonation kinetics of FAD upon photoreduction. (**B**) Plot of extracted negative DED(*3µs-dark*) features around E384 as a function of power density. Here, E384 response showed a steady increase in integrated DED within the noise level of our maps as the power was raised. Noise level was calculated by propagating the map standard deviation (σ-level) over the integrated volume. (**C**) 3σ-contoured DED(*3µs-dark*) maps used to plot (B). Positive density is shown in cyan, while negative in magenta. (D) Correlation maps for all three power levels used for time-resolved *in crystallo* transient absorption spectroscopy. For the purpose of comparing across the three power series, the pixel heights in each map were scaled with time. As with the DED based correlation map shown in Fig. 2C, these present two clusters of correlation which are separated at 33 to 66 ms. To

make comparison with Fig 2C easier, the 0.161 GW/cm^2 map is shown only up to 200 ms in Fig. 2D, while complete (up to 5 s) here.

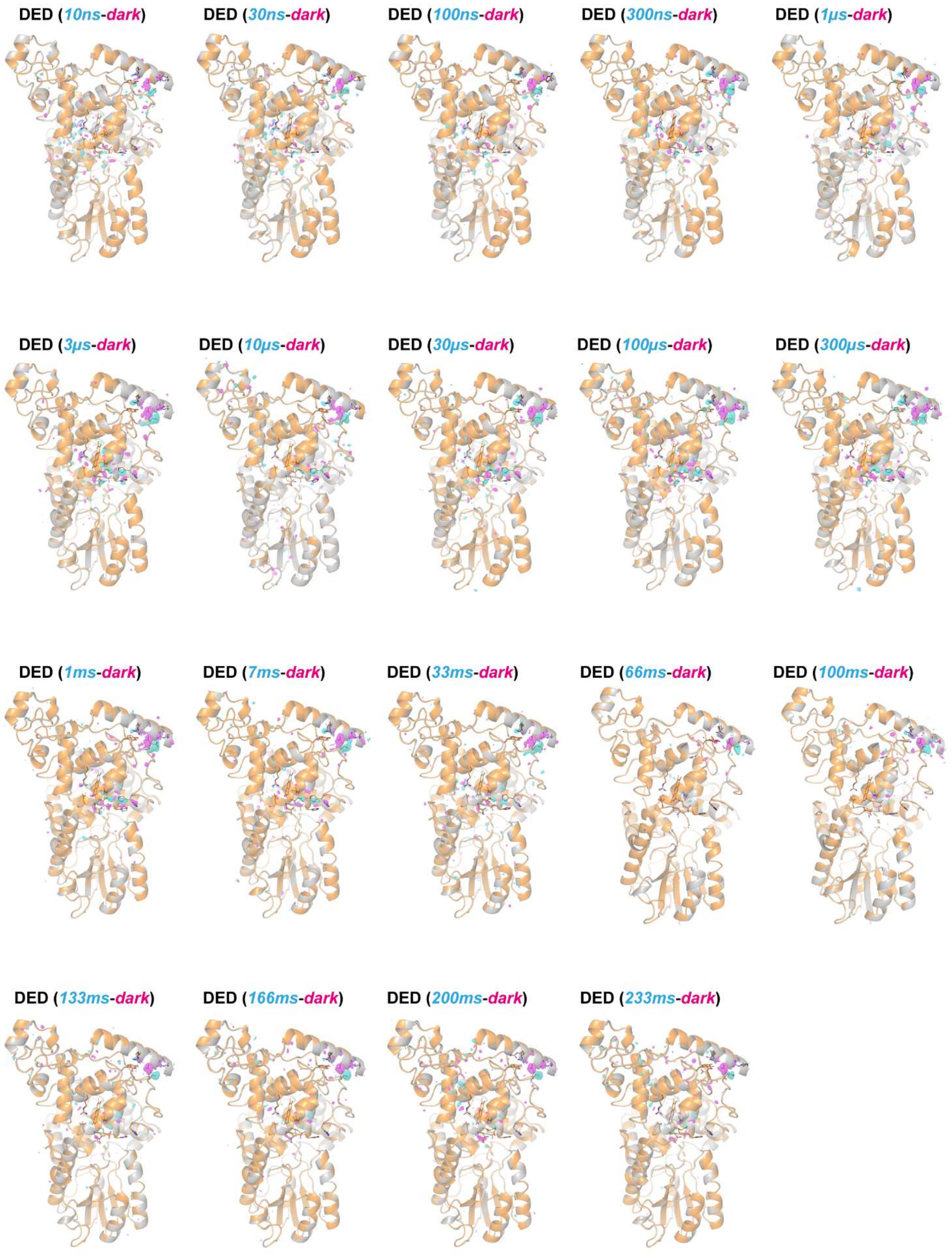

fig. S6. Overall structure with DED maps between time-resolved and dark data. Each time-resolved structure (orange) is superimposed to the FADox state structure (dark, gray) with the observed 3.5σ DED (time-dark) map. The positive and negative DED values are shown in cyan and

magenta, respectively. DED features are prominent in the FAD-binding site, the TPP, and the α22/PHR interface (Fig. 1B).

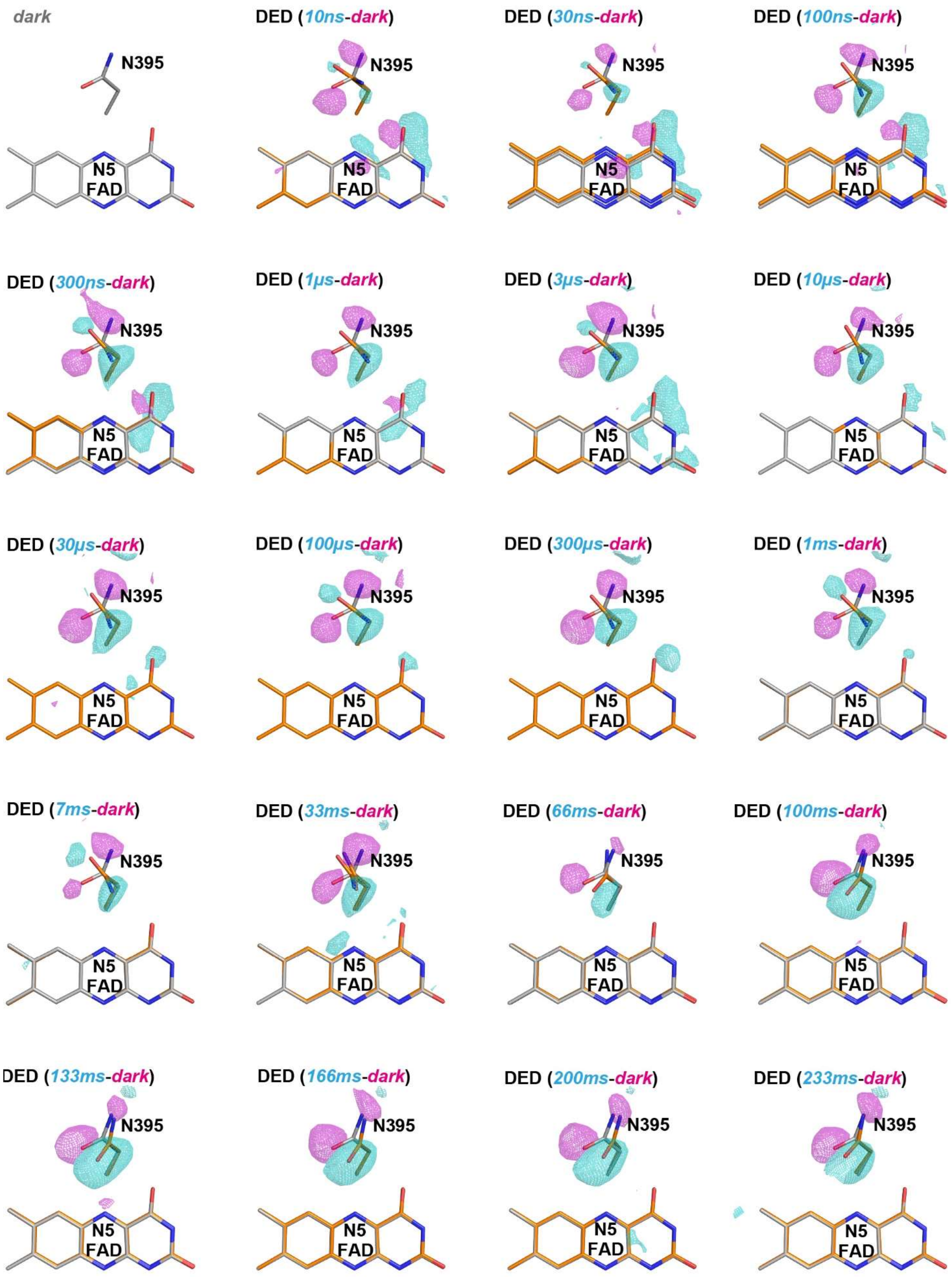

fig. S7. Structural changes in the isoalloxazine moiety of FAD and N395. Each time-resolved structure (orange) is superimposed to the FAD_{ox} state structure (*dark*, gray) with the corresponding 3.5σ DED(*time-dark*) map, where positive and negative DED values are shown in cyan and magenta, respectively. Because relatively strong DED signals are observed around FAD

from 10 ns to 3 μs, the geometry was refined by DED-targeted refinement. From 10 ns to 7 ms, N395 rotates counter-clockwise by about 90 degree and directs its Nδ2 atom towards the N5 nitrogen of the photoreduced $FAD^{\bullet -}$ chromophore. At 33 ms, because both of $FAD^{\bullet -}$ and $FADH^{\bullet}$ states are realized in the protein, double conformations with either Nδ2 or Oδ1 closest to N5 were modelled for N395. From 66 ms, Oδ1 of N395 accepts a hydrogen-bond from the N5 atom of the protonated $FADH^{\bullet}$ chromophore. Conformational models of N395 at all the time-points were produced by DED-targeted refinement. Calculated DED maps based on the refined models are shown in fig. S18 in the same pose.

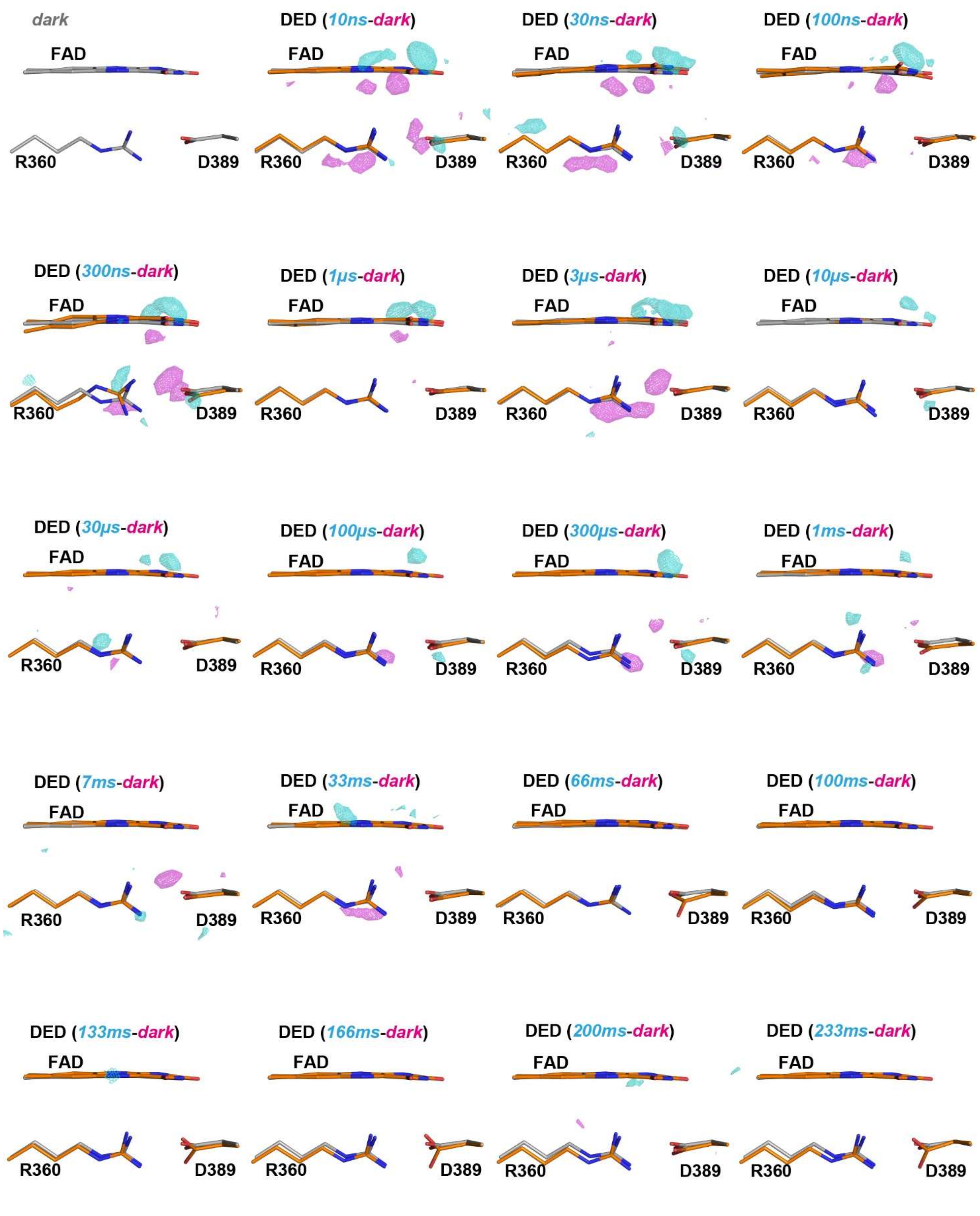

fig. S8. Structural changes in the isoalloxazine moiety of FAD and a salt bridge in FAD binding site. Each time-resolved structure (orange) is superimposed to the FAD_{ox} state structure (*dark*, gray) with the corresponding 3.5σ DED(*time-dark*) map, where the positive and negative DED values are shown in cyan and magenta, respectively. Because relatively strong DED signals are observed around FAD from 10 ns to 3 μs, the geometry was carefully refined by DED-targeted refinement. A salt bridge nearby FAD corresponding to the salt bridge between R360 and D389 in *Cr*aCRY has shown structural changes coupled with the change in the FAD state in a class II PL. However, we did not observe such a strong FAD state correlated movement in the salt bridge in

*Cr*aCRY. Calculated DED maps based on the refined models are shown in fig. S19 in the same pose.

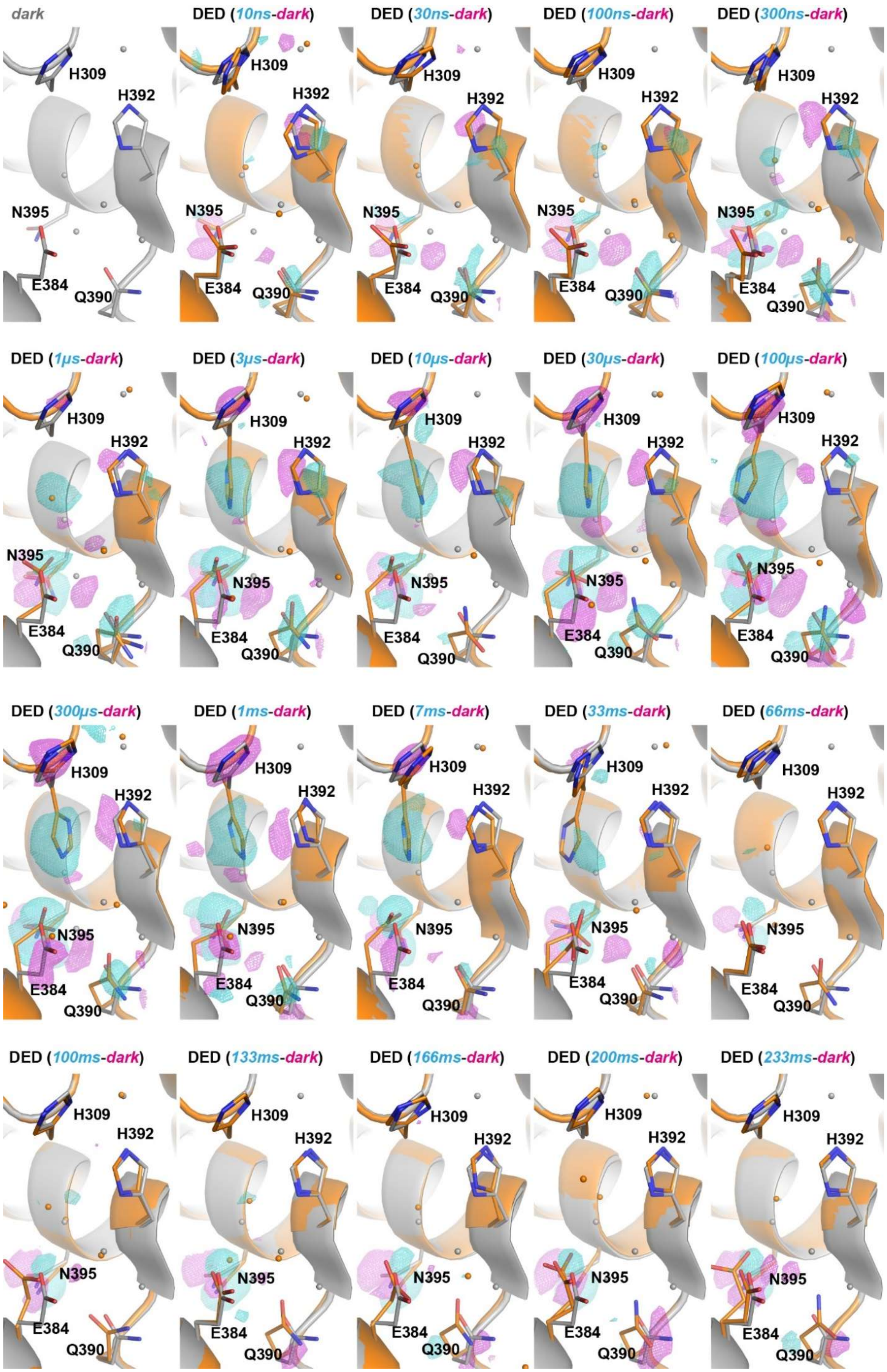
dark
DED (10ns-dark)
DED (30ns-dark)
DED (100ns-dark)
DED (300ns-dark)
DED (1µs-dark)
DED (3µs-dark)
DED (10µs-dark)
DED (30µs-dark)
DED (100µs-dark)
DED (300µs-dark)
DED (1ms-dark)
DED (7ms-dark)
DED (33ms-dark)
DED (66ms-dark)
DED (100ms-dark)
DED (133ms-dark)
DED (166ms-dark)
DED (200ms-dark)
DED (233ms-dark)
H309
H392
N395
E384
Q390

fig. S9. Structural changes for TPP region. Each time-resolved structure (orange) is superimposed to the FAD_{ox} state structure (*dark*, gray) with the corresponding 3.5σ DED(*time-dark*) map, where positive and negative DED values are shown in cyan and magenta, respectively. N395 showing strong DED signals at all the time-points was refined by DED-targeted refinement. Because H309 and E384 show strong DED signals from 3 μs to 33 ms and from 1 μs to 33 ms, respectively, they were subjected to the DED-targeted refinement in these time regions. Q390 and H392 showing some DED signals were also highlighted in sticks. The orange and gray spheres represent water molecules in time-resolved structures and dark one, respectively. Calculated DED maps based on the refined models are shown in fig. S20 in the same pose.

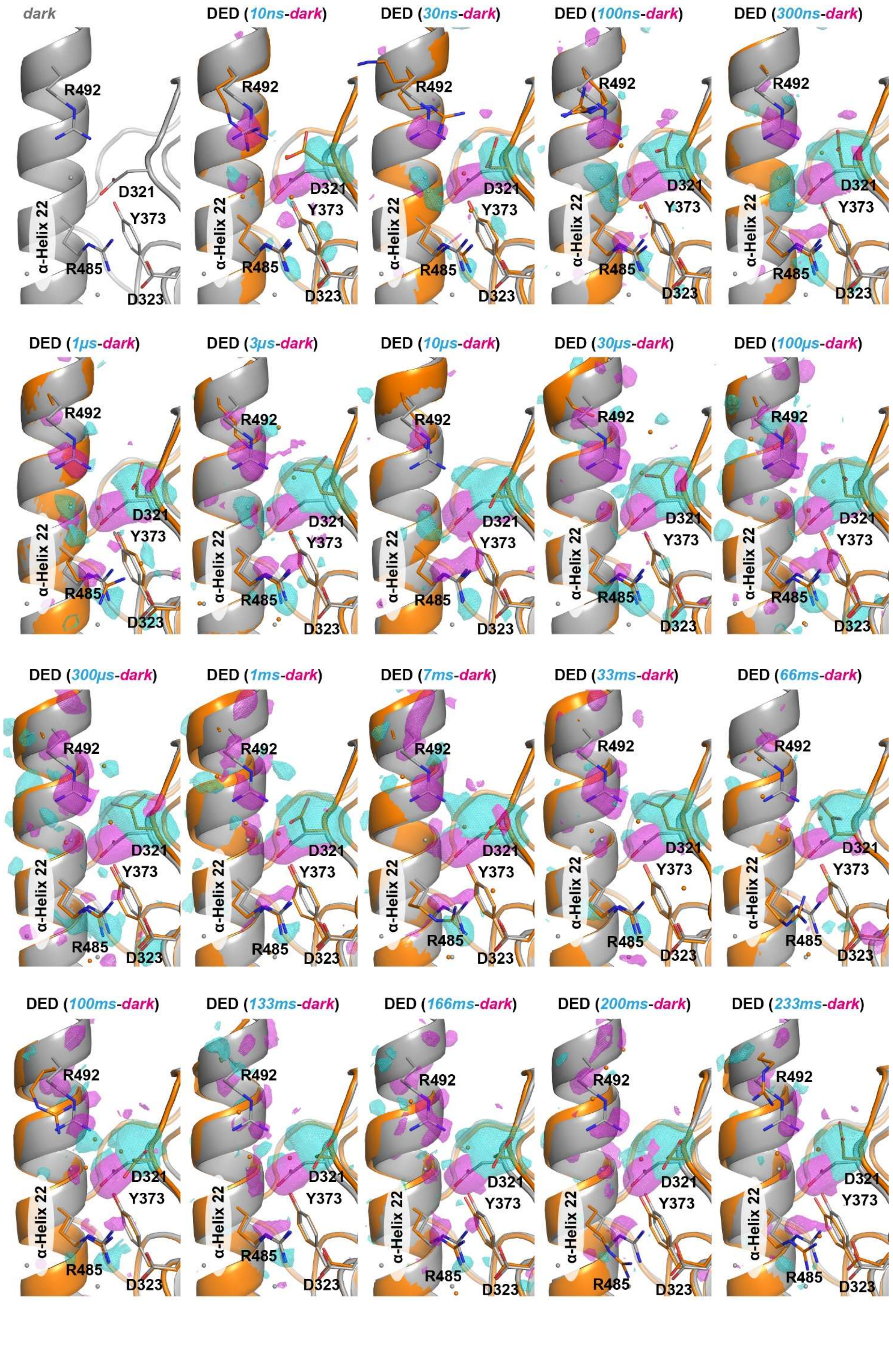

dark
DED (10ns-dark)
DED (30ns-dark)
DED (100ns-dark)
DED (300ns-dark)
DED (1µs-dark)
DED (3µs-dark)
DED (10µs-dark)
DED (30µs-dark)
DED (100µs-dark)
DED (300µs-dark)
DED (1ms-dark)
DED (7ms-dark)
DED (33ms-dark)
DED (66ms-dark)
DED (100ms-dark)
DED (133ms-dark)
DED (166ms-dark)
DED (200ms-dark)
DED (233ms-dark)
R492
D321
Y373
α-Helix 22
R485
D323

fig. S10. Structural changes in C-terminal region. Each time-resolved structure (orange) is superimposed to the dark structure (gray) with the corresponding 3σ DED (*time*-*dark*) map, where the positive and negative DED values are shown in cyan and magenta, respectively. Strong DED peaks around a salt bridge between D321 and R492 were observed at all the observed time-points. The pair of positive and negative DED signals around D321 suggests its flipping movement, which is confirmed by the structural model produced by DED-targeted refinement. Upon the breakage of the salt bridge, the negative peaks around the terminal α-helix 22 get accumulated. Calculated DED maps based on the refined models are shown in fig. S21 in the same pose.

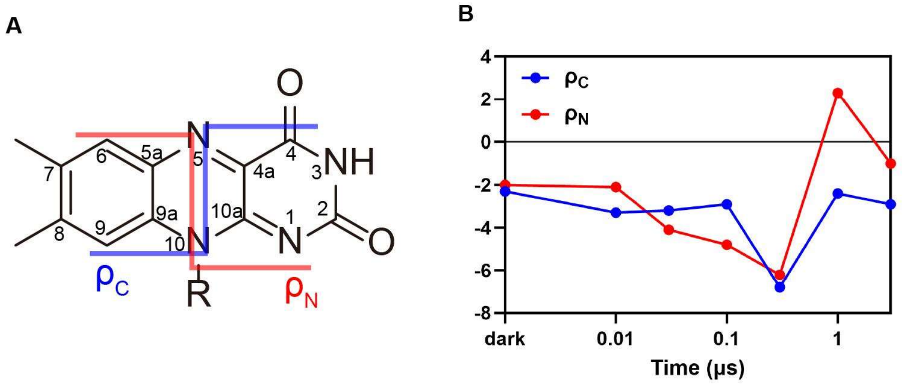

fig. S11. Geometry changes in the isoalloxazine moiety of FAD upon photoreduction. (**A**) ρC and ρN dihedral angles are defined to probe a kinking movement of the isoalloxazine moiety upon photoreduction of FAD. (**B**) The time development of ρC and ρN values for the refined model structures were traced here. Because DED at time-points after 3 μs (from 10 μs to 233 ms) does not show significant (higher than 3σ) negative density peaks around the moiety, we decided to use the same model with the dark model as the isoalloxazine moiety model at these time-points. Therefore, we did not plot the ρC and ρN values beyond 10 μs.

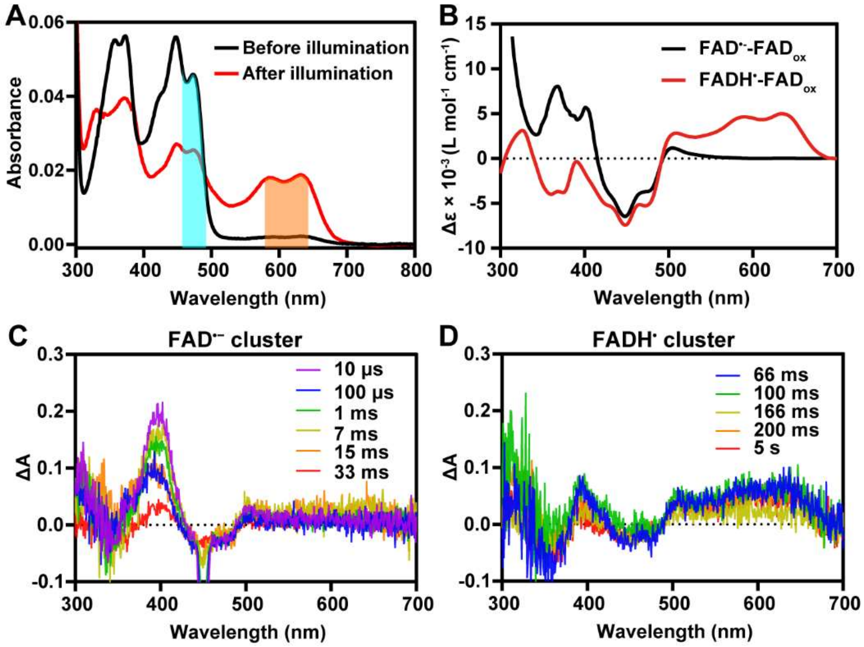

fig. S12. Time-resolved *in crystallo* absorption measurement. (**A**) Steady-state UV/V spectra of *Cr*aCRY before (black, FAD_{ox} state) and after (red, $FADH^{\bullet}$ state) exposure with white light (~430–800 nm) exposure in solution. Photoreduction at pH 5.5, a value mimicking the crystallization condition, was performed without external reductant under aerobic conditions by illuminating the *Cr*aCRY sample for 15 s. Characteristic FAD_{ox} and $FADH^{\bullet}$ regions used for time-resolved *in crystallo* spectroscopy integration are shown in blue and red, respectively. (**B**) $FAD^{\bullet-}$–FAD_{ox} (red) and $FADH^{\bullet}$–FAD_{ox} (red) difference spectra were simulated based on a previously reported absorption spectra in a (6–4) PL in solution (68). Difference spectra between before and after illumination (time-dark) are shown in (**C**) and (**D**). (**C**) The *in crystallo* spectra, recorded from 10 µs to 33 ms, show spectral features similar to the $FAD^{\bullet-}$-FAD_{ox} difference spectrum, indicating that $FAD^{\bullet-}$ predominates until 15 to 33 ms. These correspond to the $FAD^{\bullet-}$ cluster as shown in in Fig. 2D and fig. S5D (**D**) The *in crystallo* spectra from 66 ms resemble the $FADH^{\bullet}$-FAD_{ox} difference spectrum more closely than the $FAD^{\bullet-}$-FAD_{ox} spectrum, suggesting that protonation of $FAD^{\bullet-}$ to $FADH^{\bullet}$ occurs between 33 to 66 ms. This is consistent with the $FADH^{\bullet}$ cluster identified in Fig. 2D and fig. S5D.

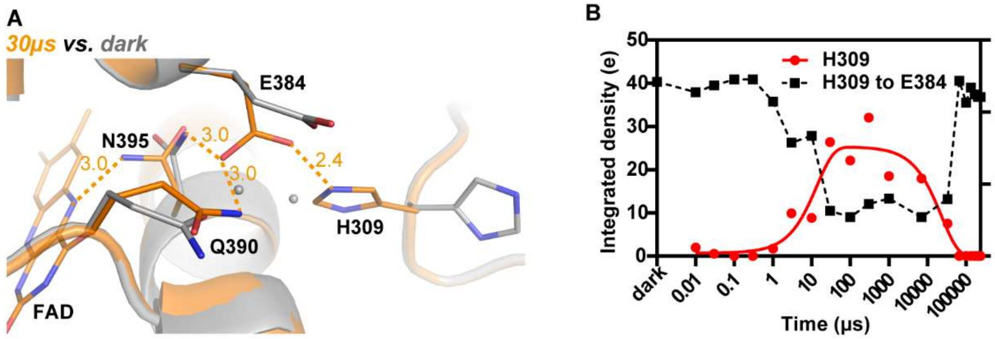

fig. S13. Behavior of H309 during TPP activation-deactivation cycle. (**A**) Detail of the TPP at 30 µs showing the entirety of the activated TPP interaction network. (**B**) H309-to-E384 center of mass distances (black squares) compared to time-dependent accumulation of DED around H309 (red dots). DED data was fitted to a two-step kinetic model (continuous red line) for rate constants.

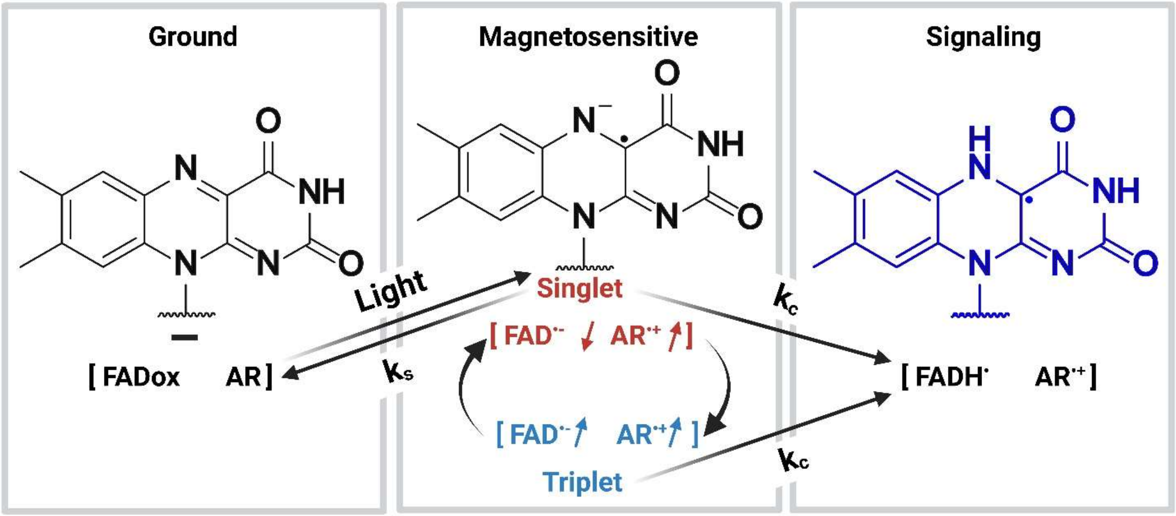

fig. S14. The hypothesized mechanism of the CRY's light-dependent compass. Upon photon absorption, the oxidized FAD_{ox} in the ground state undergoes photoreduction by abstracting a single electron from an aromatic residue (AR) via the inbuilt ET pathway, resulting in the magnetosensitive radical pair (RP) state. Initially, before reaching equilibrium, the $FAD^{\bullet-}$/$AR^{\bullet}$ pair is spin-correlated, and oscillates between the singlet (red) and triplet (blue) states. Only the singlet state may recombine to the initial FAD_{ox}/AR state, as it is spin-anticorrelated (k_s). However, both singlet and triplet state can evolve with equal probability (k_c) towards the signaling state via protonation of $FAD^{\bullet-}$ to $FADH^{\bullet}$. The singlet-triplet interconversion rate depends on the RP's orientation relative to the magnetic field, meaning that the final yield of signaling vs. ground state CRY is also magnetic-field dependent. Finally, because the $FADH^{\bullet}$/$AR^{\bullet}$ pair is extremely long-lived (millisecond time-scale), spin correlation is lost in the signaling state due to singlet-triplet equilibration during its lifetime. Figure adapted from Hore et al. (*8*)

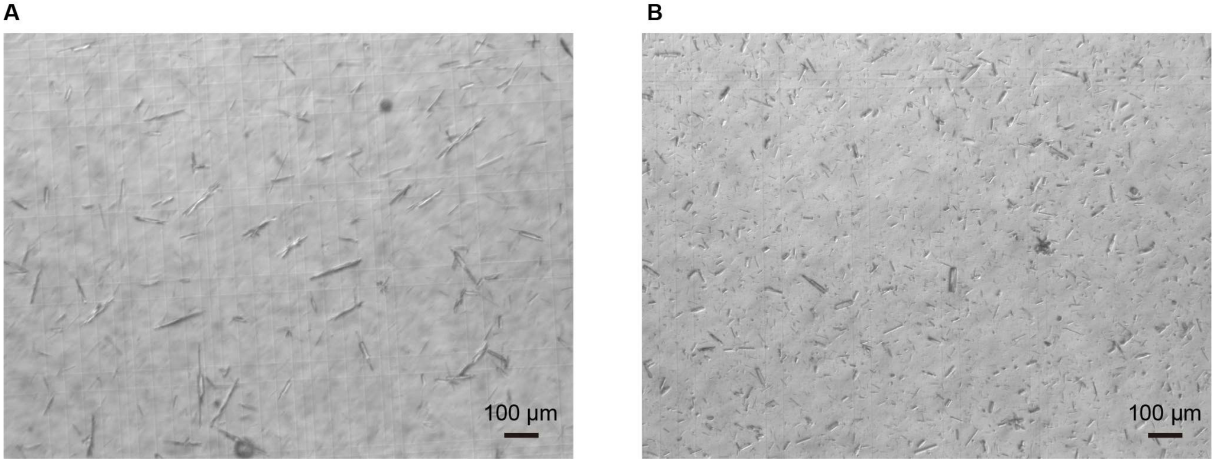

fig. S15. Comparison of crystal size before and after crushing. (**A**) Some *Cra*CRY crystals grow over 100 µm in length which can affect the sample flow of TR-SFX experiments. (**B**) After crushing with homogenizer, crystals over 100 µm were fragmented into smaller crystals.

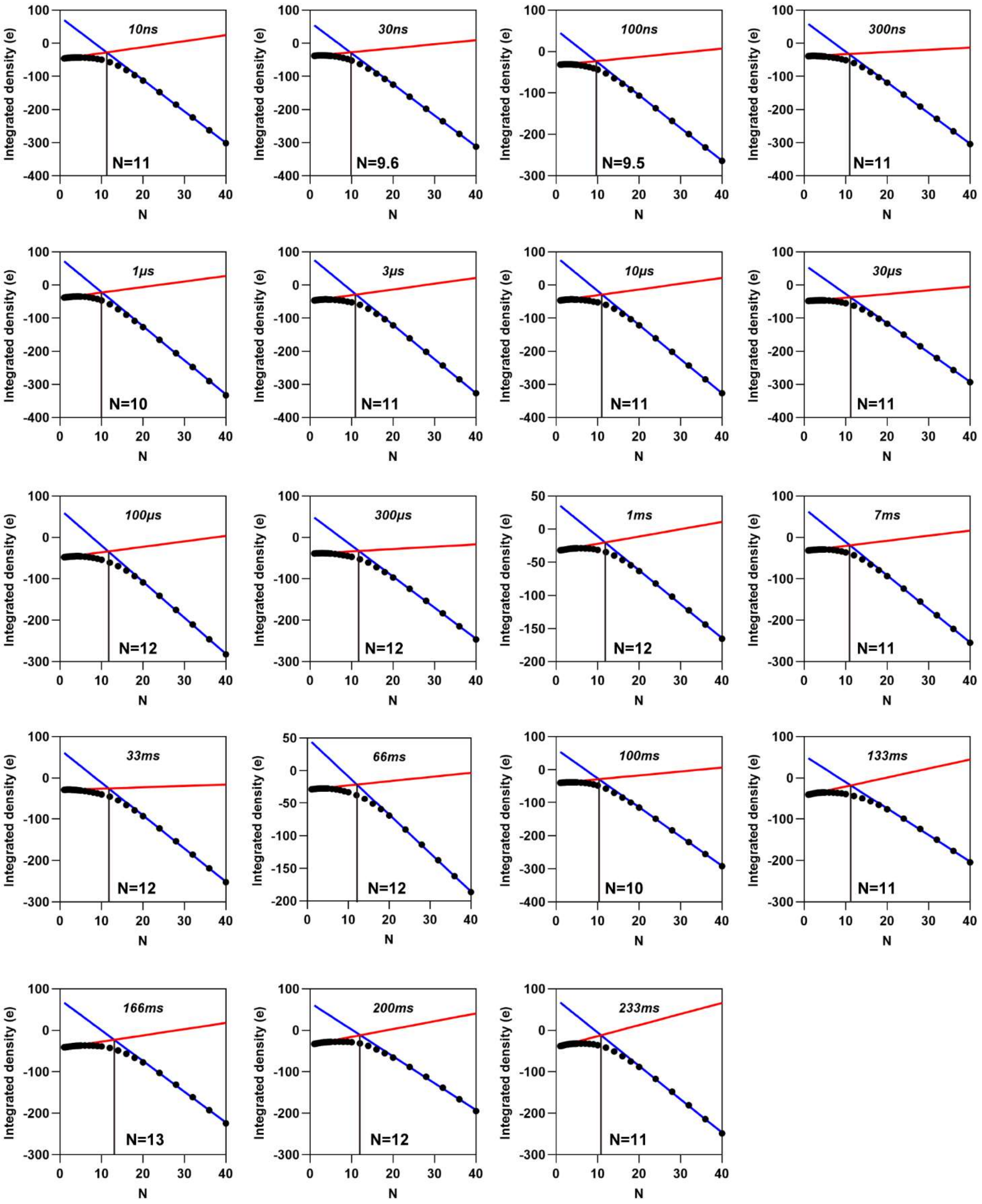

fig. S16. Estimation of the occupancy of the excited species by plotting integrated residual negative density values against N-values (N=2/occupancy). Residual negative densities from the extrapolated structure factor at various N-values (black dot), calculated as |Fc,dark| + (Nw(|Fo,l|-|Fo,dark|)), were extracted around D321. Densities at low N-values, primarily dominated by |Fc,dark|, are approximated by a blue line. On the other hand, densities at high N-values, where Nw(|Fo,l|-|Fo,dark|) has a greater impact, are approximated by a red line. The intersection of the blue and red lines indicates the appropriate N-value for each timepoint, as shown in each panel.

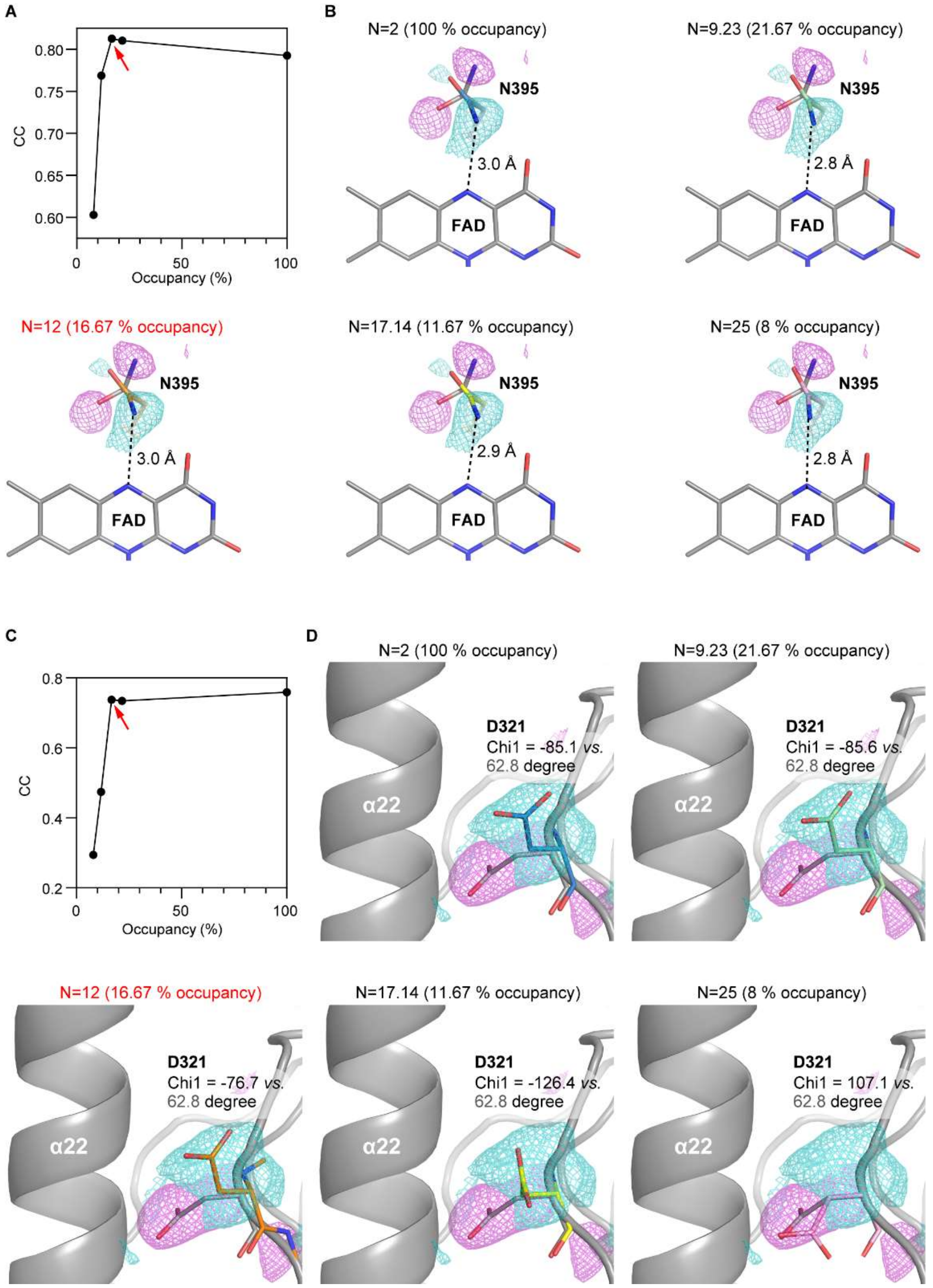

fig. S17. Effects of occupancy choice on dFoCC refinement. dFoCC refinement of N395 and D321 was performed on the 30 µs dataset by assuming various N-values (N=2/occupancy). In (A) and (C), the correlation coefficient between DED_o and DED_c calculated from each dFoCC model is plotted against the assumed occupancy for N395 and D321, respectively. The plots suggest that the correlation coefficient values decrease when the occupancy is grossly underestimated during dFoCC refinement. In (B) and (D), the *Cr*aCRY dark structure was shown in gray, while refined structural models for N395 and D321 at various N-values are color-coded as follows: blue (N=2); green (N=9.23); orange (N=12, written in red to indicate the occupancy used throughout this manuscript); yellow (N=17.14); pink (N=25). In (B), N395 shows a similar conformation across different N-values, as indicated by the interatomic distance between the Nδ2 atom of N395 and the N5 atom of FAD (dotted line). In (D), the flipping of D321 is well reproduced at N=2, 9.23, and 12, as indicated by the Chi1 angles shown in each panel, with the Chi1 angle of the dark structure displayed in gray. Overall, while dFoCC refinement was performed for the deposited structure at N=12 (red arrows and text), the resulting structural models remain robust against different N-

values, suggesting that the choice of N-value has minimal effect on dFoCC refinement results unless the occupancy is largely underestimated.

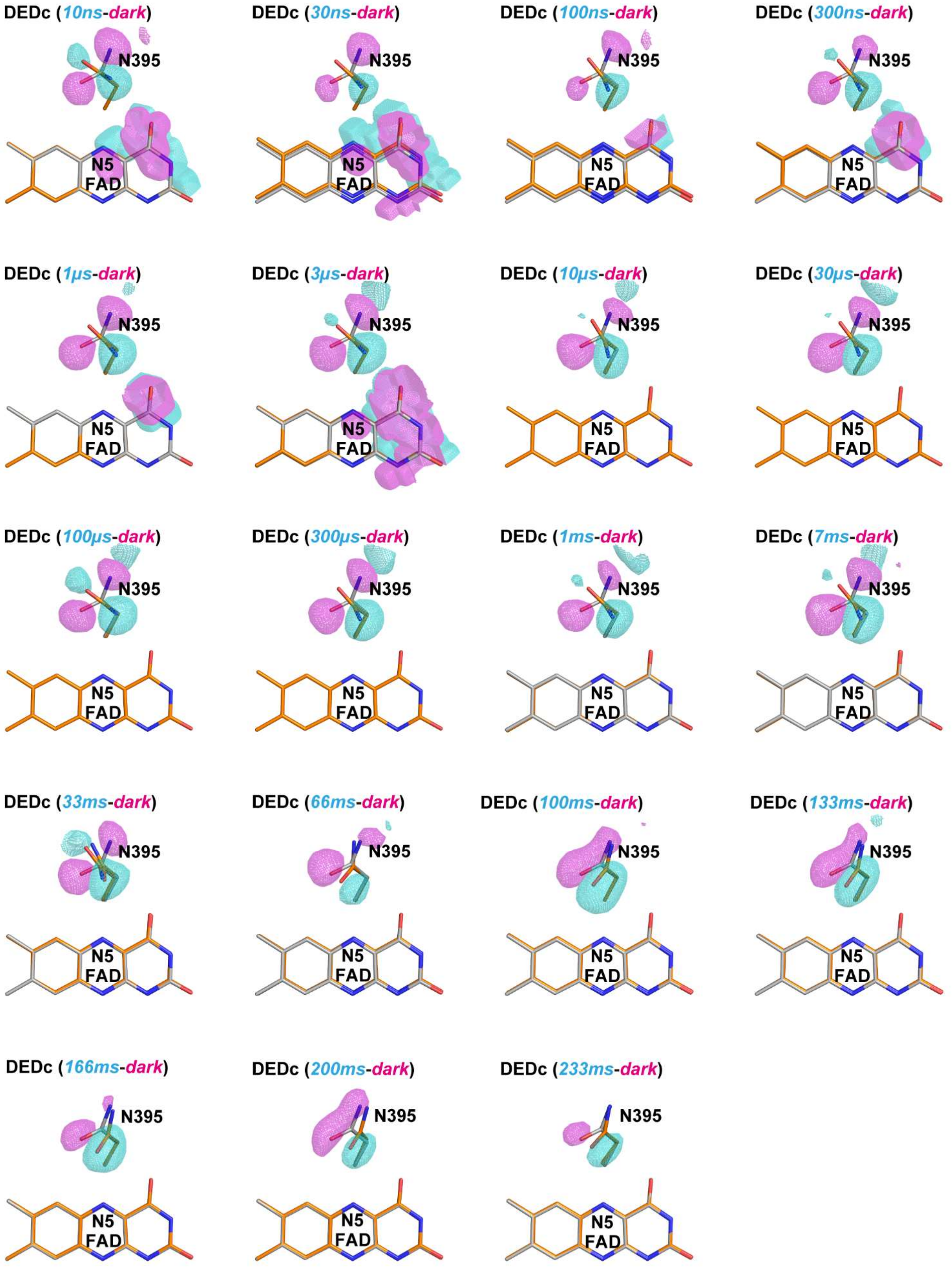

fig. S18. Calculated DED maps for FAD and N395. The calculated difference density maps at 3.5σ level based on the refined models were shown in cyan and magenta for positive and negative peaks, respectively. The signal patterns are overlapped with those observed in DED maps (fig. S7), validating that our model structures are reasonable.

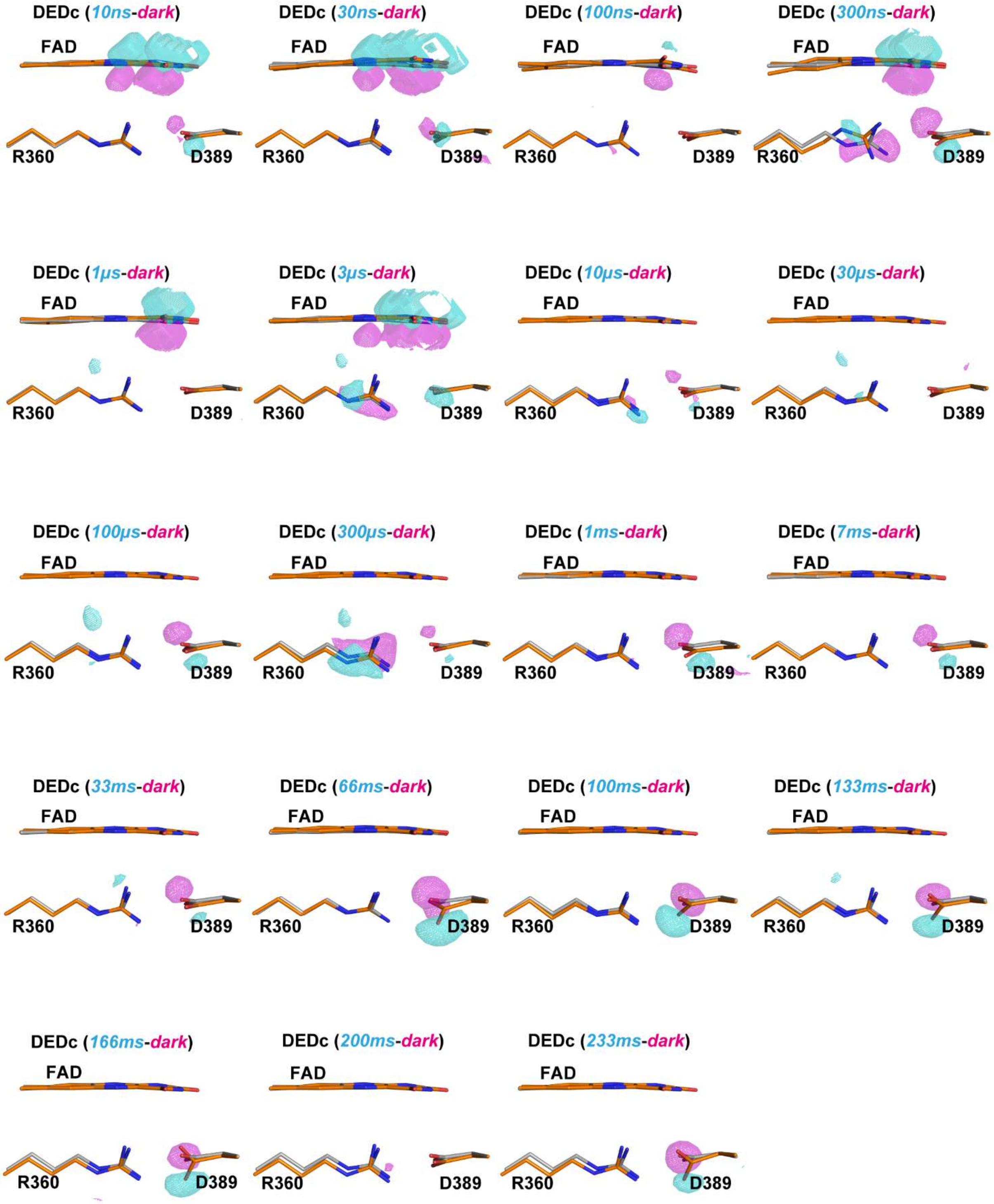

fig. S19. Calculated DED maps around FAD's isoalloxazine moiety and conserved salt bridge next to it. The calculated difference density maps at 3.5σ level based on the refined models were shown in cyan and magenta for positive and negative peaks, respectively. The signal patterns around the isoalloxazine moiety are overlapped with those observed in DED maps (fig. S8), validating that our FAD models are reasonable.

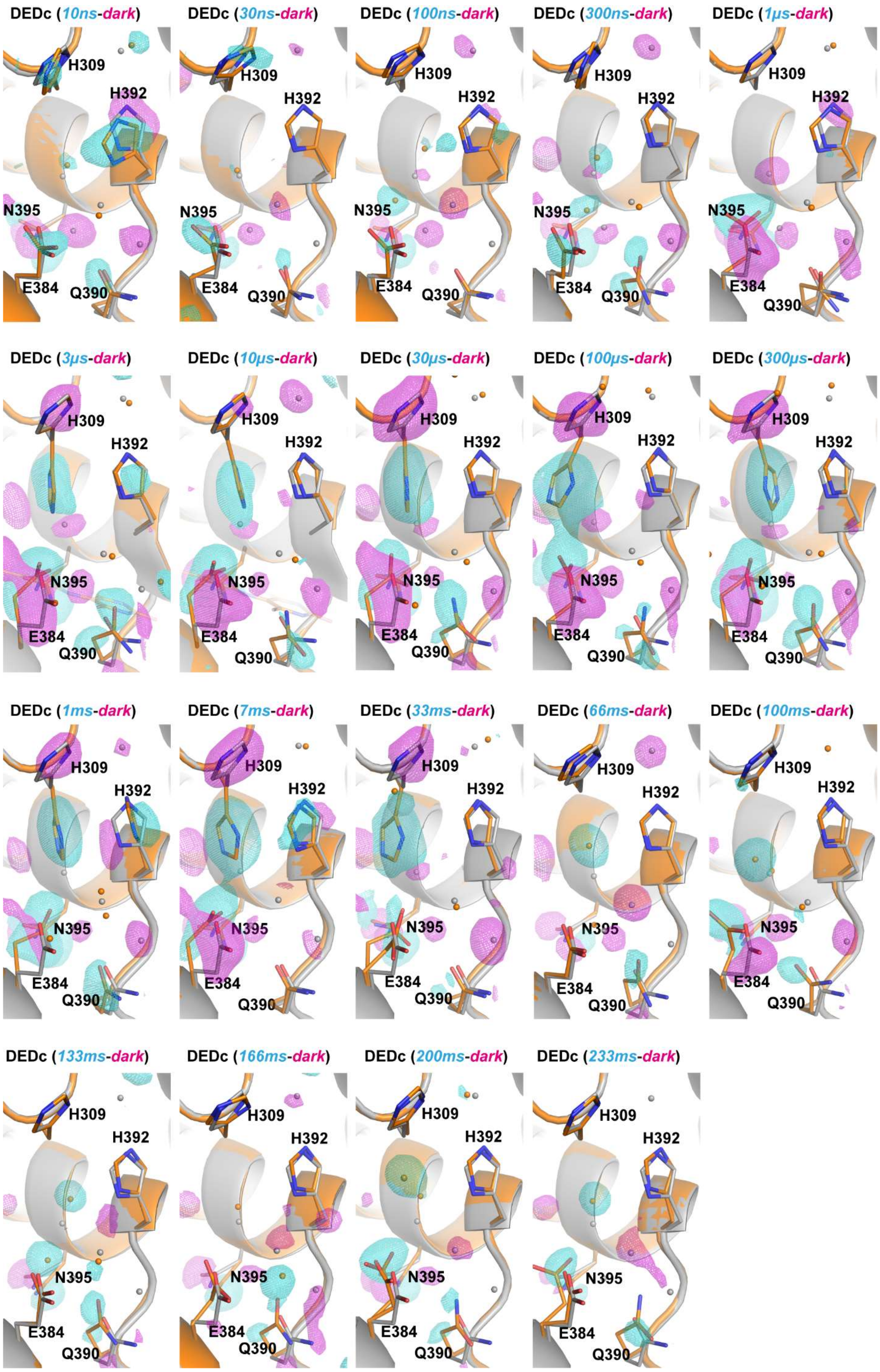
DEDc (10ns-dark)
DEDc (30ns-dark)
DEDc (100ns-dark)
DEDc (300ns-dark)
DEDc (1μs-dark)
DEDc (3μs-dark)
DEDc (10μs-dark)
DEDc (30μs-dark)
DEDc (100μs-dark)
DEDc (300μs-dark)
DEDc (1ms-dark)
DEDc (7ms-dark)
DEDc (33ms-dark)
DEDc (66ms-dark)
DEDc (100ms-dark)
DEDc (133ms-dark)
DEDc (166ms-dark)
DEDc (200ms-dark)
DEDc (233ms-dark)
H309
H392
N395
E384
Q390

fig. S20. Calculated DED maps around TPP. The calculated difference density maps at 3.5σ level based on the refined models were shown in cyan and magenta for positive and negative peaks, respectively. The signal patterns are overlapped with those observed in DED maps (fig. S9), validating that our model structures are reasonable.

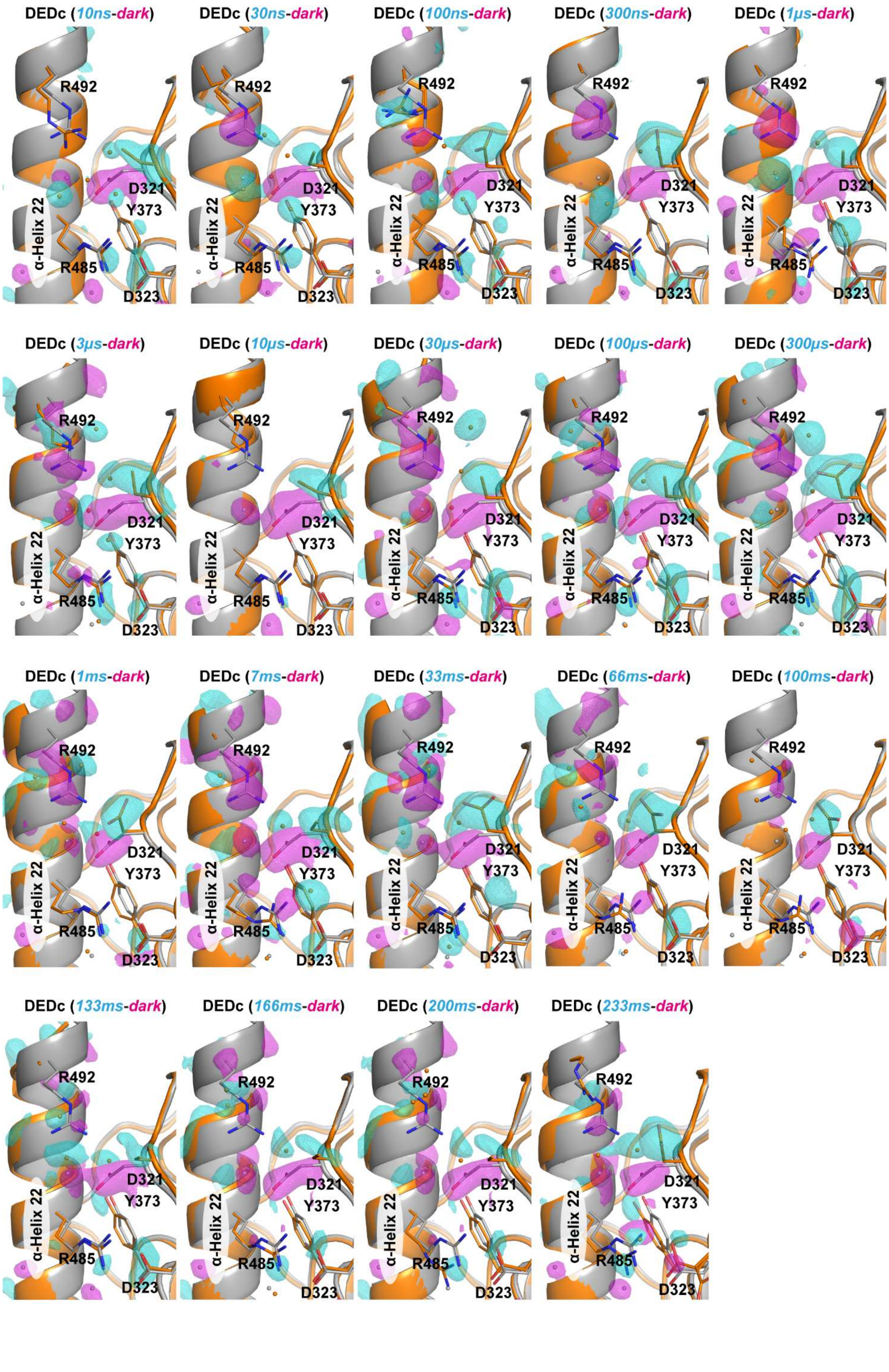

DEDc (10ns-dark)
DEDc (30ns-dark)
DEDc (100ns-dark)
DEDc (300ns-dark)
DEDc (1µs-dark)
DEDc (3µs-dark)
DEDc (10µs-dark)
DEDc (30µs-dark)
DEDc (100µs-dark)
DEDc (300µs-dark)
DEDc (1ms-dark)
DEDc (7ms-dark)
DEDc (33ms-dark)
DEDc (66ms-dark)
DEDc (100ms-dark)
DEDc (133ms-dark)
DEDc (166ms-dark)
DEDc (200ms-dark)
DEDc (233ms-dark)
R492
D321
Y373
α-Helix 22
R485
D323

fig. S21. Calculated DED maps in α22/PHR domain interface. The calculated difference density maps at 3σ level based on the refined models were shown in cyan and magenta for positive and negative peaks, respectively. The signal patterns are overlapped with those observed in DED maps (fig. S11), validating that our model structures are reasonable.

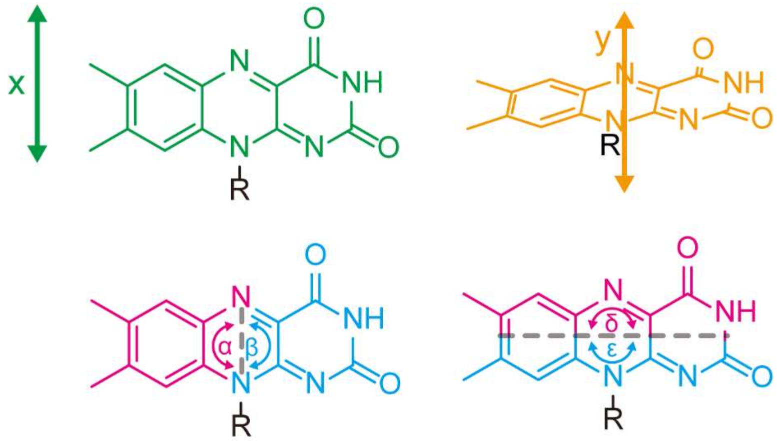

fig. S22 Reaction coordinates x,y,α,β,δ,ε for isoalloxazine dFoCC refinement. X and Y are cartesian coordinates and shown as solid lines. α,β,δ,ε are angles, where the angle is shown as a curved solid line and the axis of rotation as a dotted line. Atoms affected by any given reaction coordinate are color-coded as the corresponding letter in each panel. α and β are defined as rotational translation of a normal vector spanning between each of the color-coded atoms and the vector defined by the N5-N10 axis. Meanwhile, δ and ε are likewise defined as rotational translation along the axis shown in the scheme. The axis itself was constructed by defining a vector with origin at the algebraic mean coordinates of atoms 2 and 3, and end at the algebraic mean coordinates of atoms 7 and 8 (fig. S11). Because all affected atoms (see color-coding) are rotated along the same normal plane, their relative positions remain identical, but their absolute positions change by the corresponding angle.

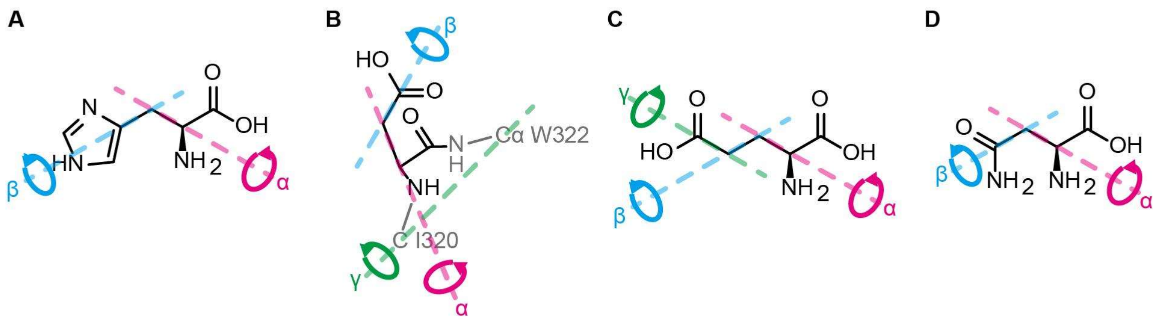

fig. S23 Reaction coordinates for (A) H309, (B) D321, (C) E384, and (D) N395. All amino-acid reaction coordinates correspond to rotational angles (α, β, and γ) affecting specific atoms along axis defined by color coded broken lines. In (A), (C), and (D), side chain atoms were subjected to rotation with the residue main-chain remaining fixed. In (B) side chain atoms were subjected to rotation angles of α and β. Meanwhile, rotational angle γ affected all D321 atoms and the peptide bond N atom of W322. Here, the rotational axis was defined by a vector spanning between the Cα atoms of I320 and W322.

Supplementary Tables

table S1. SFX data statistics for static and TR structures (10 ns to 100 µs). Numbers in parenthesis correspond to highest resolution shell.

**Time point**	**FADH$^{\bullet}$ state**	**FADH^{-} state**	**FAD$_{ox}$ state (*dark*)**	**10ns**	**30ns**	**100ns**	**300ns**	**1µs**	**3µs**	**10µs**	**30µs**	**100µs**
Space group	$P2_12_12_1$											
Unit cell (a, b, c)*	50.59, 65.98, 148.72	50.32, 64.92, 151.54	50.84, 65.60, 153.13									
**Data collection statistics**												
Indexed crystals	22358	41690	87112	24694	29537	25703	29189	26164	25574	21803	23375	26457
Resolution range	41.83-2.13 (2.17–2.13)	41.92-2.00 (2.04–2.00)	33.06-1.64 (1.83–1.64)	32.8-1.75 (1.79–1.75)	32.8-1.81 (1.85–1.81)	33.06-1.87 (1.91–1.87)	33.1-1.83 (1.87–1.83)	32.07-1.83 (1.91–1.83)	32,8-1.77 (1.81–1.77)	32.8-1.85 (1.89–1.85)	33.06-1.78 (1.82–1.78)	32.81-1.78 (1.82–1.78)
**Merging statistics**												
Completeness (%)	99.89 (100)	99.94 (100)	99.98 (100)	99.95 (100)	99.93 (100)	99.94 (100)	99.95 (100)	99.95 (100)	99.95 (100)	99.94 (100)	99.95 (100)	99.94 (100)
Unique reflections	29512 (1688)	34439 (1660)	63708 (3083)	52639 (2596)	47627 (2301)	43294 (2113)	46189 (2258)	43294 (2113)	50888 (2489)	44700 (2229)	50056 (2478)	50127 (2482)
Multiplicity	450 (70)	307 (33)	909 (110)	332 (53)	427 (74)	306 (64)	374 (81)	275 (59)	328 (62)	225 (62)	326 (69)	266 (59)
CC1/2	0.9981 (0.5348)	0.9949 (0.4406)	0.997 (0.506)	0.991 (0.5299)	0.994 (0.527)	0.993 (0.515)	0.993 (0.515)	0.991 (0.515)	0.990 (0.522)	0.987 (0.522)	0.990 (0.536)	0.988 (0.520)
I/σ	6.911 (1.58)	9.119 (1.11)	9.505 (1.37)	6.496 (1.33)	7.65 (1.43)	7.08 (1.43)	7.133 (1.38)	6.76 (1.42)	6.42 (1.41)	5.99 (1.51)	5.91 (1.38)	6.07 (1.41)
**Refinement statistics[%]**												
Resolution range (Å)	41.83-2.15 (2.227-2.15)	31.25-2.1 (2.175-2.1)	32.8–1.64 (1.699–1.64)	30.15–1.95 (2.02–1.95)	27.75–2.0 (2.071–2.0)	30.15–2.0 (2.071–2.0)	30.15–2.00 (2.071–2.00)	30.15–2.0 (2.071–2.0)	30.15–1.95 (2.02–1.95)	30.15–2.0 (2.071–2.0)	27.75–1.95 (2.02–1.95)	30.15–1.95 (2.02–1.95)
Unique reflections	27882 (2733)	29745 (2883)	63626 (6235)	38211 (3782)	35458 (3489)	35460 (3489)	35460 (3489)	35460 (3489)	38210 (3782)	35460 (3489)	38210 (3781)	38205 (3782)
R-work	0.1802 (0.2623)	0.1454 (0.1940)	0.1400 (0.2519)	0.1909 (0.3143)	0.2015 (0.3006)	0.2029 (0.3063)	0.2094 (0.3256)	0.1998 (0.2836)	0.1869 (0.3155)	0.2048 (0.3112)	0.1938 (0.3111)	0.1919 (0.3176)
R-free	0.2197 (0.3659)	0.1722 (0.2318)	0.1574 (0.2881)	0.2236 (0.3450)	0.2259 (0.3456)	0.2172 (0.3082)	0.2316 (0.3424)	0.2246 (0.3288)	0.2166 (0.3516)	0.2348 (0.3582)	0.2293 (0.3454)	0.2256 (0.3356)
Ramachandran outliers	0	0	0	0	0	0	0	0	0	0	0.21	0
RMS (bonds, Å)	0.005	0.01	0.011	0.003	0.003	0.004	0.003	0.003	0.005	0.004	0.004	0.005
RMS (angles, deg)	0.63	0.78	1	0.6	0.59	0.68	0.49	0.61	0.66	0.66	0.61	0.65
Average B factor	57.39	42.46	32.29	14.44	15.36	14.6	13.86	13.09	14.55	12.33	13.32	13.57
Estimated occupancy (%)	100	100	100	18	21	21	20	20	18	18	18	17

*α=β=γ=90°, [%] Refinement statistics of all time-resolved structures are based on extrapolated data

table S2. TR-SFX data statistics for structures (300 µs to 233 ms). Numbers in parenthesis correspond to highest resolution shell.

**Time point**	**300µs**	**1ms**	**7ms**	**33ms**	**66ms**	**100ms**	**133ms**	**166ms**	**200ms**	**233ms**
Space group	$P2_12_12_1$									
Unit cell (a, b, c)*	50.84, 65.6, 153.13	50.84,65.58,153.34		50.84,65.60,153.13		50.74,65.45, 152.95	50.74,65.45,152.95			
**Data collection statistics**										
Indexed crystals	23565	23978	22889	13360	17595	58107	58431	57634	57730	57729
Resolution range	32.8-1.8 (1.84–1.8)	35.59-1.85 (1.89–1.85)	35.59-1.82 (1.86–1.82)	33.16-1.73 (1.77–1.73)	33.1-1.7 (1.73–1.70)	33.02-1.8 (1.83–1.80)	33.02-1.8 (1.84–1.80)	33.02-1.8 (1.84–1.80)	33.02-1.85 (1.89–1.85)	33.02-1.8 (1.84–1.80)
**Merging statistics**										
Completeness (%)	99.94 (100)	99.92 (100)	99.92 (100)	99.95 (100)	99.88 (100)	99.79 (100)	99.79 (100)	99.79 (100)	99.81 (100)	99.8 (100)
Unique reflections	48440 (2388)	44753 (2237)	46926 (2280)	54688 (2683)	48171 (2360)	48171 (2360)	48171 (2360)	48171 (2360)	44448 (2205)	48171 (2360)
Multiplicity	320 (84)	377 (65)	378 (66)	314 (55)	1174 (78.6)	1175 (80.5)	1175 (80.5)	1231 (132)	1313 (200)	1138 (67.2)
CC1/2	0.987 (0.536)	0.992 (0.52)	6.65 (1.33)	0.988 (0.537)	0.999 (0.462)	0.999 (0.447)	0.999 (0.447)	0.999 (0.442)	0.999 (0.553)	0.999 (0.385)
I/σ	5.98 (1.37)	6.65 (1.33)	6.53 (1.36)	6.04 (1.4)	8.78 (1.09)	8.84 (1.11)	8.84 (1.11)	8.69 (1.09)	9.31 (1.39)	8.71 (1.00)
**Refinement statistics[%]**										
Resolution range (Å)	30.15–2.0 (2.071–2.0)	31.59–2.0 (2.071–2.0)	31.59–1.9 (1.968–1.9)	31.59–2.05 (2.123–2.05)	31.59–2.0 (2.071–2.0)	31.52–1.9 (1.968–1.9)	31.52–2.0 (2.071–2.0)	30.09–2.2 (2.279–2.2)	27.67–2.15 (2.227–2.15)	27.67–2.2 (2.279–2.2)
Unique reflections	35460 (3489)	35482 (3489)	41271 (4052)	32989 (3265)	35485 (3490)	41014 (4049)	35245 (3488)	26607 (2616)	28485 (2812)	26610 (2616)
R-work	0.1905 (0.3201)	0.2015 (0.3372)	0.1967 (0.3589)	0.2067 (0.3361)	0.1945 (0.3130)	0.1783 (0.3155)	0.1855 (0.2701)	0.2077 (0.3904)	0.2100 (0.3923)	0.2120 (0.4048)
R-free	0.2224 (0.3779)	0.2239 (0.3628)	0.2225 (0.4136)	0.2282 (0.3410)	0.2277 (0.3326)	0.2189 (0.3815)	0.2187 (0.3116)	0.2198 (0.4425)	0.2282 (0.4000)	0.2380 (0.4057)
Ramachandran outliers	0	0	0	0	0	0	0	0	0	0
RMS (bonds, Å)	0.004	0.003	0.003	0.003	0.003	0.007	0.004	0.007	0.007	0.003
RMS (angles, deg)	0.62	0.59	0.61	0.56	0.61	0.93	0.63	1.04	1.08	0.54
Average B factor	13.53	16.01	15.93	13.72	13.56	16.91	15.19	24.75	26.64	27.78
Estimated occupancy (%)	17	17	18	17	17	20	18	15	17	18

*α=β=γ=90°, [%] Refinement statistics of all time-resolved structures are based on extrapolated data

table S3. TR-SFX data statistics for power titration experiment at 3 µs. Numbers in parenthesis correspond to highest resolution shell.

**Time point**	**3µs**			
**Energy (GW/cm²)**	**0.00427**	**0.013852**	**0.042711**	**0.143147**
Space group	$P2_12_12_1$			
Unit cell (a, b, c)*	51.02 65.82 154.26			
**Data collection statistics**				
Indexed crystals	20072	17035	16066	14986
Resolution range	30.19-1.56 (1.59-1.56)	30.19-1.59 (1.62-1.59)	30.19-1.59 (1.62-1.59)	30.19-1.66 (1.66-1.69)
**Merging statistics**				
Completeness (%)	100 (100)	100 (100)	100 (99.94)	100 (100)
Unique reflections	74327 (3651)	70260 (3443)	70260 (3439)	61880 (3062)
Multiplicity	172 (28.9)	149.11 (27.5)	142.84 (26.0)	155.65 (41.5)
CC1/2	0.985 (0.497)	0.982 (0.516)	0.982 (0.501)	0.980 (0.496)
I/σ	4.75 (1.37)	4.59 (1.34)	4.47 (1.65)	4.79 (1.57)

*α=β=γ=90°, Datasets were only used for difference map generation, not for structural solution.

table S4. Initial and final ranges of reaction coordinates used for DED refinement

		Initial ranges	Final ranges
FAD - isoalloxazine	x	±0.512 Å (0.256 Å)	±0.001 Å (0.001 Å)
	y	±0.512 Å (0.256 Å)	±0.001 Å (0.001 Å)
	α	±12.8° (6.4°)	±0.1° (0.1°)
	β	±12.8° (6.4°)	±0.1° (0.1°)
	ε	±6.4° (3.2°)	±0.1° (0.1°)
	δ	±6.4° (3.2°)	±0.1° (0.1°)
H309	α	±160° (3.2°)	±5° (0.1°)
	β	±160° (3.2°)	±5° (0.1°)
D321	α	±153.6° (12.8°)	±4.8° (0.1°)
	β	±153.6° (12.8°)	±4.8° (0.1°)
	γ	±153.6° (12.8°)	±4.8° (0.1°)
E384	α	±160° (16°)	±1° (0.1°)
	β	±160° (16°)	±1° (0.1°)
	γ	±160° (16°)	±1° (0.1°)
N395	α	±180° (30°)	±4.5° (0.1°)
	β	±180° (30°)	±4.5° (0.1°)

Other Supplementary Materials

Supplementary file 1. Coordinate file produced by refining against the SV0 map, as shown in Fig. 4F. File can be retrieved from https://doi.org/10.5281/zenodo.15192585

Supplementary file 2. Coordinate file produced by refining against the SV1 map, as shown in fig. 4G. File can be retrieved from https://doi.org/10.5281/zenodo.15192585

Movie S1. 3D molecular movie of *Cr*aCRY post-illumination dynamics

REFERENCES AND NOTES

1. Q. Mei, V. Dvornyk, Evolutionary history of the photolyase/cryptochrome superfamily in eukaryotes. *PLOS ONE* **10**, e0135940 (2015).

2. I. H. Kavakli, I. Baris, M. Tardu, Ş. Gül, H. Öner, S. Çal, S. Bulut, D. Yarparvar, Ç. Berkel, P. Ustaoğlu, C. Aydın, The photolyase/cryptochrome family of proteins as DNA repair enzymes and transcriptional repressors. *Photochem. Photobiol.* **93**, 93–103 (2017).

3. I. Chaves, R. Pokorny, M. Byrdin, N. Hoang, T. Ritz, K. Brettel, L.-O. Essen, G. T. J. van der Horst, A. Batschauer, M. Ahmad, The cryptochromes: Blue light photoreceptors in plants and animals. *Annu. Rev. Plant Biol.* **62**, 335–364 (2011).

4. K. Brettel, M. Byrdin, Reaction mechanisms of DNA photolyase. *Curr. Opin. Struct. Biol.* **20**, 693–701 (2010).

5. A. Sancar, Mechanisms of DNA repair by photolyase and excision nuclease (Nobel lecture). *Angew. Chem. Int. Ed. Engl.* **55**, 8502–8527 (2016).

6. L. O. Essen, T. Klar, Light-driven DNA repair by photolyases. *Cell. Mol. Life Sci.* **63**, 1266–1277 (2006).

7. T. Ritz, P. Thalau, J. B. Phillips, R. Wiltschko, W. Wiltschko, Resonance effects indicate a radical-pair mechanism for avian magnetic compass. *Nature* **429**, 177–180 (2004).

8. P. J. Hore, H. Mouritsen, The radical-pair mechanism of magnetoreception. *Annu. Rev. Biophys.* **45**, 299–344 (2016).

9. J. Xu, L. E. Jarocha, T. Zollitsch, M. Konowalczyk, K. B. Henbest, S. Richert, M. J. Golesworthy, J. Schmidt, V. Déjean, D. J. C. Sowood, M. Bassetto, J. Luo, J. R. Walton, J. Fleming, Y. Wei, T. L. Pitcher, G. Moise, M. Herrmann, H. Yin, H. Wu, R. Bartölke, S. J. Käsehagen, S. Horst, G. Dautaj, P. D. F. Murton, A. S. Gehrckens, Y. Chelliah, J. S. Takahashi, K.-W. Koch, S. Weber, I. A. Solov'yov, C. Xie, S. R. Mackenzie, C. R. Timmel, H. Mouritsen,

P. J. Hore, Magnetic sensitivity of cryptochrome 4 from a migratory songbird. *Nature* **594**, 535–540 (2021).

10. K. Schulten, C. E. Swenberg, A. Weiler, A biomagnetic sensory mechanism based on magnetic field modulated coherent electron spin motion. *Z. Phys. Chem.* **111**, 1–5 (1978).

11. B. Beel, K. Prager, M. Spexard, S. Sasso, D. Weiss, N. Müller, M. Heinnickel, D. Dewez, D. Ikoma, A. R. Grossman, T. Kottke, M. Mittag, A flavin binding cryptochrome photoreceptor responds to both blue and red light in Chlamydomonas reinhardtii. *Plant Cell* **24**, 2992–3008 (2012).

12. J. Petersen, A. Rredhi, J. Szyttenholm, S. Oldemeyer, T. Kottke, M. Mittag, The world of algae reveals a broad variety of cryptochrome properties and functions. *Front. Plant Sci.* **12**, 766509 (2021).

13. Y. Zou, S. Wenzel, N. Müller, K. Prager, E.-M. Jung, E. Kothe, T. Kottke, M. Mittag, An animal-like cryptochrome controls the *Chlamydomonas* sexual cycle. *Plant Physiol.* **174**, 1334–1347 (2017).

14. S. Franz, E. Ignatz, S. Wenzel, H. Zielosko, E. P. G. N. Putu, M. Maestre-Reyna, M.-D. Tsai, J. Yamamoto, M. Mittag, L.-O. Essen, Structure of the bifunctional cryptochrome aCRY from Chlamydomonas reinhardtii. *Nucleic Acids Res.* **46**, 8010–8022 (2018).

15. D. Nohr, S. Franz, R. Rodriguez, B. Paulus, L. O. Essen, S. Weber, E. Schleicher, Extended electron-transfer in animal cryptochromes mediated by a tetrad of aromatic amino acids. *Biophys. J.* **111**, 301–311 (2016).

16. S. Oldemeyer, S. Franz, S. Wenzel, L. O. Essen, M. Mittag, T. Kottke, Essential role of an unusually long-lived tyrosyl radical in the response to red light of the animal-like cryptochrome acry. *J. Biol. Chem.* **291**, 14062–14071 (2016).

17. R. Martin, F. Lacombat, A. Espagne, N. Dozova, P. Plaza, J. Yamamoto, P. Müller, K. Brettel, A. De La Lande, Ultrafast flavin photoreduction in an oxidized animal (6-4) photolyase through an unconventional tryptophan tetrad. *Phys. Chem. Chem. Phys.* **19**, 24493–24504 (2017).

18. D. Timmer, A. Frederiksen, D. C. Lünemann, A. R. Thomas, J. Xu, R. Bartölke, J. Schmidt, T. Kubař, A. De Sio, I. A. Solov'yov, H. Mouritsen, C. Lienau, Tracking the electron transfer cascade in European robin Cryptochrome 4 mutants. *J. Am. Chem. Soc.* **145**, 11566–11578 (2023).

19. A. K. Michael, J. L. Fribourgh, R. N. Van Gelder, C. L. Partch, Animal cryptochromes: Divergent roles in light perception, circadian timekeeping and beyond. *Photochem. Photobiol.* **93**, 128–140 (2017).

20. R. Zangl, S. Soravia, M. Saft, J. G. Löffler, J. Schulte, C. J. Rosner, J. Bredenbeck, L. O. Essen, N. Morgner, Time-resolved ion mobility mass spectrometry to solve conformational changes in a cryptochrome. *J. Am. Chem. Soc.* **146**, 14468–14478 (2024).

21. F. Lacombat, A. Espagne, N. Dozova, P. Plaza, P. Müller, K. Brettel, S. Franz-Badur, L. O. Essen, Ultrafast oxidation of a Tyrosine by proton-coupled electron transfer promotes light activation of an animal-like cryptochrome. *J. Am. Chem. Soc.* **141**, 13394–13409 (2019).

22. C. Kupitz, S. Basu, I. Grotjohann, R. Fromme, N. A. Zatsepin, K. N. Rendek, M. S. Hunter, R. L. Shoeman, T. A. White, D. Wang, D. James, J. H. Yang, D. E. Cobb, B. Reeder, R. G. Sierra, H. Liu, A. Barty, A. L. Aquila, D. Deponte, R. A. Kirian, S. Bari, J. J. Bergkamp, K. R. Beyerlein, M. J. Bogan, C. Caleman, T. C. Chao, C. E. Conrad, K. M. Davis, H. Fleckenstein, L. Galli, S. P. Hau-Riege, S. Kassemeyer, H. Laksmono, M. Liang, L. Lomb, S. Marchesini, A. V. Martin, M. Messerschmidt, D. Milathianaki, K. Nass, A. Ros, S. Roy-Chowdhury, K. Schmidt, M. Seibert, J. Steinbrener, F. Stellato, L. Yan, C. Yoon, T. A. Moore, A. L. Moore, Y. Pushkar, G. J. Williams, S. Boutet, R. B. Doak, U. Weierstall, M. Frank, H. N. Chapman, J. C. H. Spence, P. Fromme, Serial time-resolved crystallography of photosystem II using a femtosecond x-ray laser. *Nature* **513**, 261–265 (2014).

23. J. C. H. Spence, XFELs for structure and dynamics in biology. *IUCrJ* **4**, 322–339 (2017).

24. P. Fromme, XFELs open a new era in structural chemical biology. *Nat. Chem. Biol.* **11**, 895–899 (2015).

25. A. M. Orville, Recent results in time resolved serial femtosecond crystallography at XFELs. *Curr. Opin. Struct. Biol.* **65**, 193–208 (2020).

26. M. Maestre-Reyna, C. H. Yang, E. Nango, W. C. Huang, E. P. G. Ngurah Putu, W. J. Wu, P. H. Wang, S. Franz-Badur, M. Saft, H. J. Emmerich, H. Y. Wu, C. C. Lee, K. F. Huang, Y. K. Chang, J. H. Liao, J. H. Weng, W. Gad, C. W. Chang, A. H. Pang, M. Sugahara, S. Owada, Y. Hosokawa, Y. Joti, A. Yamashita, R. Tanaka, T. Tanaka, F. Luo, K. Tono, K. C. Hsu, S. Kiontke, I. Schapiro, R. Spadaccini, A. Royant, J. Yamamoto, S. Iwata, L. O. Essen, Y. Bessho, M. D. Tsai, Serial crystallography captures dynamic control of sequential electron and proton transfer events in a flavoenzyme. *Nat. Chem.* **14**, 677–685 (2022).

27. S. Franz-Badur, A. Penner, S. Straß, S. von Horsten, U. Linne, L. O. Essen, Structural changes within the bifunctional cryptochrome/photolyase *Cra*CRY upon blue light excitation. *Sci. Rep.* **9**, 9896 (2019).

28. M. Maestre-Reyna, P.-H. Wang, E. Nango, Y. Hosokawa, M. Saft, A. Furrer, C.-H. Yang, E. P. G. N. Putu, W.-J. Wu, H.-J. Emmerich, N. Caramello, S. Franz-Badur, C. Yang, S. Engilberge, M. Wranik, H. L. Glover, T. Weinert, H.-Y. Wu, C.-C. Lee, W.-C. Huang, K.-F. Huang, Y.-K. Chang, J.-H. Liao, J.-H. Weng, W. Gad, C.-W. Chang, A. H. Pang, K.-C. Yang, W.-T. Lin, Y.-C. Chang, D. Gashi, E. Beale, D. Ozerov, K. Nass, G. Knopp, P. J. M. Johnson, C. Cirelli, C. Milne, C. Bacellar, M. Sugahara, S. Owada, Y. Joti, A. Yamashita, R. Tanaka, T. Tanaka, F. Luo, K. Tono, W. Zarzycka, P. Müller, M. A. Alahmad, F. Bezold, V. Fuchs, P. Gnau, S. Kiontke, L. Korf, V. Reithofer, C. J. Rosner, E. M. Seiler, M. Watad, L. Werel, R. Spadaccini, J. Yamamoto, S. Iwata, D. Zhong, J. Standfuss, A. Royant, Y. Bessho, L.-O. Essen, M.-D. Tsai, Visualizing the DNA repair process by a photolyase at atomic resolution. *Science* **382**, eadd7795 (2023).

29. A. Cellini, M. K. Shankar, A. Nimmrich, L. A. Hunt, L. Monrroy, J. Mutisya, A. Furrer, E. V. Beale, M. Carrillo, T. N. Malla, P. Maj, L. Vrhovac, F. Dworkowski, C. Cirelli, P. J. M. Johnson, D. Ozerov, E. A. Stojković, L. Hammarström, C. Bacellar, J. Standfuss, M. Maj, M. Schmidt, T. Weinert, J. A. Ihalainen, W. Y. Wahlgren, S. Westenhoff, Directed ultrafast conformational changes accompany electron transfer in a photolyase as resolved by serial crystallography. *Nat. Chem.*, **16**, 624–632 (2024).

30. I. M. M. Wijaya, T. Domratcheva, T. Iwata, E. D. Getzoff, H. Kandori, Single hydrogen bond donation from Flavin N5 to proximal asparagine ensures FAD reduction in DNA photolyase. *J. Am. Chem. Soc.* **138**, 4368–4376 (2016).

31. T. Iwata, Y. Zhang, K. Hitomi, E. D. Getzoff, H. Kandori, Key dynamics of conserved asparagine in a cryptochrome/photolyase family protein by Fourier transform infrared spectroscopy. *Biochemistry* **49**, 8882–8891 (2010).

32. S. M. Harper, L. C. Neil, K. H. Gardner, Structural basis of a phototropin light switch. *Science* **301**, 1541–1544 (2003).

33. P. Li, H. Cheng, V. Kumar, C. S. Lupala, X. Li, Y. Shi, C. Ma, K. Joo, J. Lee, H. Liu, Y.-W. Tan, Direct experimental observation of blue-light-induced conformational change and intermolecular interactions of cryptochrome. *Commun. Biol.* **5**, 1103 (2022).

34. J. J. Goings, P. Li, Q. Zhu, S. Hammes-Schiffer, Formation of an unusual glutamine tautomer in a blue light using flavin photocycle characterizes the light-adapted state. *Proc. Natl. Acad. Sci. U.S.A.* **117**, 26626–26632 (2020).

35. T. Fujisawa, S. Masuda, Light-induced chromophore and protein responses and mechanical signal transduction of BLUF proteins. *Biophys. Rev.* **10**, 327–337 (2018).

36. N.-E. Christou, V. Apostolopoulou, D. V. M. Melo, M. Ruppert, A. Fadini, A. Henkel, J. Sprenger, D. Oberthuer, S. Günther, A. Pateras, A. R. Mashhour, O. M. Yefanov, M. Galchenkova, P. Y. A. Reinke, V. Kremling, T Emilie S Scheer, E. R. Lange, P. Middendorf, R. Schubert, E. De Zitter, K. Lumbao-Conradson, J. Herrmann, S. Rahighi, A. Kunavar, E. V. Beale, J. H. Beale, C. Cirelli, P. J. M. Johnson, F. Dworkowski, D. Ozerov, Q. Bertrand, M. Wranik, C. Bacellar, S. Bajt, S. Wakatsuki, J. A. Sellberg, N. Huse, D. Turk, H. N. Chapman, T. J. Lane, Time-resolved crystallography captures light-driven DNA repair. *Science* **382**, 1015–1020 (2023).

37. T. Kottke, A. Batschauer, M. Ahmad, J. Heberle, Blue-light-induced changes in arabidopsis cryptochrome 1 probed by FTIR difference spectroscopy. *Biochemistry* **45**, 2472–2479 (2006).

38. Y. Geisselbrecht, S. Frühwirth, C. Schroeder, A. J. J. Pierik, G. Klug, L.-O. Essen, CryB from Rhodobacter sphaeroides: A unique class of cryptochromes with new cofactors. *EMBO Rep.* **13**, 233–239 (2012).

39. B. D. Zoltowski, A. T. Vaidya, D. Top, J. Widom, M. W. Young, B. R. Crane, Structure of full-length Drosophila cryptochrome. *Nature* **480**, 396–399 (2011).

40. S. N. Nangle, C. Rosensweig, N. Koike, H. Tei, J. S. Takahashi, C. B. Green, N. Zheng, Molecular assembly of the period-cryptochrome circadian transcriptional repressor complex. *eLife* **3**, e03674 (2014).

41. I. Schmalen, S. Reischl, T. Wallach, R. Klemz, A. Grudziecki, J. R. Prabu, C. Benda, A. Kramer, E. Wolf, Interaction of circadian clock proteins CRY1 and PER2 is modulated by zinc binding and disulfide bond formation. *Cell* **157**, 1203–1215 (2014).

42. W. Xing, L. Busino, T. R. Hinds, S. T. Marionni, N. H. Saifee, M. F. Bush, M. Pagano, N. Zheng, SCF FBXL3 ubiquitin ligase targets cryptochromes at their cofactor pocket. *Nature* **496**, 64–68 (2013).

43. T. Ritz, S. Adem, K. Schulten, A model for photoreceptor-based magnetoreception in birds. *Biophys. J.* **78**, 707–718 (2000).

44. T. R. M. Barends, L. Foucar, A. Ardevol, K. Nass, A. Aquila, S. Botha, R. B. Doak, K. Falahati, E. Hartmann, M. Hilpert, M. Heinz, M. C. Hoffmann, J. Köfinger, J. E. Koglin, G. Kovacsova, M. Liang, D. Milathianaki, H. T. Lemke, J. Reinstein, C. M. Roome, R. L. Shoeman, G. J. Williams, I. Burghardt, G. Hummer, S. Boutet, I. Schlichting, Direct observation of ultrafast collective motions in CO myoglobin upon ligand dissociation. *Science* **350**, 445–450 (2015).

45. P. Nogly, T. Weinert, D. James, S. Carbajo, D. Ozerov, A. Furrer, D. Gashi, V. Borin, P. Skopintsev, K. Jaeger, K. Nass, P. Båth, R. Bosman, J. Koglin, M. Seaberg, T. Lane, D. Kekilli, S. Brünle, T. Tanaka, W. Wu, C. Milne, T. White, A. Barty, U. Weierstall, V. Panneels, E. Nango, S. Iwata, M. Hunter, I. Schapiro, G. Schertler, R. Neutze, J. Standfuss, Retinal

isomerization in bacteriorhodopsin captured by a femtosecond x-ray laser. *Science* **361**, eaat0094 (2018).

46. M. Yabashi, H. Tanaka, T. Ishikawa, Overview of the SACLA facility. *J. Synchrotron Radiat.* **22**, 477–484 (2015).

47. M. A. Rould, C. W. Carter Jr., Isomorphous difference methods. *Methods Enzymol.* **374**, 145–163 (2003).

48. M. Schmidt, Time-resolved macromolecular crystallography at pulsed x-ray sources. *Int. J. Mol. Sci.* **20**, 1401 (2019).

49. M. Sugahara, E. Mizohata, E. Nango, M. Suzuki, T. Tanaka, T. Masuda, R. Tanaka, T. Shimamura, Y. Tanaka, C. Suno, K. Ihara, D. Pan, K. Kakinouchi, S. Sugiyama, M. Murata, T. Inoue, K. Tono, C. Song, J. Park, T. Kameshima, T. Hatsui, Y. Joti, M. Yabashi, S. Iwata, Grease matrix as a versatile carrier of proteins for serial crystallography. *Nat. Methods* **12**, 61–63 (2014).

50. Y. Shimazu, K. Tono, T. Tanaka, Y. Yamanaka, T. Nakane, C. Mori, K. T. Kimura, T. Fujiwara, M. Sugahara, R. Tanaka, R. B. Doak, T. Shimamura, S. Iwata, E. Nango, M. Yabashi, High-viscosity sample-injection device for serial femtosecond crystallography at atmospheric pressure. *J. Appl. Cryst.* **52**, 1280–1288 (2019).

51. D. von Stetten, T. Giraud, P. Carpentier, F. Sever, M. Terrien, F. Dobias, D. H. Juers, D. Flot, C. Mueller-Dieckmann, G. A. Leonard, D. De Sanctis, A. Royant, In crystallo optical spectroscopy (*ic*OS) as a complementary tool on the macromolecular crystallography beamlines of the ESRF. *Acta Crystallogr. D Biol. Crystallogr.* **71**, 15–26 (2015).

52. K. Tono, E. Nango, M. Sugahara, C. Song, J. Park, T. Tanaka, R. Tanaka, Y. Joti, T. Kameshima, S. Ono, T. Hatsui, E. Mizohata, M. Suzuki, T. Shimamura, Y. Tanaka, S. Iwata, M. Yabashi, Diverse application platform for hard x-ray diffraction in SACLA (DAPHNIS): Application to serial protein crystallography using an x-ray free-electron laser. *J. Synchrotron Radiat.* **22**, 532–537 (2015).

53. T. Kameshima, S. Ono, T. Kudo, K. Ozaki, Y. Kirihara, K. Kobayashi, Y. Inubushi, M. Yabashi, T. Horigome, A. Holland, K. Holland, D. Burt, H. Murao, T. Hatsui, Development of an x-ray pixel detector with multi-port charge-coupled device for x-ray free-electron laser experiments. *Rev. Sci. Instrum.* **85**, 033110 (2014).

54. T. Nakane, Y. Joti, K. Tono, M. Yabashi, E. Nango, S. Iwata, R. Ishitani, O. Nureki, Data processing pipeline for serial femtosecond crystallography at SACLA. *J. Appl. Cryst.* **49**, 1035–1041 (2016).

55. T. A. White, Processing serial crystallography data with crystFEL: A step-by-step guide. *Acta Crystallogr. D Struct. Biol.* **75**, 219–233 (2019).

56. T. A. White, R. A. Kirian, A. V. Martin, A. Aquila, K. Nass, A. Barty, H. N. Chapman, *CrystFEL*: A software suite for snapshot serial crystallography. *J. Appl. Cryst.* **45**, 335–341 (2012).

57. M. D. Winn, C. C. Ballard, K. D. Cowtan, E. J. Dodson, P. Emsley, P. R. Evans, R. M. Keegan, E. B. Krissinel, A. G. W. Leslie, A. McCoy, S. J. McNicholas, G. N. Murshudov, N. S. Pannu, E. A. Potterton, H. R. Powell, R. J. Read, A. Vagin, K. S. Wilson, Overview of the CCP4 suite and current developments. *Acta Crystallogr. D Biol. Crystallogr.* **67**, 235–242 (2011).

58. A. J. McCoy, R. W. Grosse-Kunstleve, P. D. Adams, M. D. Winn, L. C. Storoni, R. J. Read, Phaser crystallographic software. *J. Appl. Cryst.* **40**, 658–674 (2007).

59. D. Liebschner, P. V Afonine, M. L. Baker, G. Bunkoczi, V. B. Chen, T. I. Croll, B. Hintze, L. W. Hung, S. Jain, A. J. McCoy, N. W. Moriarty, R. D. Oeffner, B. K. Poon, M. G. Prisant, R. J. Read, J. S. Richardson, D. C. Richardson, M. D. Sammito, O. V Sobolev, D. H. Stockwell, T. C. Terwilliger, A. G. Urzhumtsev, L. L. Videau, C. J. Williams, P. D. Adams, Macromolecular structure determination using X-rays, neutrons and electrons: Recent developments in Phenix. *Acta Crystallogr. D Struct. Biol.* **75**, 861–877 (2019).

60. P. V Afonine, R. W. Grosse-Kunstleve, N. Echols, J. J. Headd, N. W. Moriarty, M. Mustyakimov, T. C. Terwilliger, A. Urzhumtsev, P. H. Zwart, P. D. Adams, Towards automated

crystallographic structure refinement with phenix.refine. *Acta Crystallogr. D Biol. Crystallogr.* **68**, 352–367 (2012).

61. P. D. Adams, P. V. Afonine, G. Bunkóczi, V. B. Chen, I. W. Davis, N. Echols, J. J. Headd, L. W. Hung, G. J. Kapral, R. W. Grosse-Kunstleve, A. J. McCoy, N. W. Moriarty, R. Oeffner, R. J. Read, D. C. Richardson, J. S. Richardson, T. C. Terwilliger, P. H. Zwart, PHENIX: A comprehensive Python-based system for macromolecular structure solution. *Acta Crystallogr. D Biol. Crystallogr.* **66**, 213–221 (2010).

62. P. Emsley, B. Lohkamp, W. G. Scott, K. Cowtan, Features and development of Coot. *Acta Crystallogr. D Biol. Crystallogr.* **66**, 486–501 (2010).

63. G. N. Murshudov, P. Skubák, A. A. Lebedev, N. S. Pannu, R. A. Steiner, R. A. Nicholls, M. D. Winn, F. Long, A. A. Vagin, REFMAC5 for the refinement of macromolecular crystal structures. *Acta Crystallogr. D Biol. Crystallogr.* **67**, 355–367 (2011).

64. M. Carrillo, S. Pandey, J. Sanchez, M. Noda, I. Poudyal, L. Aldama, T. N. Malla, E. Claesson, W. Y. Wahlgren, D. Feliz, V. Šrajer, M. Maj, L. Castillon, S. Iwata, E. Nango, R. Tanaka, T. Tanaka, L. Fangjia, K. Tono, S. Owada, S. Westenhoff, E. A. Stojković, M. Schmidt, High-resolution crystal structures of transient intermediates in the phytochrome photocycle. *Structure* **29**, 743–754.e4 (2021).

65. U. K. Genick, G. E. O. Borgstahl, K. Ng, Z. Ren, C. Pradervand, P. M. Burke, V. Šrajer, T.-Y. Teng, W. Schildkamp, D. E. McRee, K. Moffat, E. D. Getzoff, Structure of a protein photocycle intermediate by millisecond time-resolved crystallography. *Science* **275**, 1471–1475 (1997).

66. S. Engilberge, N. Caramello, S. Bukhdruker, M. Byrdin, T. Giraud, P. Jacquet, D. Scortani, R. Biv, H. Gonzalez, A. Broquet, P. Van Der Linden, S. L. Rose, D. Flot, T. Balandin, V. Gordeliy, J. M. Lahey-Rudolph, M. Roessle, D. De Sanctis, G. A. Leonard, C. Mueller-Dieckmann, A. Royant, The TR-icOS setup at the ESRF: Time-resolved microsecond UV-Vis absorption spectroscopy on protein crystals. *Acta Crystallogr. D Struct. Biol.* **80**, 16–25 (2024).

67. F. S. N. Dworkowski, M. A. Hough, G. Pompidor, M. R. Fuchs, Challenges and solutions for the analysis of in situ, in crystallo micro-spectrophotometric data. *Acta Crystallogr. D Biol. Crystallogr.* **71**, 27–35 (2015).

68. P. Müller, J. Yamamoto, R. Martin, S. Iwai, K. Brettel, Discovery and functional analysis of a 4th electron-transferring tryptophan conserved exclusively in animal cryptochromes and (6-4) photolyases. *Chem. Commun.* **51**, 15502–15505 (2015).

69. M. Schmidt, S. Rajagopal, Z. Ren, K. Moffat, Application of singular value decomposition to the analysis of time-resolved macromolecular x-ray data. *Biophys. J.* **84**, 2112–2129 (2003).

70. M. Schmidt, R. Pahl, V. Srajer, S. Anderson, Z. Ren, H. Ihee, S. Rajagopal, K. Moffat, Protein kinetics: Structures of intermediates and reaction mechanism from time-resolved x-ray data. *Proc. Natl. Acad. Sci. U.S.A.* **101**, 4799–4804 (2004).

71. T. E. Wales, K. E. Fadgen, G. C. Gerhardt, J. R. Engen, High-speed and high-resolution UPLC separation at zero degrees celsius. *Anal. Chem.* **80**, 6815–6820 (2008).

72. S. J. Geromanos, J. P. C. Vissers, J. C. Silva, C. A. Dorschel, G. Z. Li, M. V. Gorenstein, R. H. Bateman, J. I. Langridge, The detection, correlation, and comparison of peptide precursor and product ions from data independent LC-MS with data dependant LC-MS/MS. *Proteomics* **9**, 1683–1695 (2009).

73. G.-Z. Li, J. P. C. Vissers, J. C. Silva, D. Golick, M. V. Gorenstein, S. J. Geromanos, Database searching and accounting of multiplexed precursor and product ion spectra from the data independent analysis of simple and complex peptide mixtures. *Proteomics* **9**, 1696–1719 (2009).

74. M. Osorio-Valeriano, F. Altegoer, W. Steinchen, S. Urban, Y. Liu, G. Bange, M. Thanbichler, ParB-type DNA segregation proteins are CTP-dependent molecular switches. *Cell* **179**, 1512–1524.e15 (2019).

75. L. Schrödinger, W. DeLano, The PyMOL Molecular Graphics System, Version 3.0 Schrödinger LLC.

76. G. Brändén, R. Neutze, Advances and challenges in time-resolved macromolecular crystallography. *Science* **373**, eaba0954 (2021).

77. R. Neutze, R. J. D. Miller, Energetic laser pulses alter outcomes of x-ray studies of proteins. *Nature* **626**, 720–722 (2024).

78. T. R. M. Barends, A. Gorel, S. Bhattacharyya, G. Schirò, C. Bacellar, C. Cirelli, J. P. Colletier, L. Foucar, M. L. Grünbein, E. Hartmann, M. Hilpert, J. M. Holton, P. J. M. Johnson, M. Kloos, G. Knopp, B. Marekha, K. Nass, G. Nass Kovacs, D. Ozerov, M. Stricker, M. Weik, R. B. Doak, R. L. Shoeman, C. J. Milne, M. Huix-Rotllant, M. Cammarata, I. Schlichting, Influence of pump laser fluence on ultrafast myoglobin structural dynamics. *Nature* **626**, 905–911 (2024).

79. Q. Bertrand, P. Nogly, E. Nango, D. Kekilli, G. Khusainov, A. Furrer, D. James, F. Dworkowski, P. Skopintsev, S. Mous, I. Martiel, P. Börjesson, G. Ortolani, C. Y. Huang, M. Kepa, D. Ozerov, S. Brünle, V. Panneels, T. Tanaka, R. Tanaka, K. Tono, S. Owada, P. J. M. Johnson, K. Nass, G. Knopp, C. Cirelli, C. Milne, G. Schertler, S. Iwata, R. Neutze, T. Weinert, J. Standfuss, Structural effects of high laser power densities on an early bacteriorhodopsin photocycle intermediate. *Nat. Commun.* **15**, 10278 (2024).

80. A. Vallejos, G. Katona, R. Neutze, Appraising protein conformational changes by resampling time-resolved serial x-ray crystallography data. *Struct. Dyn.* **11**, 044302 (2024).
